# Supplementary material for: Engaging Learners Through Modules in Quality Improvement and Patient Safety
Source: MedEdPORTAL. 2016 Oct 13;12:10482. doi: 10.15766/mep_2374-8265.10482 (PMC6440404; doi:10.15766/mep_2374-8265.10482)
Supplement: Supplementary file 1 — A. Instructor's Guide.docx B. PowerPoint Talking Points.docx C. Knowledge Survey.docx D. Attitude Survey Questions.docx E. Fundamentals of QI.pptx F. Fundamentals of Patient Safety.ppt G. Evidence-Based Practice and QI Improvement Research.pptx H. QI and PS Potpourri.pptx [file mep-12-10482-s001.zip › H. QI and PS Potpourri.pptx]

## Slide 1
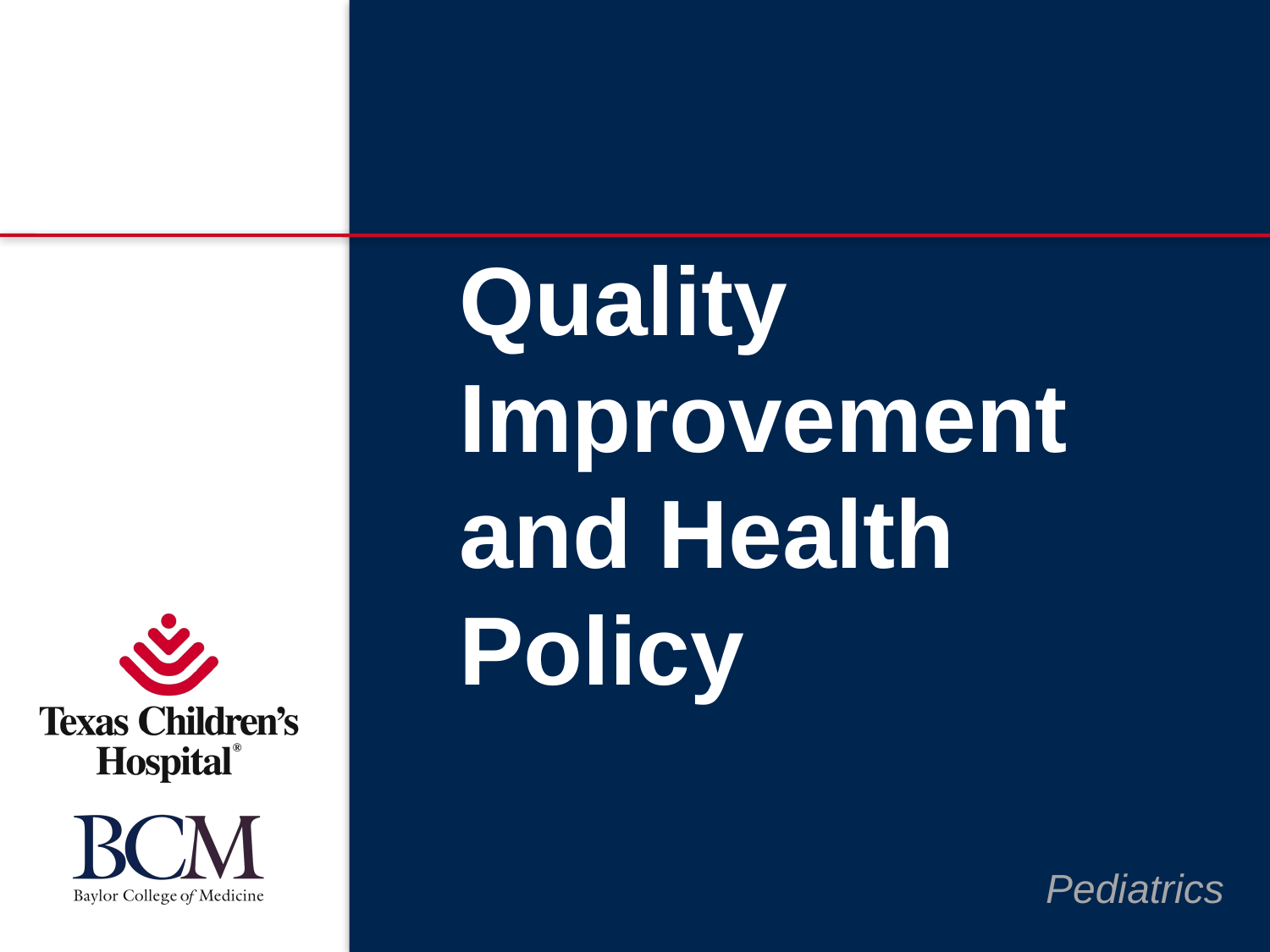

# Quality Improvement and Health Policy

## Slide 2
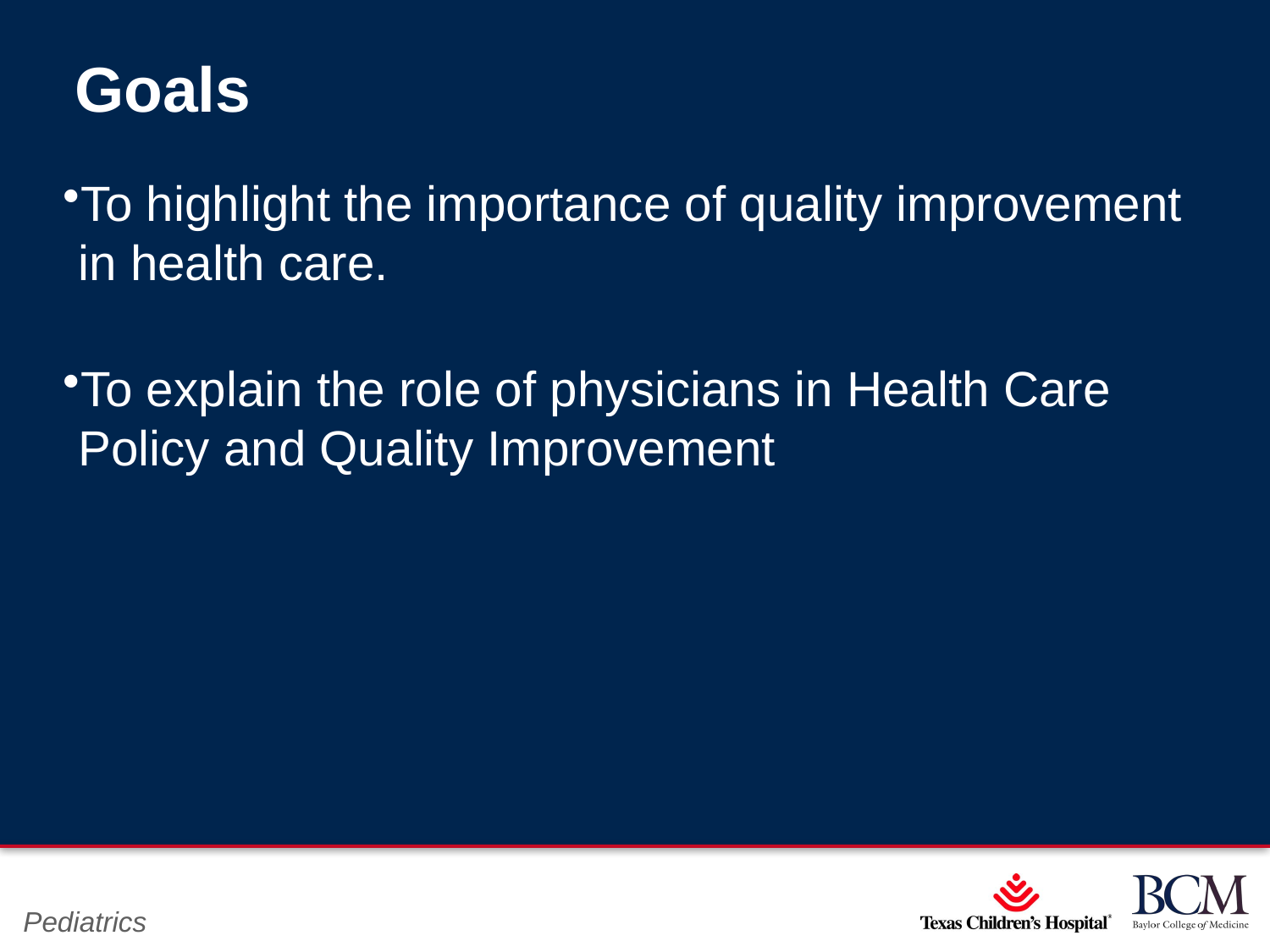

# Goals
To highlight the importance of quality improvement in health care.
To explain the role of physicians in Health Care Policy and Quality Improvement

## Slide 3
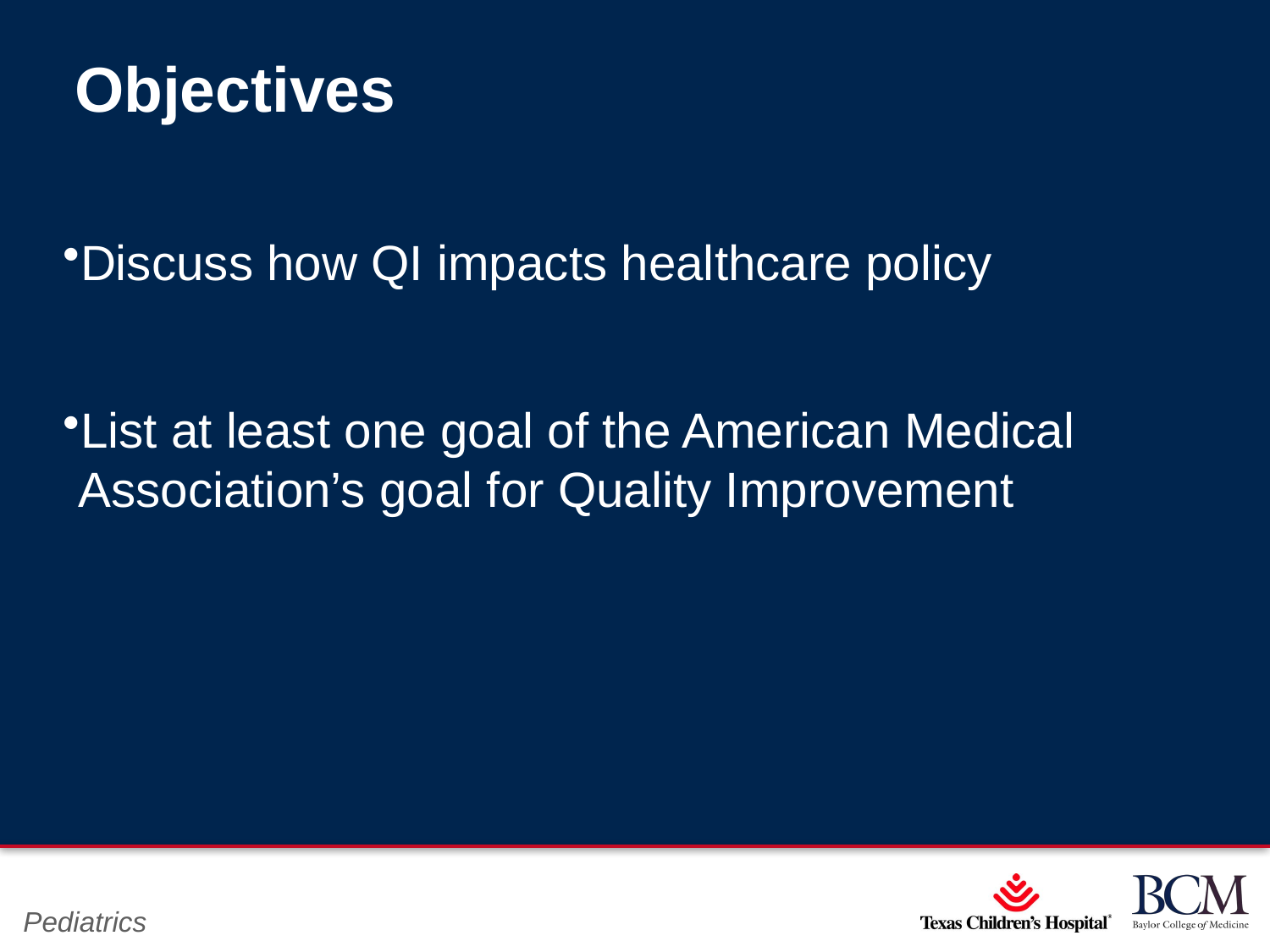

# Objectives
Discuss how QI impacts healthcare policy
List at least one goal of the American Medical Association’s goal for Quality Improvement

## Slide 4
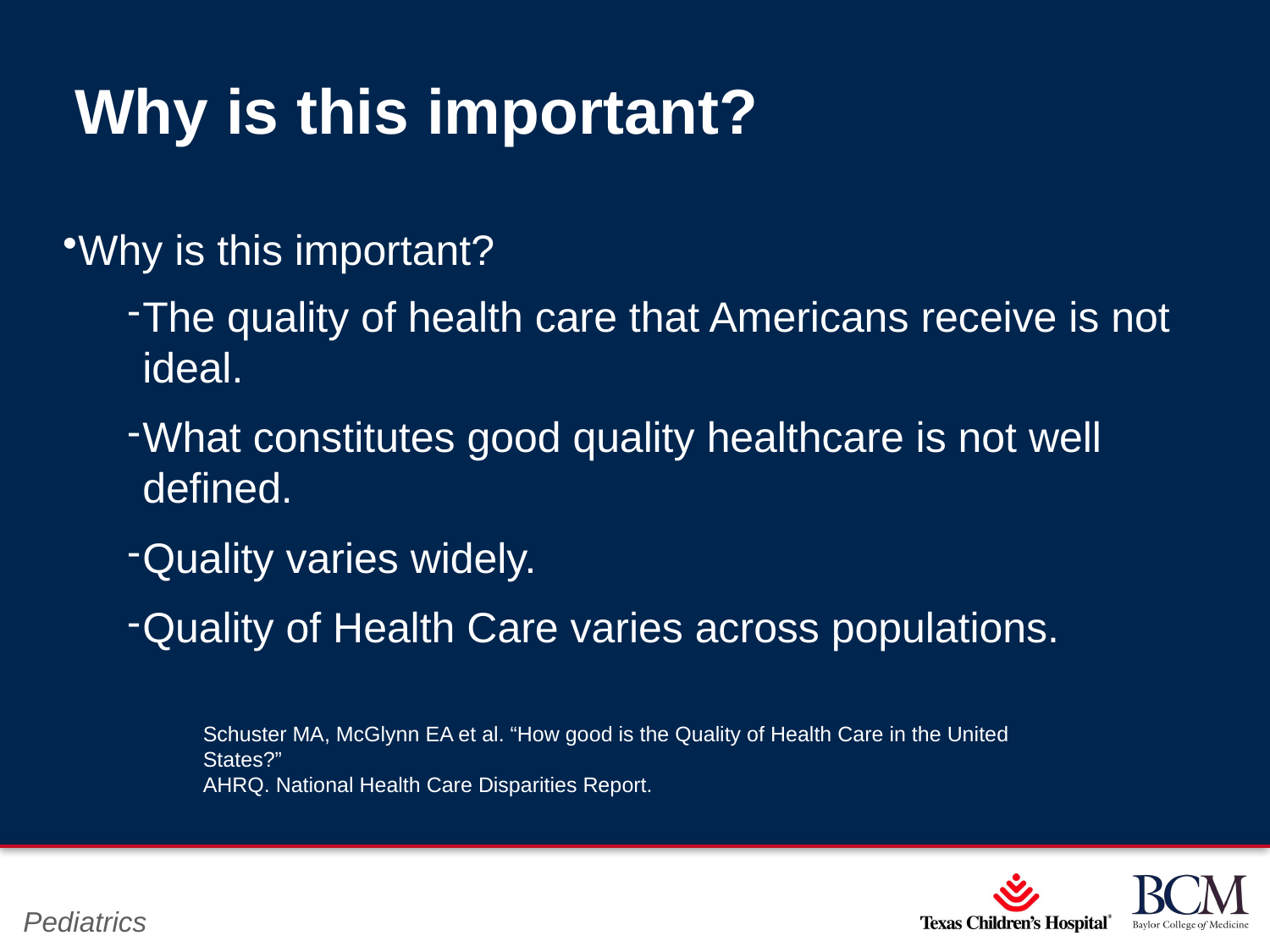

# Why is this important?
Why is this important?
The quality of health care that Americans receive is not ideal.
What constitutes good quality healthcare is not well defined.
Quality varies widely.
Quality of Health Care varies across populations.
Schuster MA, McGlynn EA et al. “How good is the Quality of Health Care in the United States?”
AHRQ. National Health Care Disparities Report.

## Slide 5
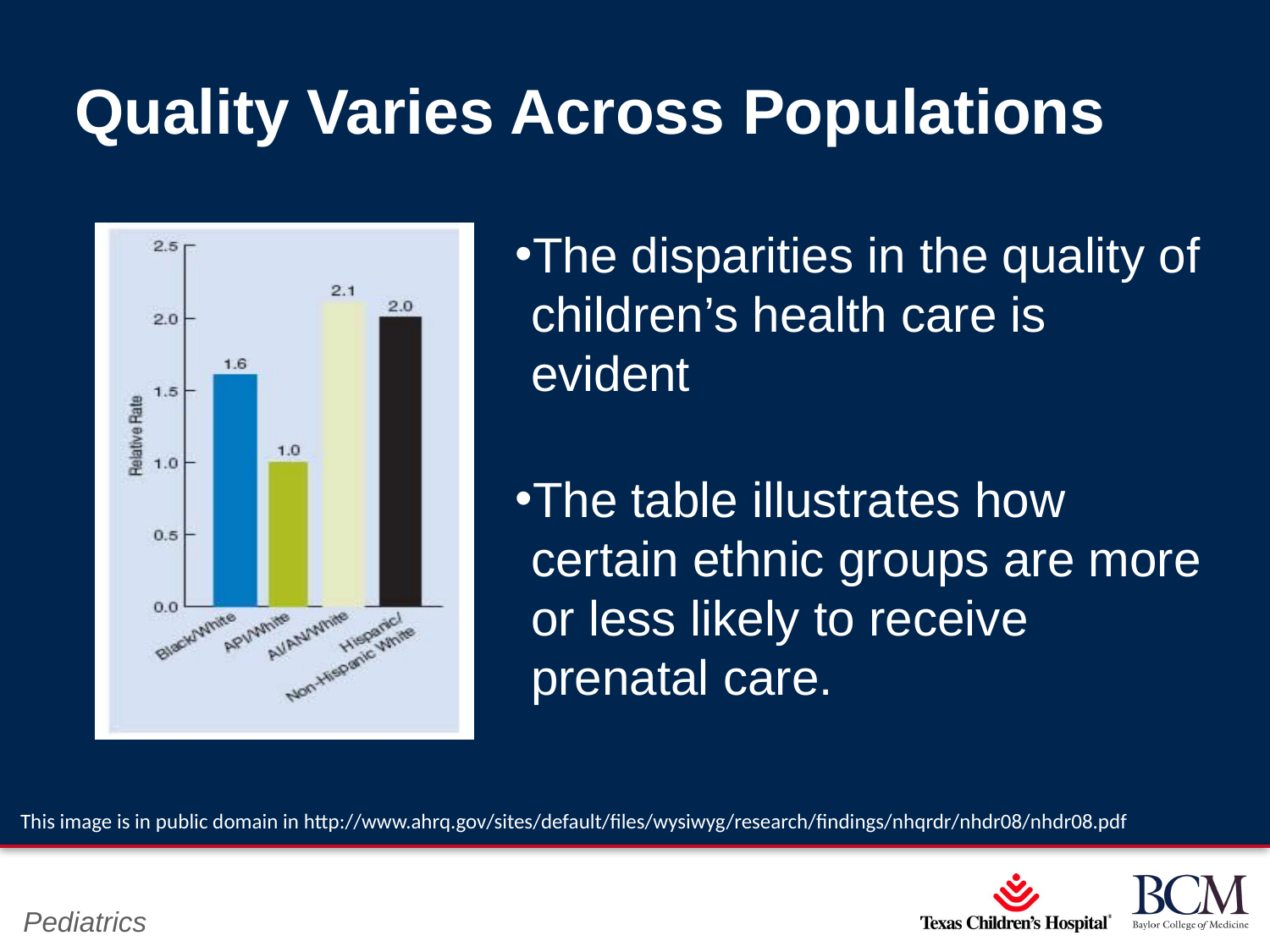

# Quality Varies Across Populations
The disparities in the quality of children’s health care is evident
The table illustrates how certain ethnic groups are more or less likely to receive prenatal care.
This image is in public domain in http://www.ahrq.gov/sites/default/files/wysiwyg/research/findings/nhqrdr/nhdr08/nhdr08.pdf

## Slide 6
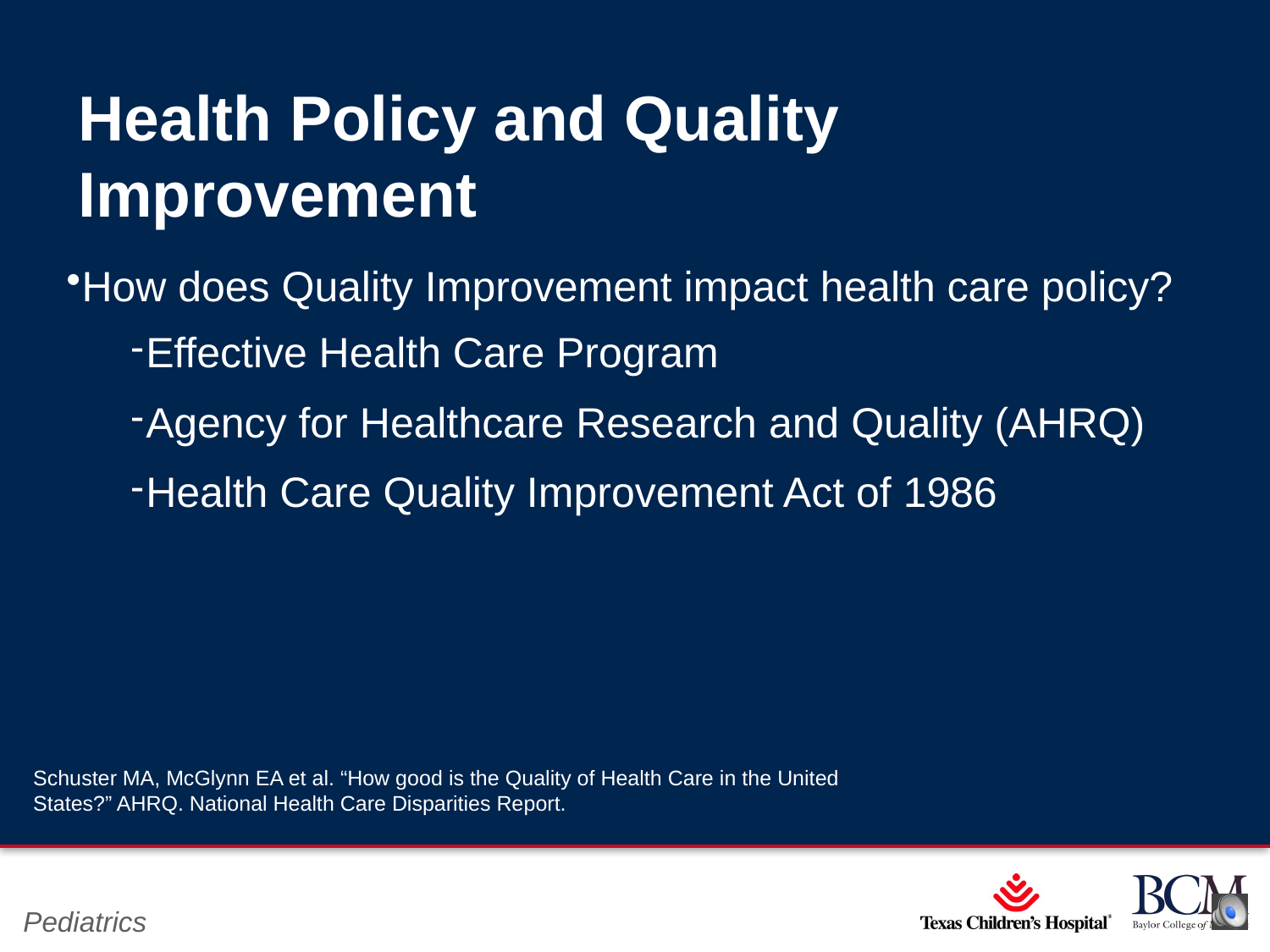

# Health Policy and Quality Improvement
How does Quality Improvement impact health care policy?
Effective Health Care Program
Agency for Healthcare Research and Quality (AHRQ)
Health Care Quality Improvement Act of 1986
Schuster MA, McGlynn EA et al. “How good is the Quality of Health Care in the United States?” AHRQ. National Health Care Disparities Report.

## Slide 7
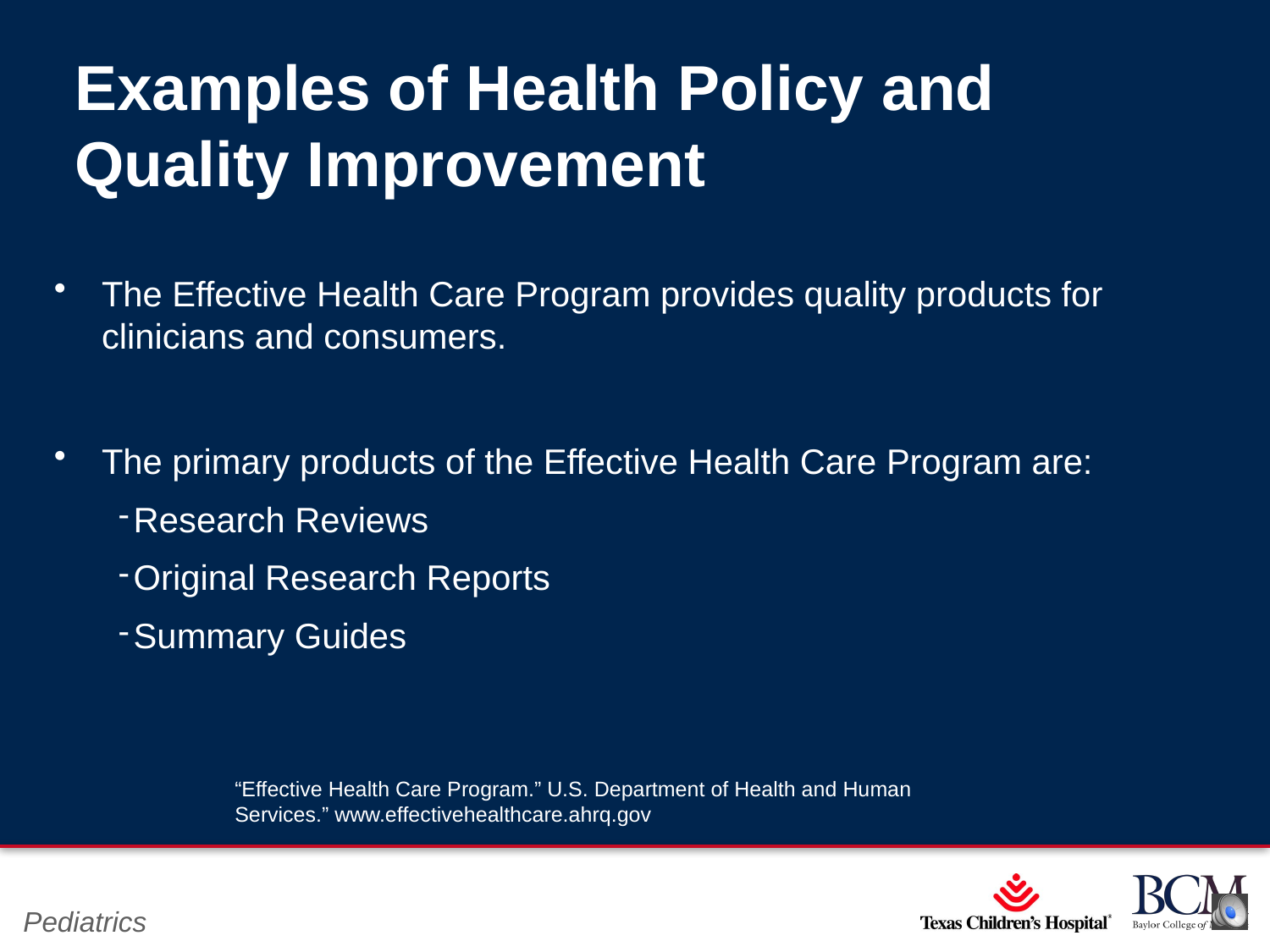

# Examples of Health Policy and Quality Improvement
The Effective Health Care Program provides quality products for clinicians and consumers.
The primary products of the Effective Health Care Program are:
Research Reviews
Original Research Reports
Summary Guides
“Effective Health Care Program.” U.S. Department of Health and Human Services.” www.effectivehealthcare.ahrq.gov

## Slide 8
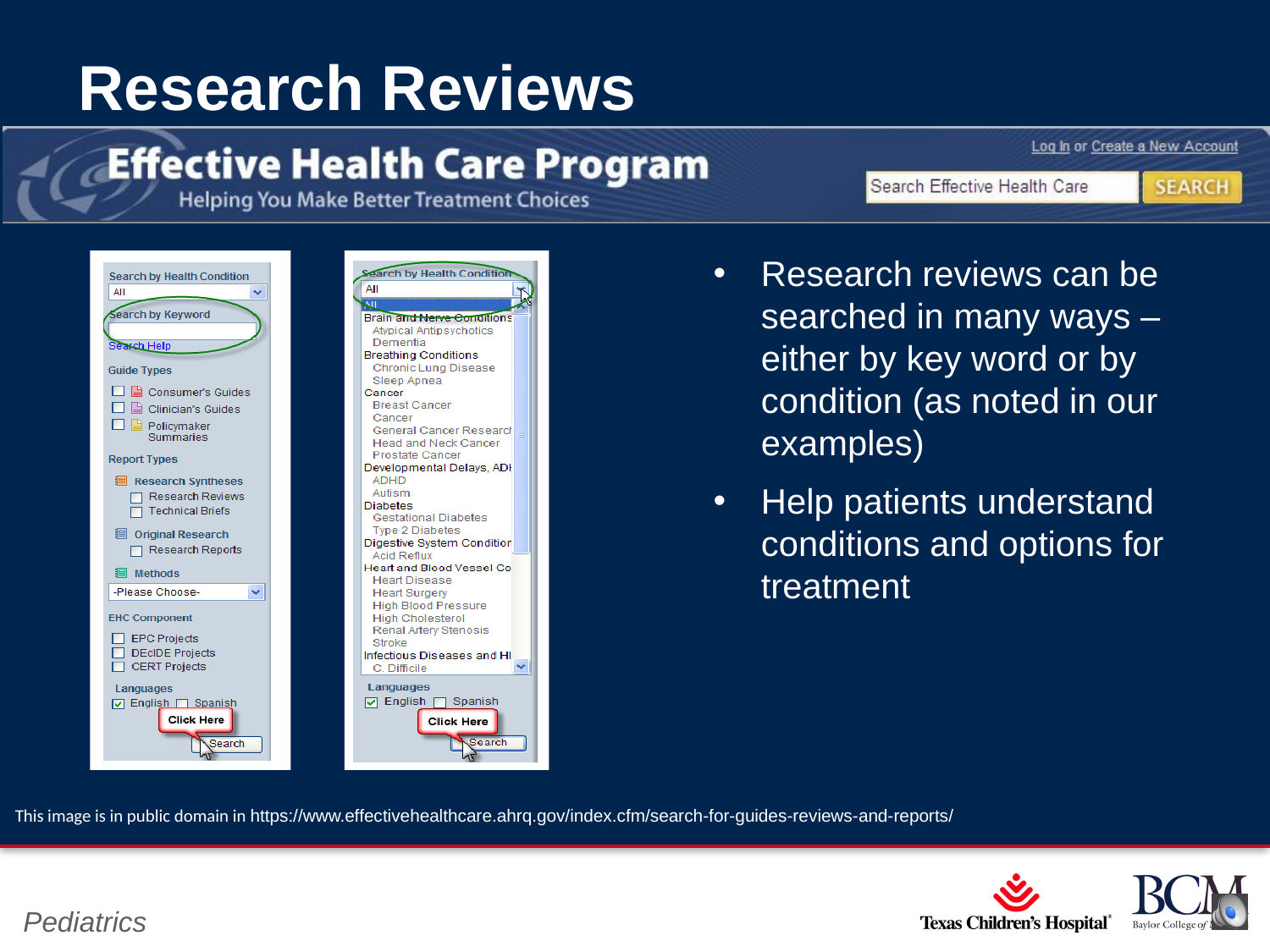

# Research Reviews
Research reviews can be searched in many ways – either by key word or by condition (as noted in our examples)
Help patients understand conditions and options for treatment
This image is in public domain in https://www.effectivehealthcare.ahrq.gov/index.cfm/search-for-guides-reviews-and-reports/

## Slide 9
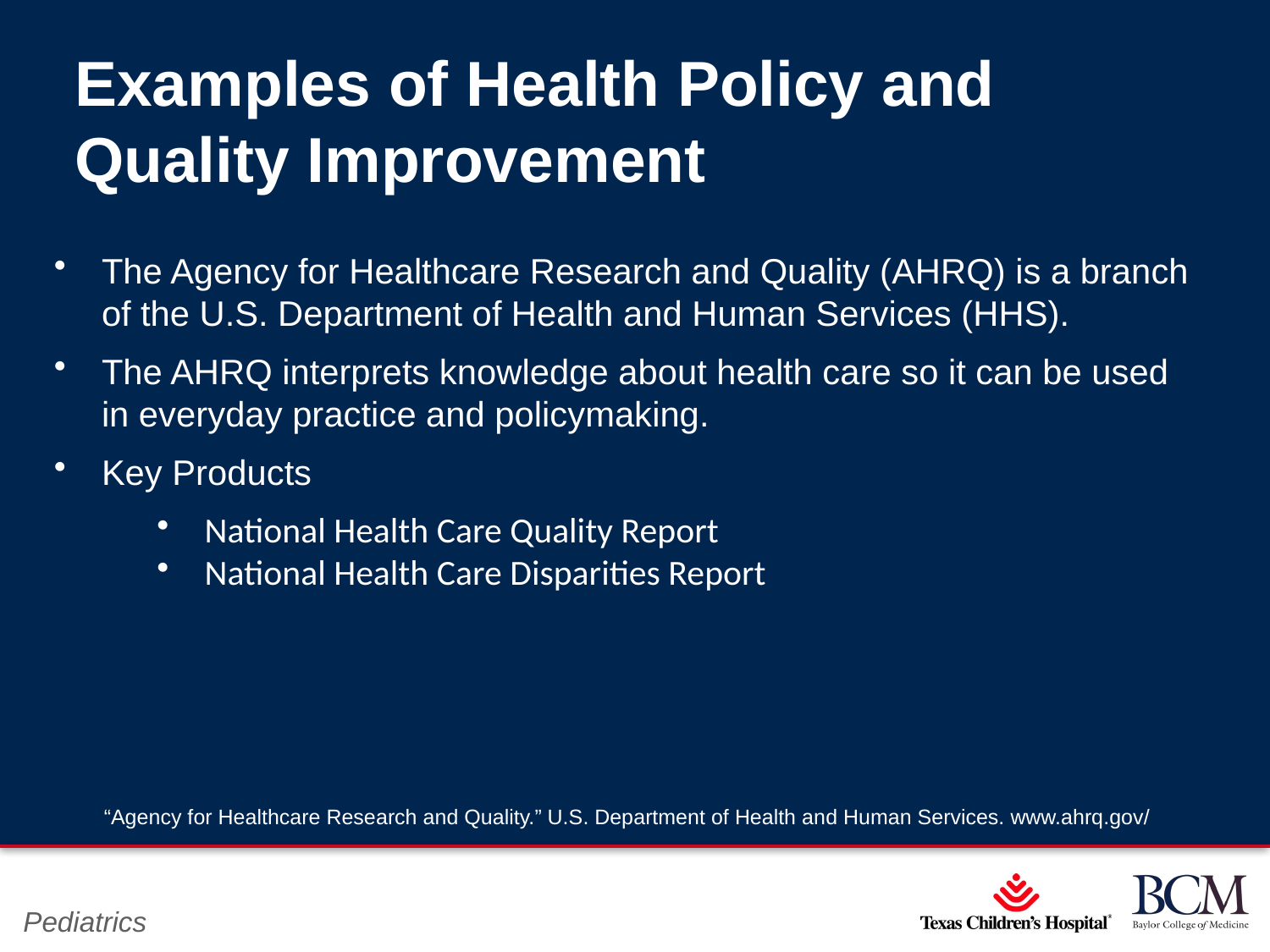

Examples of Health Policy and Quality Improvement
The Agency for Healthcare Research and Quality (AHRQ) is a branch of the U.S. Department of Health and Human Services (HHS).
The AHRQ interprets knowledge about health care so it can be used in everyday practice and policymaking.
Key Products
National Health Care Quality Report
National Health Care Disparities Report
“Agency for Healthcare Research and Quality.” U.S. Department of Health and Human Services. www.ahrq.gov/

## Slide 10
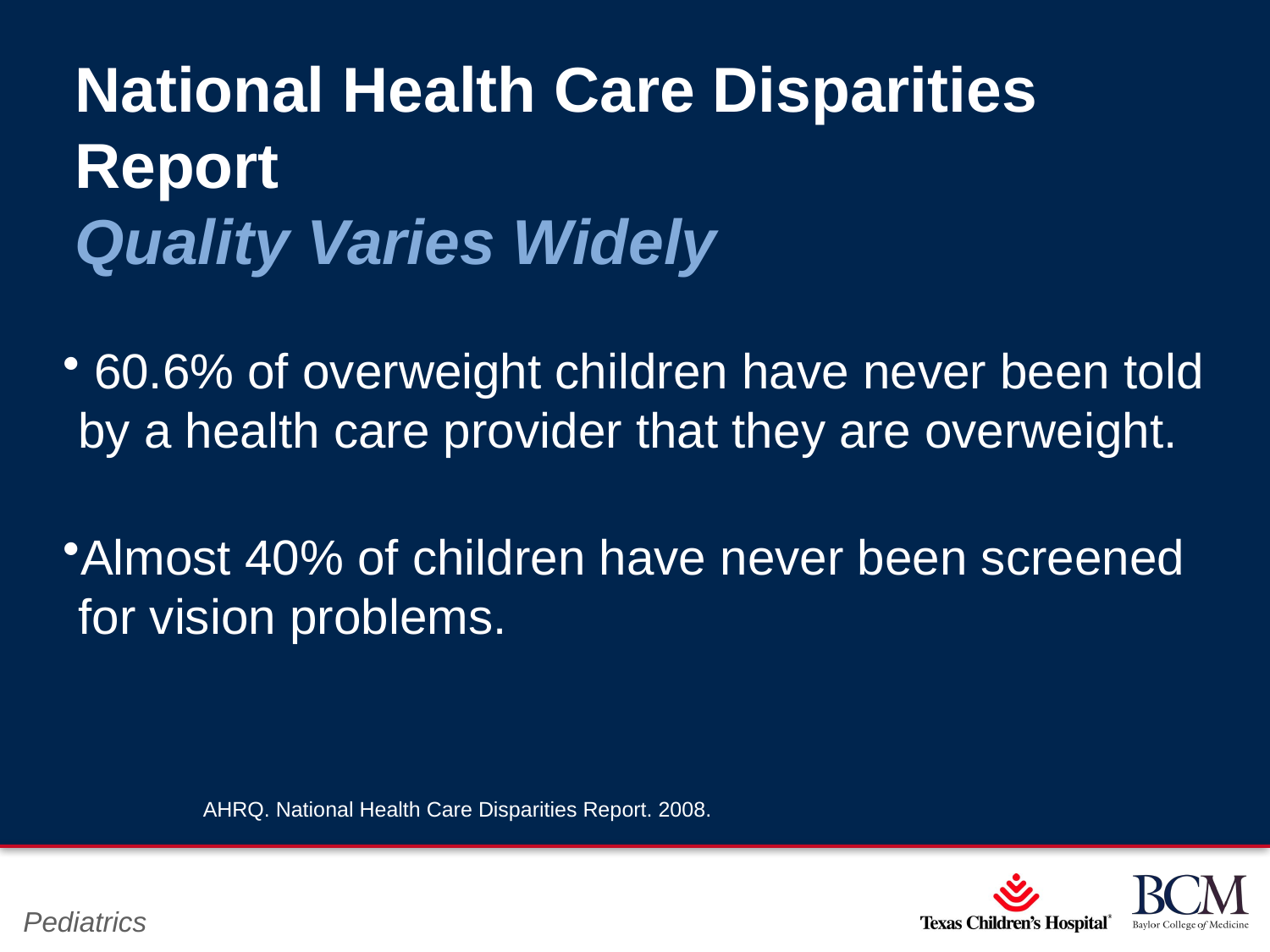

# National Health Care Disparities Report Quality Varies Widely
 60.6% of overweight children have never been told by a health care provider that they are overweight.
Almost 40% of children have never been screened for vision problems.
AHRQ. National Health Care Disparities Report. 2008.

## Slide 11
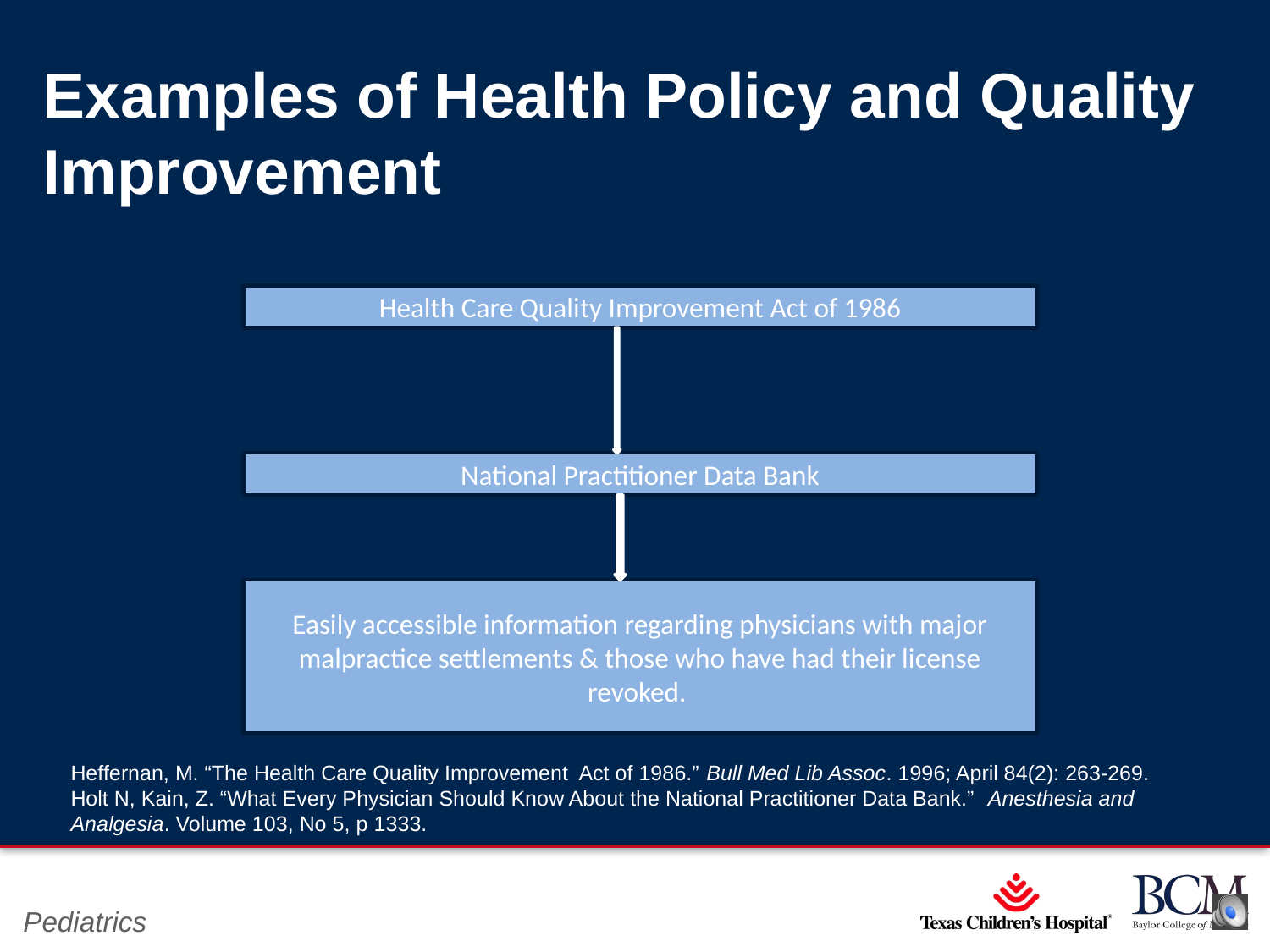

# Examples of Health Policy and Quality Improvement
Health Care Quality Improvement Act of 1986
National Practitioner Data Bank
Easily accessible information regarding physicians with major malpractice settlements & those who have had their license revoked.
Heffernan, M. “The Health Care Quality Improvement Act of 1986.” Bull Med Lib Assoc. 1996; April 84(2): 263-269.
Holt N, Kain, Z. “What Every Physician Should Know About the National Practitioner Data Bank.” Anesthesia and Analgesia. Volume 103, No 5, p 1333.

## Slide 12
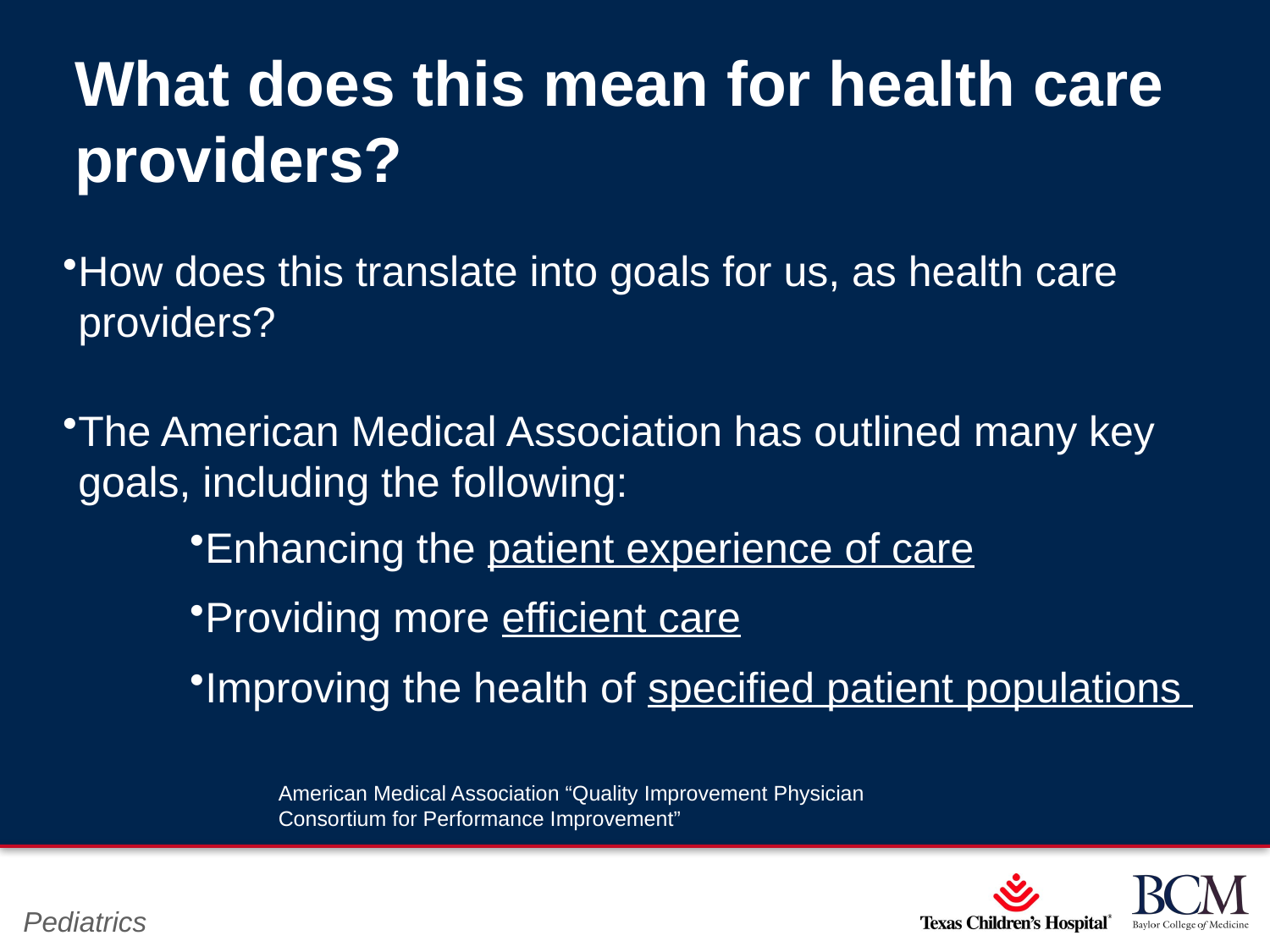

# What does this mean for health care providers?
How does this translate into goals for us, as health care providers?
The American Medical Association has outlined many key goals, including the following:
Enhancing the patient experience of care
Providing more efficient care
Improving the health of specified patient populations
American Medical Association “Quality Improvement Physician Consortium for Performance Improvement”

## Slide 13
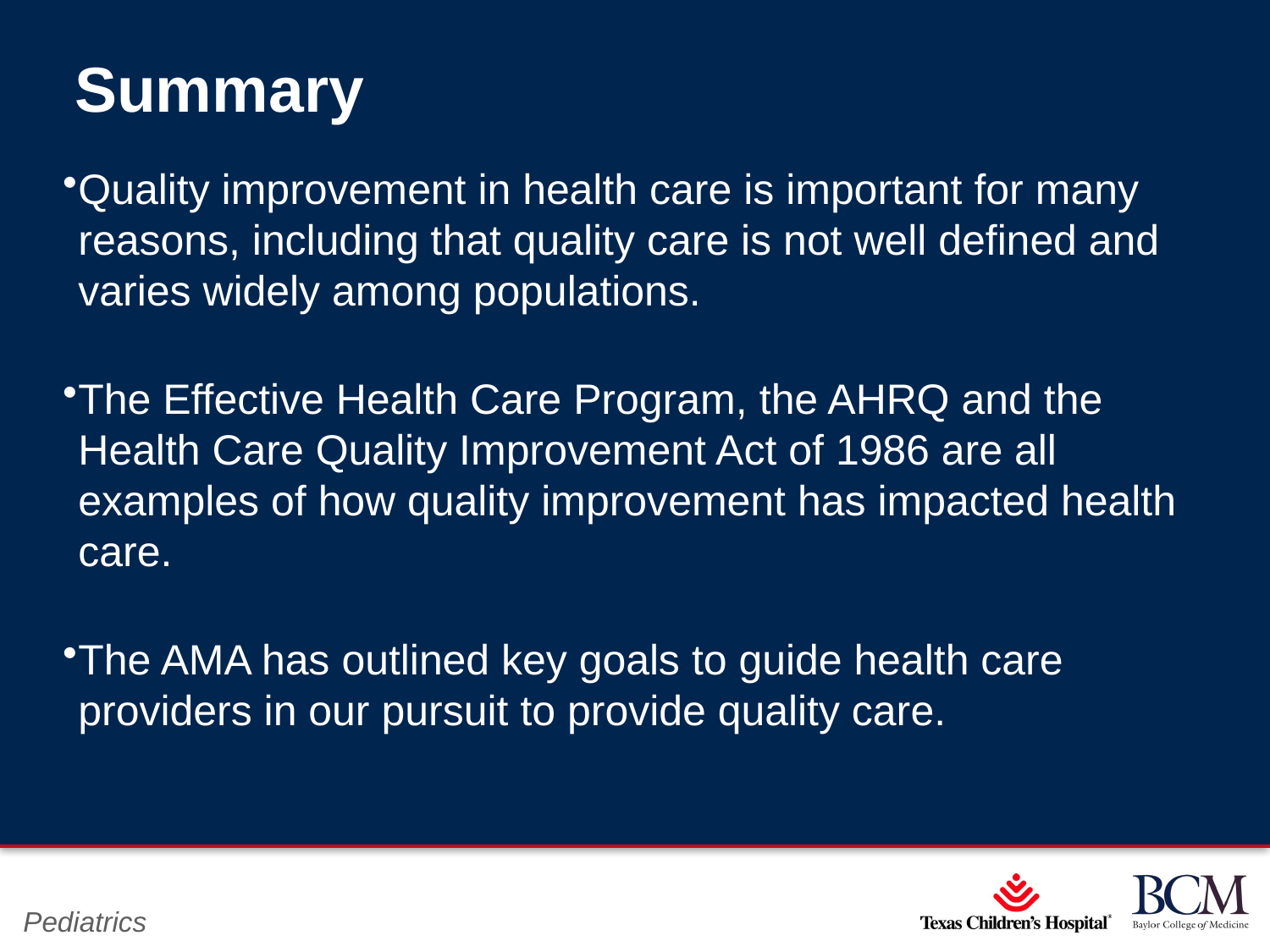

# Summary
Quality improvement in health care is important for many reasons, including that quality care is not well defined and varies widely among populations.
The Effective Health Care Program, the AHRQ and the Health Care Quality Improvement Act of 1986 are all examples of how quality improvement has impacted health care.
The AMA has outlined key goals to guide health care providers in our pursuit to provide quality care.

## Slide 14
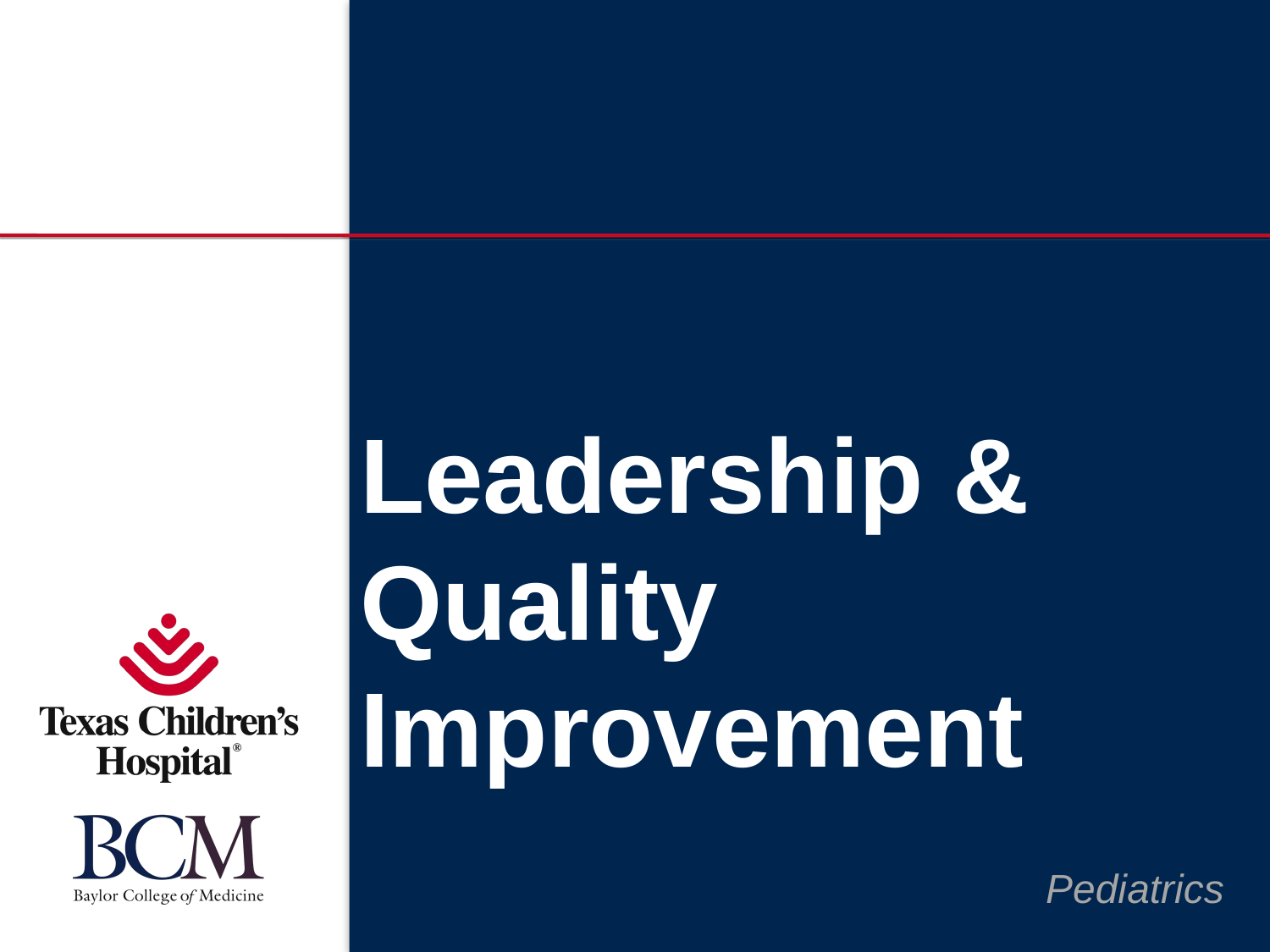

# Leadership & Quality Improvement

## Slide 15
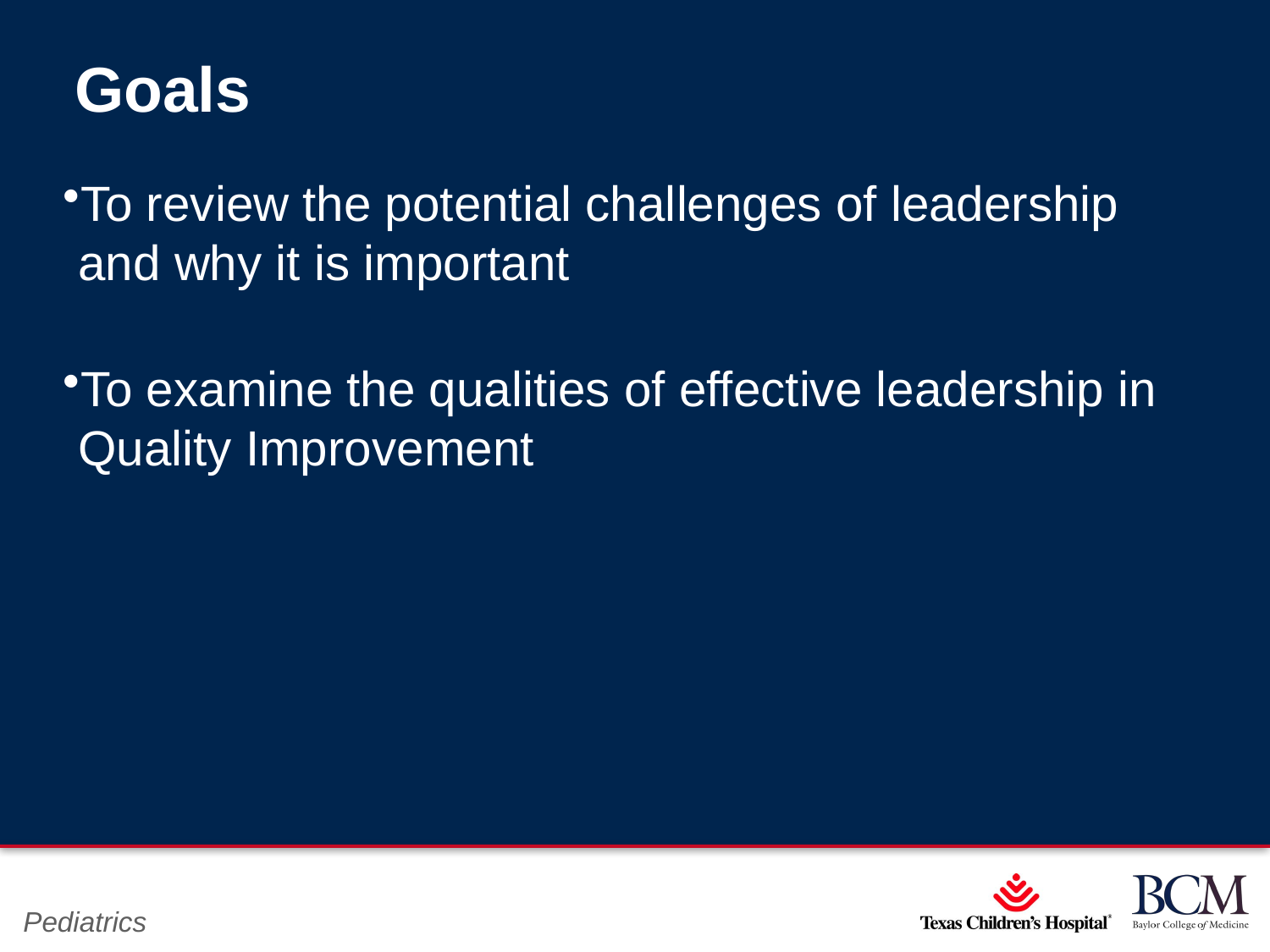

# Goals
To review the potential challenges of leadership and why it is important
To examine the qualities of effective leadership in Quality Improvement

## Slide 16
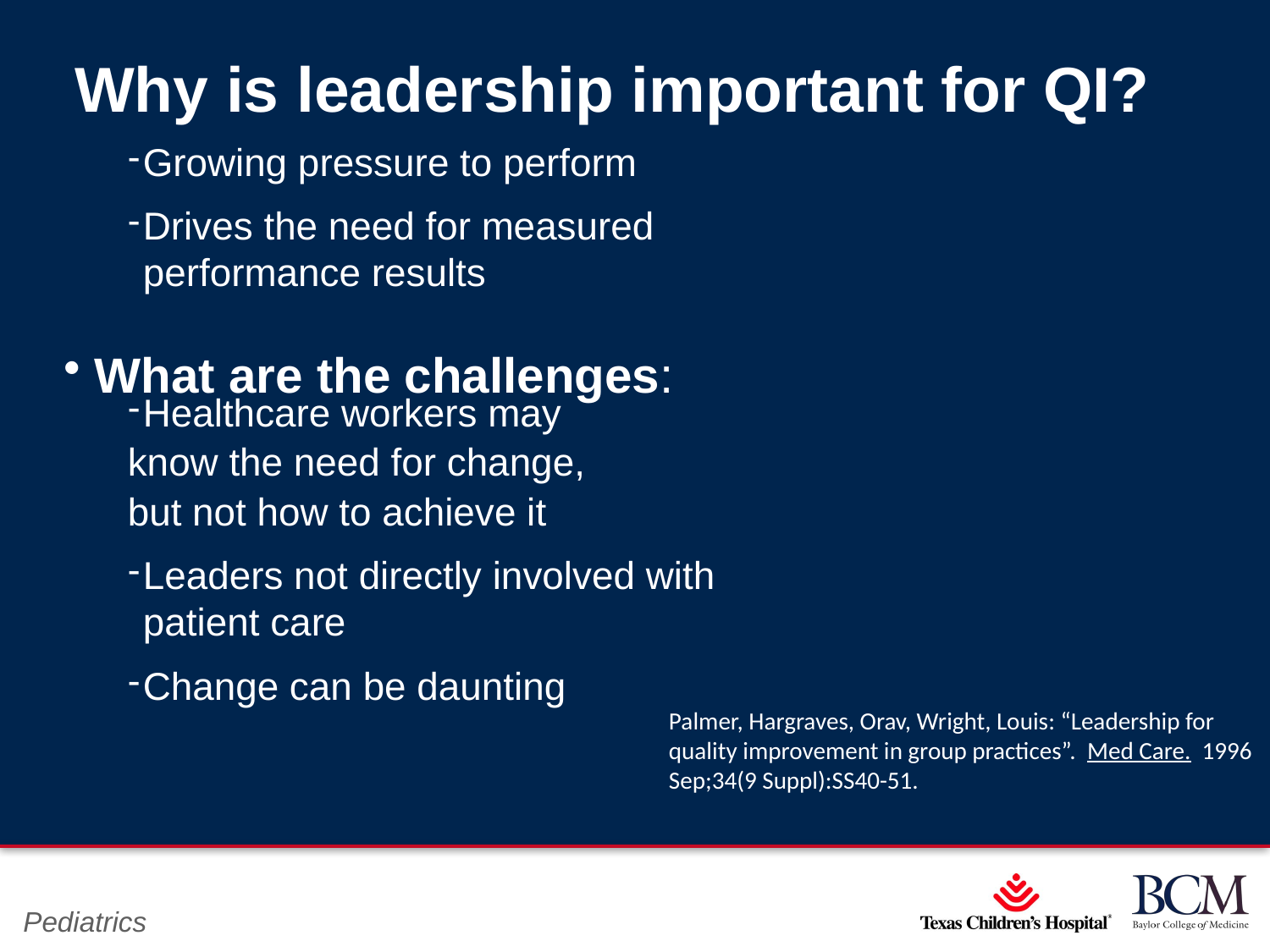

# Why is leadership important for QI?
Growing pressure to perform
Drives the need for measured performance results
 What are the challenges:
Healthcare workers may
know the need for change,
but not how to achieve it
Leaders not directly involved with patient care
Change can be daunting
Palmer, Hargraves, Orav, Wright, Louis: “Leadership for quality improvement in group practices”. Med Care. 1996 Sep;34(9 Suppl):SS40-51.

## Slide 17
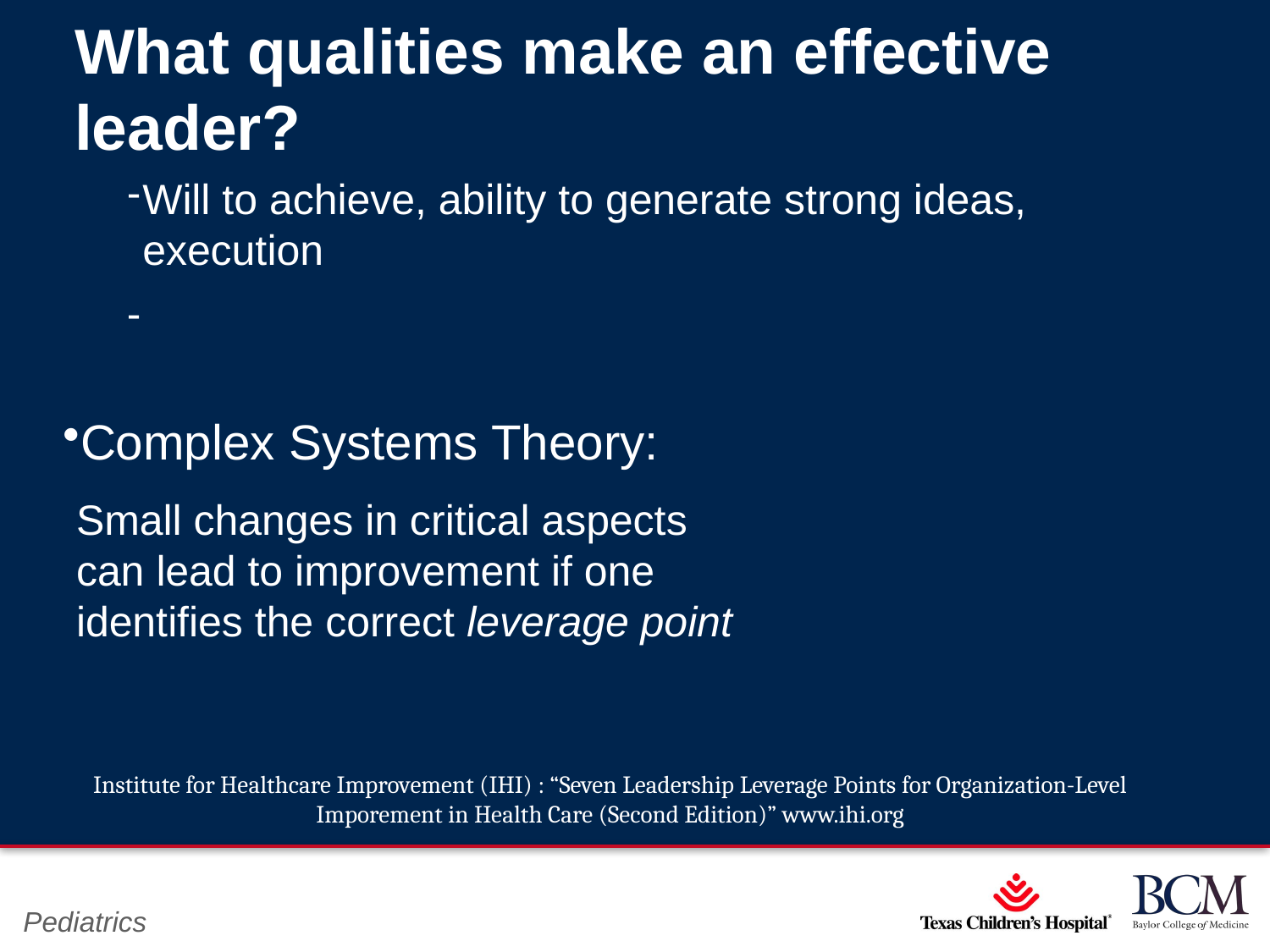

# What qualities make an effective leader?
Will to achieve, ability to generate strong ideas, execution
Adapt, spread, and sustain ideas
Complex Systems Theory:
Small changes in critical aspects
can lead to improvement if one
identifies the correct leverage point
Institute for Healthcare Improvement (IHI) : “Seven Leadership Leverage Points for Organization-Level
Imporement in Health Care (Second Edition)” www.ihi.org

## Slide 18
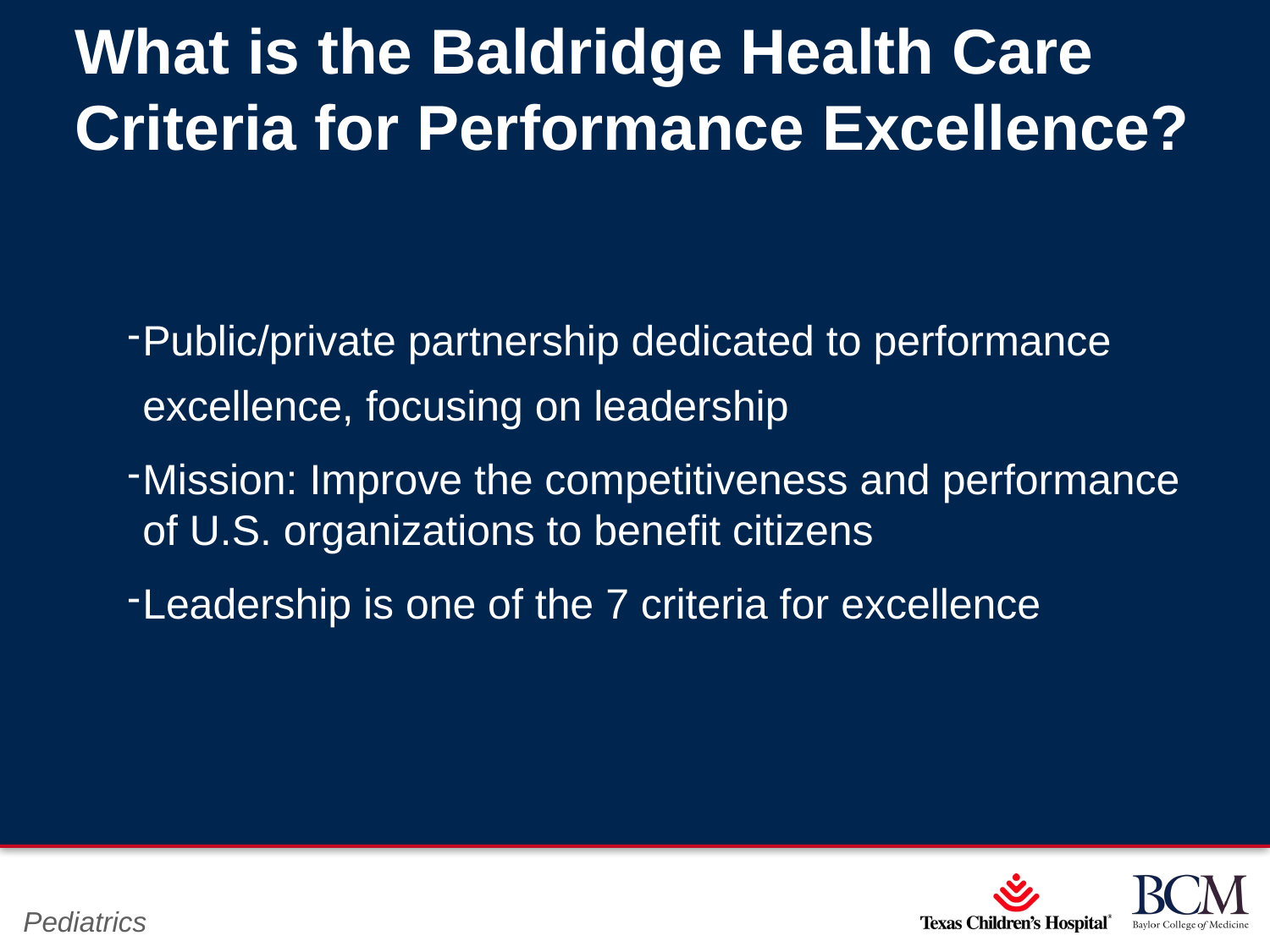

# What is the Baldridge Health Care Criteria for Performance Excellence?
Public/private partnership dedicated to performance excellence, focusing on leadership
Mission: Improve the competitiveness and performance of U.S. organizations to benefit citizens
Leadership is one of the 7 criteria for excellence

## Slide 19
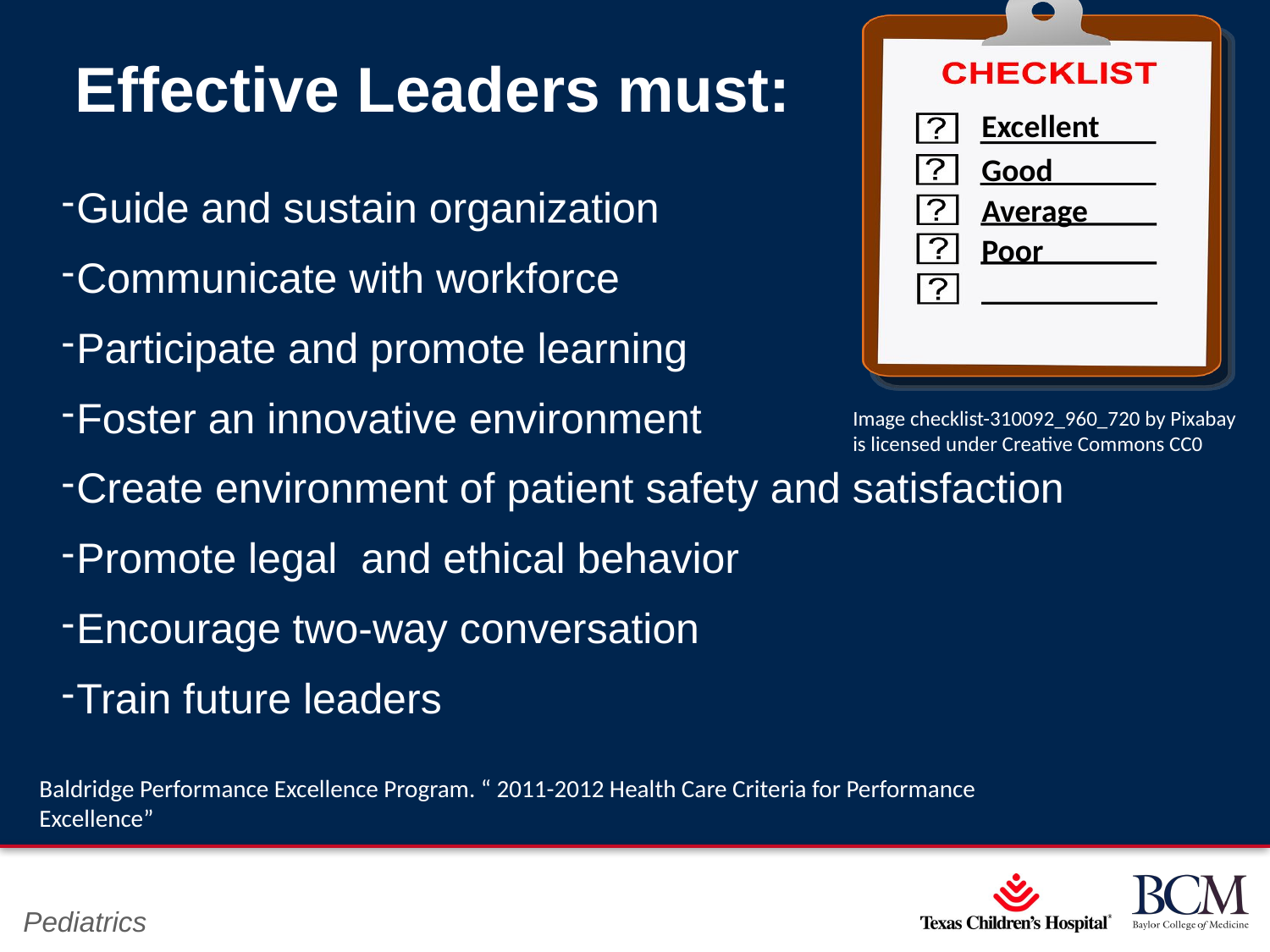

# Effective Leaders must:
Excellent
Good
Guide and sustain organization
Communicate with workforce
Participate and promote learning
Foster an innovative environment
Create environment of patient safety and satisfaction
Promote legal and ethical behavior
Encourage two-way conversation
Train future leaders
Average
Poor
Image checklist-310092_960_720 by Pixabay
is licensed under Creative Commons CC0
Baldridge Performance Excellence Program. “ 2011-2012 Health Care Criteria for Performance Excellence”

## Slide 20
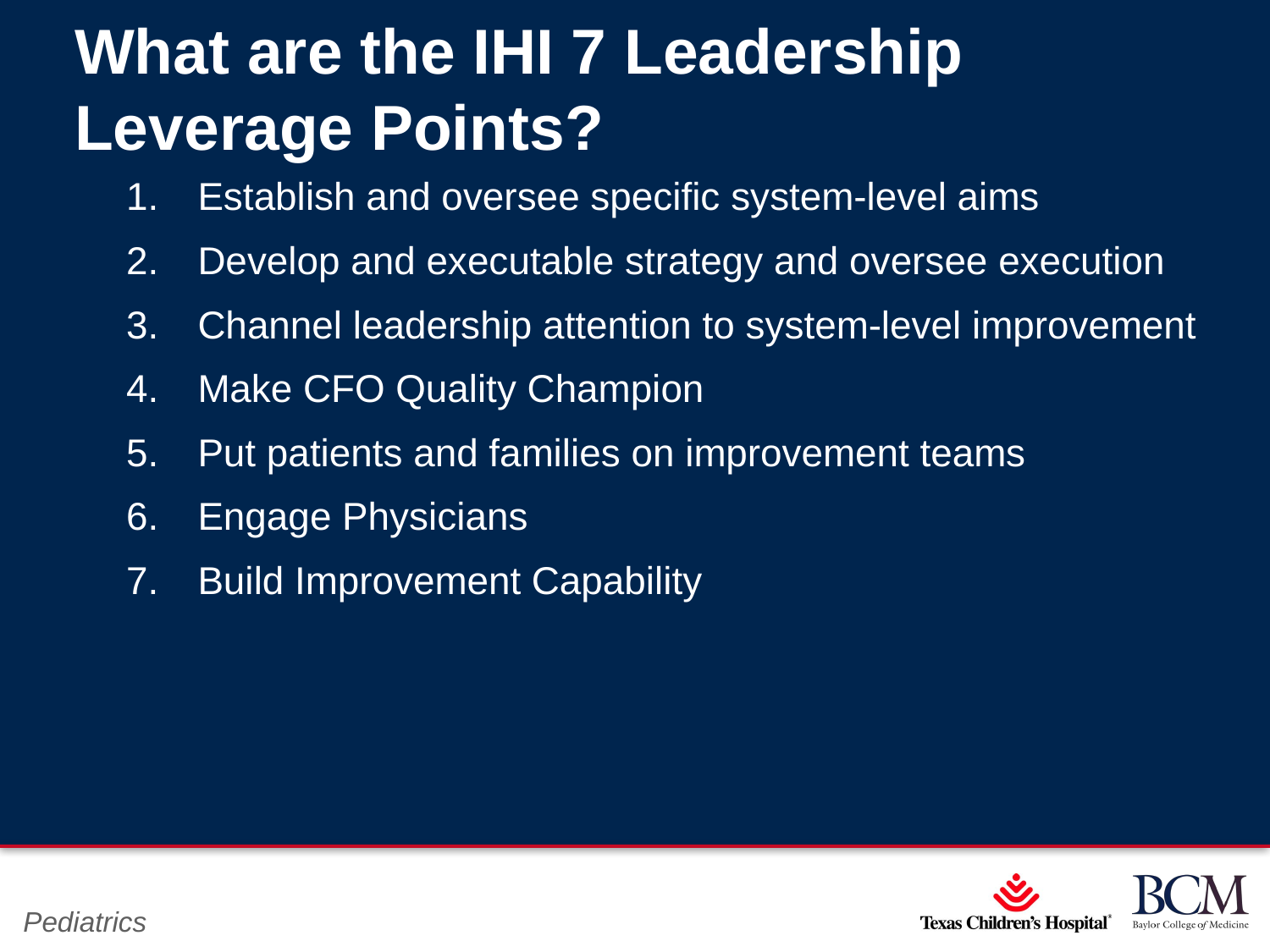

# What are the IHI 7 Leadership Leverage Points?
Establish and oversee specific system-level aims
Develop and executable strategy and oversee execution
Channel leadership attention to system-level improvement
Make CFO Quality Champion
Put patients and families on improvement teams
Engage Physicians
Build Improvement Capability

## Slide 21
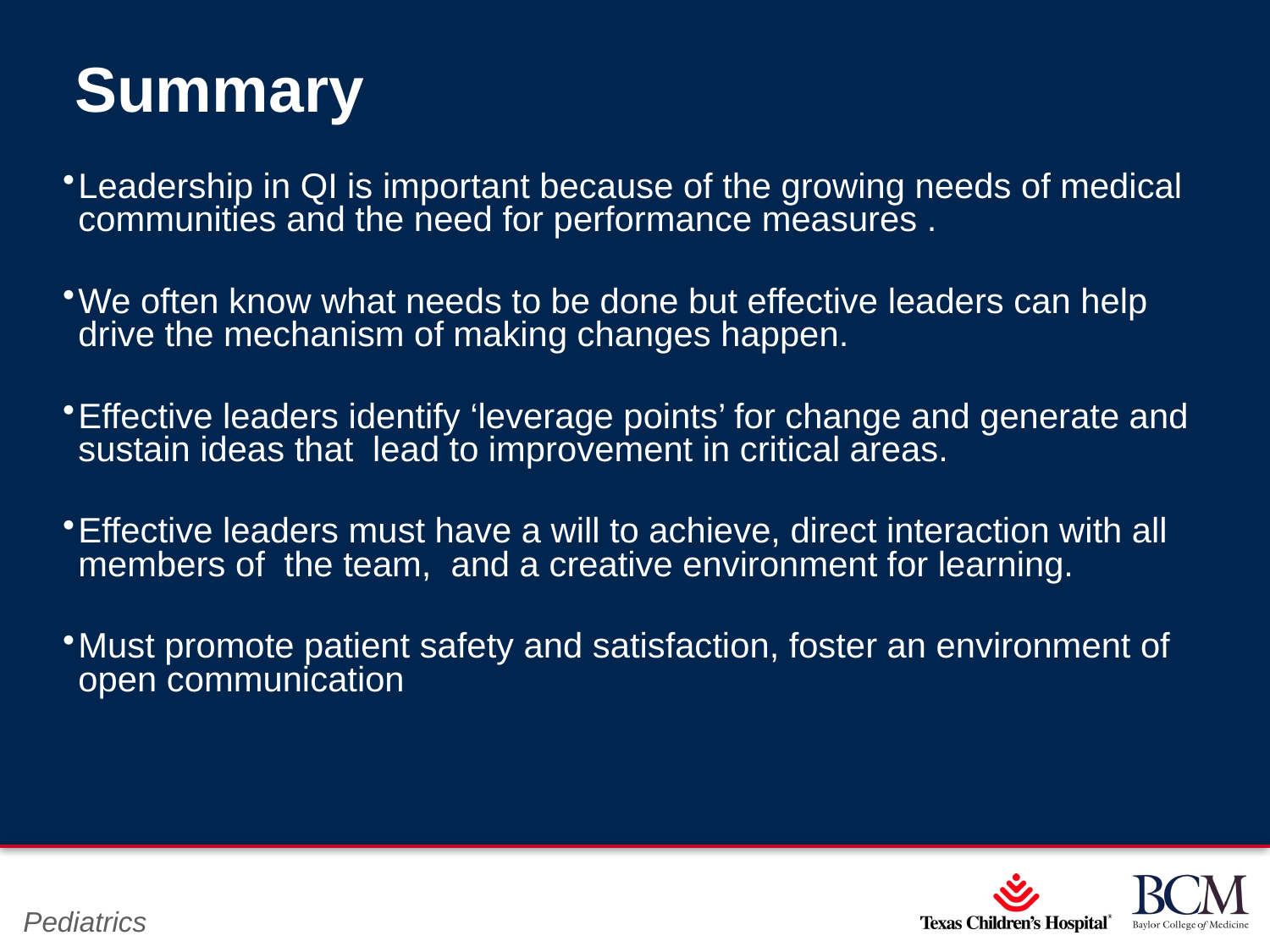

# Summary
Leadership in QI is important because of the growing needs of medical communities and the need for performance measures .
We often know what needs to be done but effective leaders can help drive the mechanism of making changes happen.
Effective leaders identify ‘leverage points’ for change and generate and sustain ideas that lead to improvement in critical areas.
Effective leaders must have a will to achieve, direct interaction with all members of the team, and a creative environment for learning.
Must promote patient safety and satisfaction, foster an environment of open communication

## Slide 22
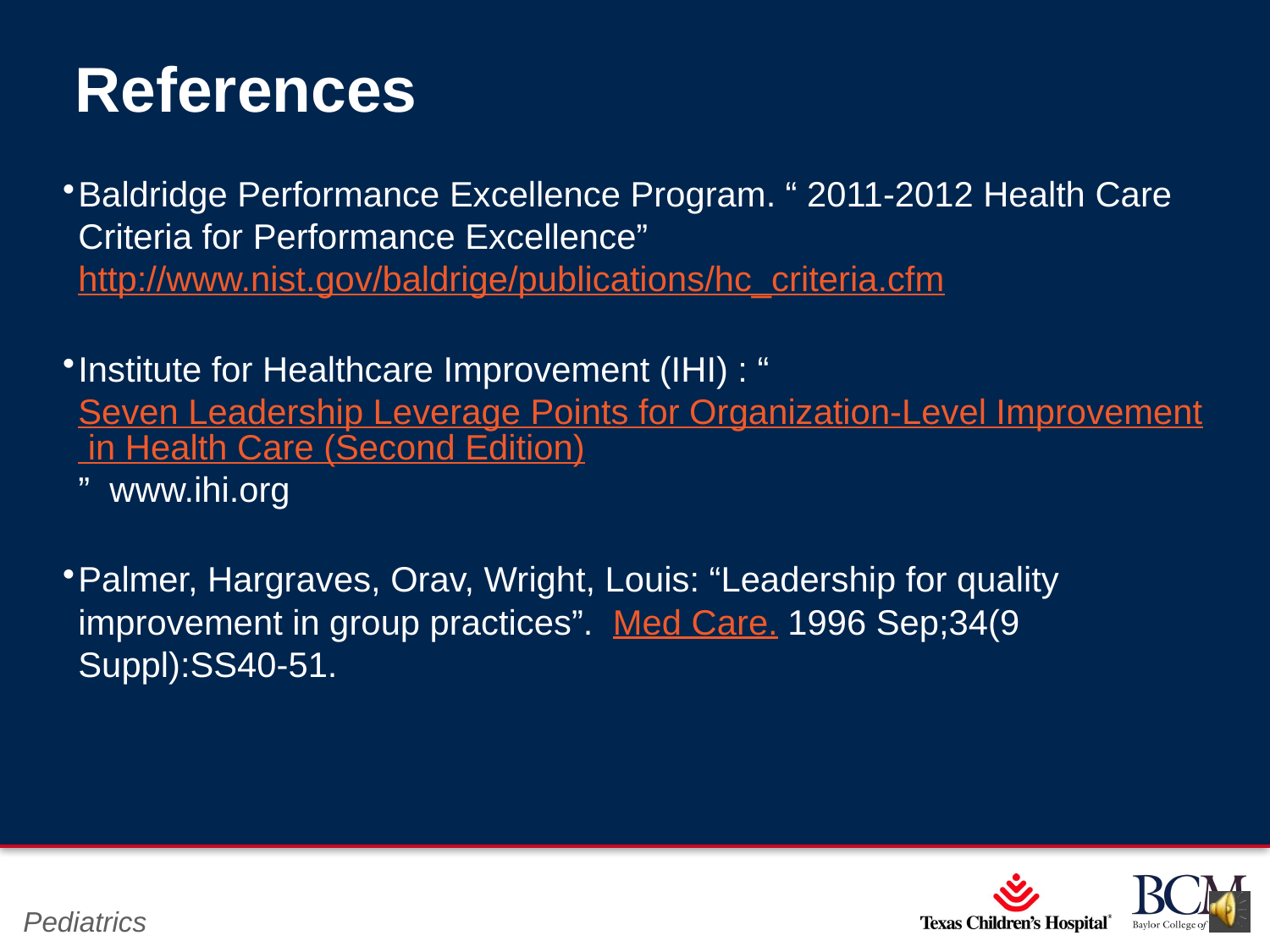

# References
Baldridge Performance Excellence Program. “ 2011-2012 Health Care Criteria for Performance Excellence” http://www.nist.gov/baldrige/publications/hc_criteria.cfm
Institute for Healthcare Improvement (IHI) : “Seven Leadership Leverage Points for Organization-Level Improvement in Health Care (Second Edition)” www.ihi.org
Palmer, Hargraves, Orav, Wright, Louis: “Leadership for quality improvement in group practices”. Med Care. 1996 Sep;34(9 Suppl):SS40-51.

## Slide 23
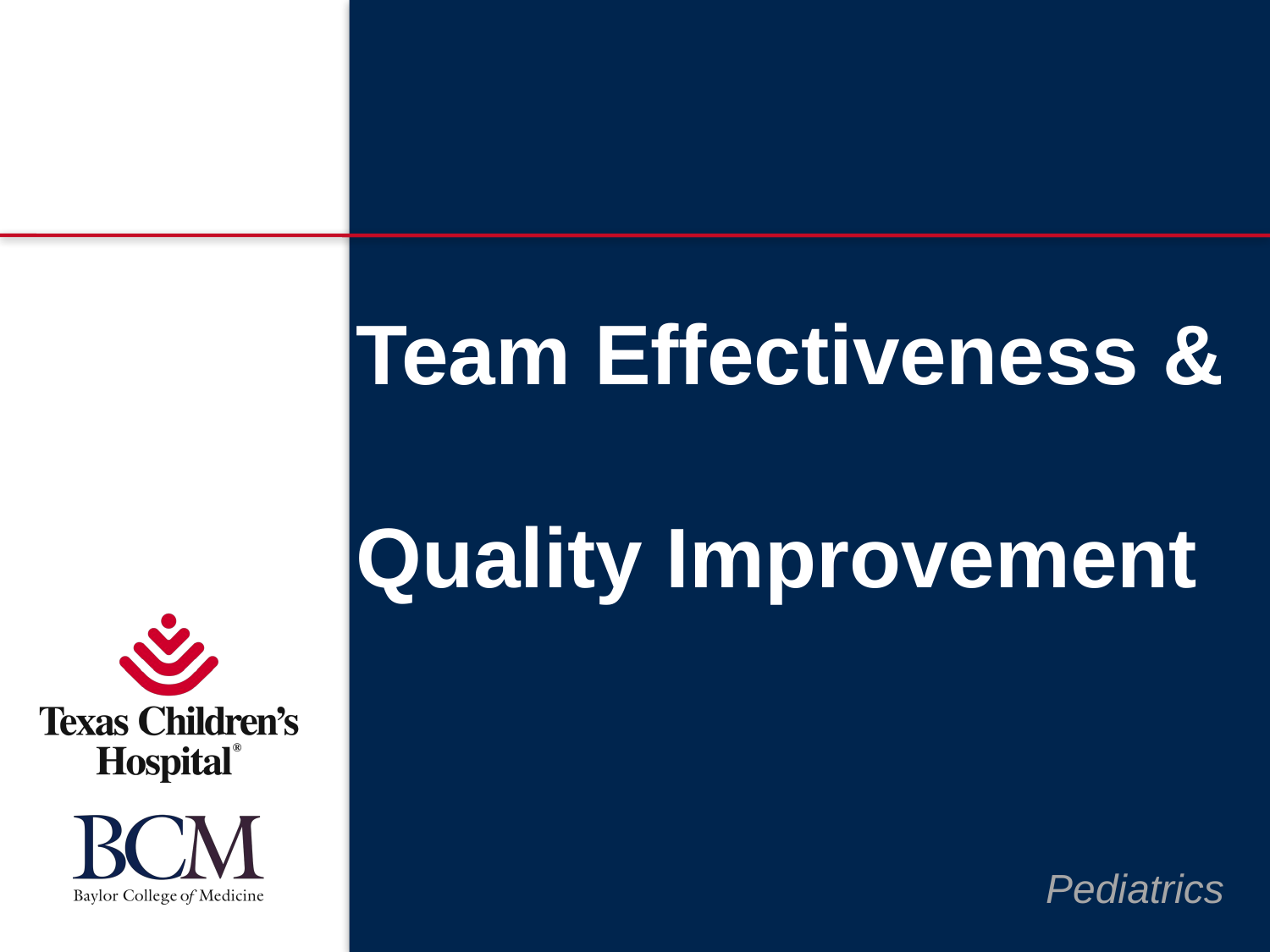

# Team Effectiveness & Quality Improvement

## Slide 24
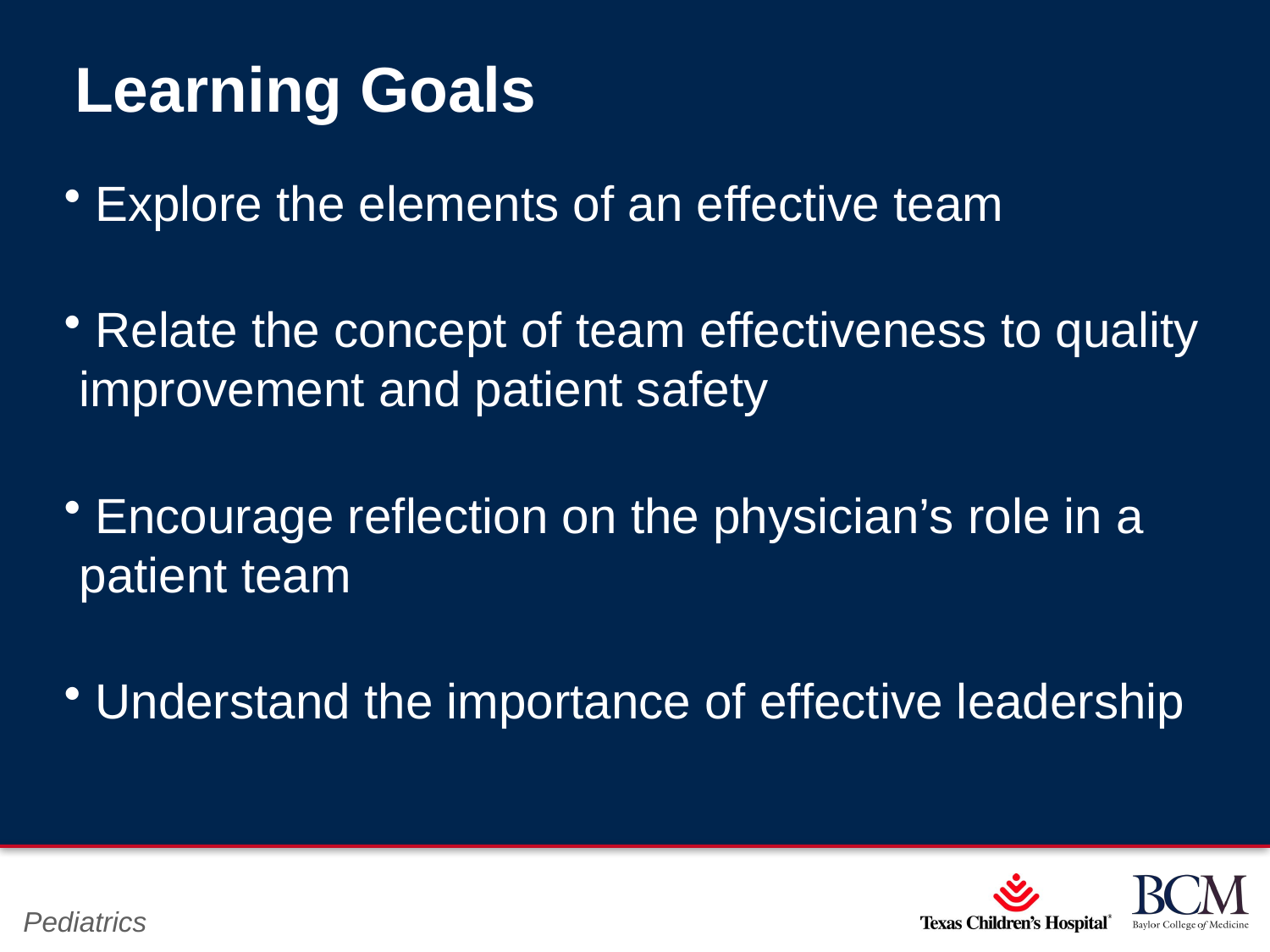

# Learning Goals
 Explore the elements of an effective team
 Relate the concept of team effectiveness to quality improvement and patient safety
 Encourage reflection on the physician’s role in a patient team
 Understand the importance of effective leadership

## Slide 25
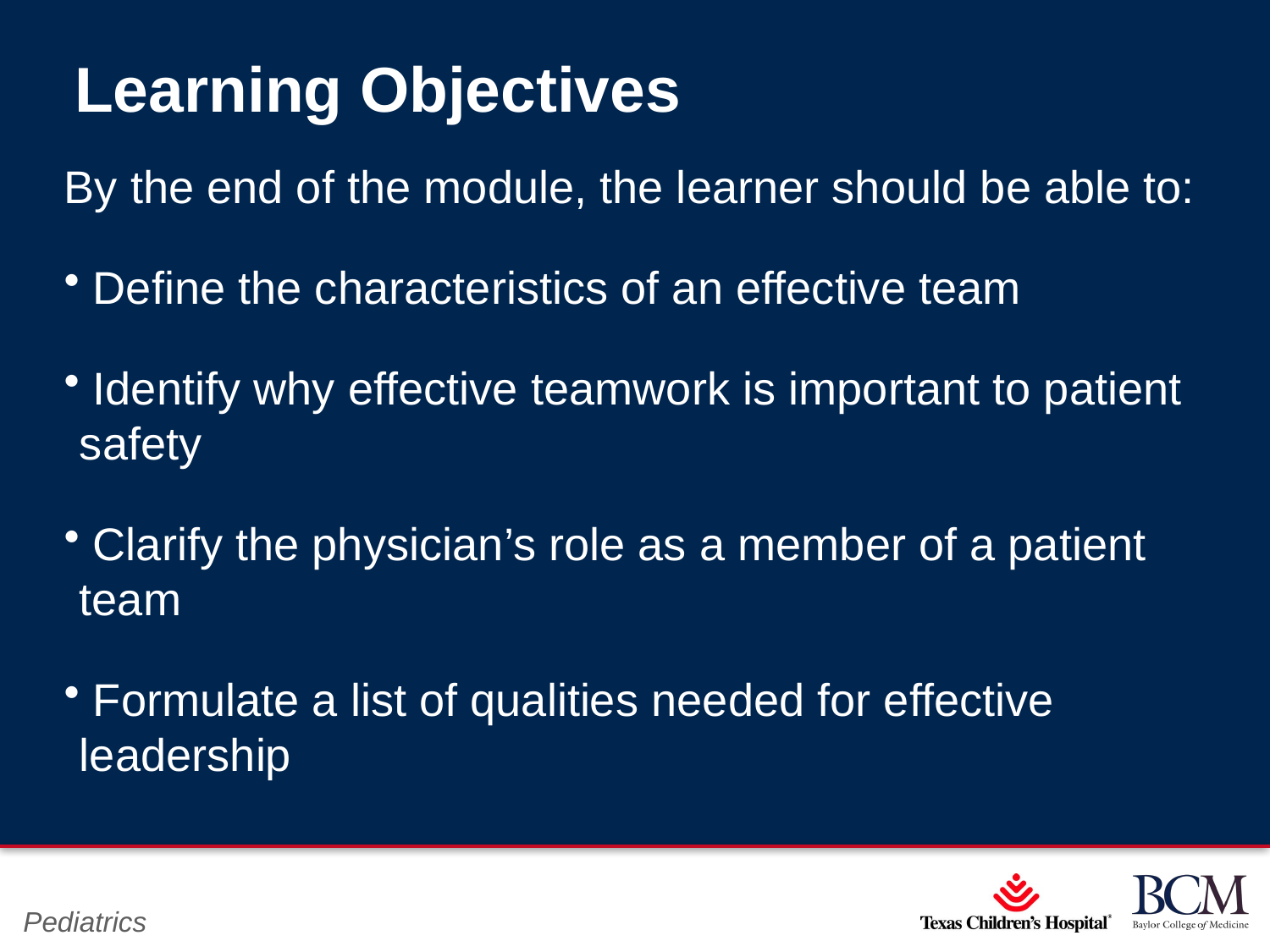

# Learning Objectives
By the end of the module, the learner should be able to:
 Define the characteristics of an effective team
 Identify why effective teamwork is important to patient safety
 Clarify the physician’s role as a member of a patient team
 Formulate a list of qualities needed for effective leadership

## Slide 26
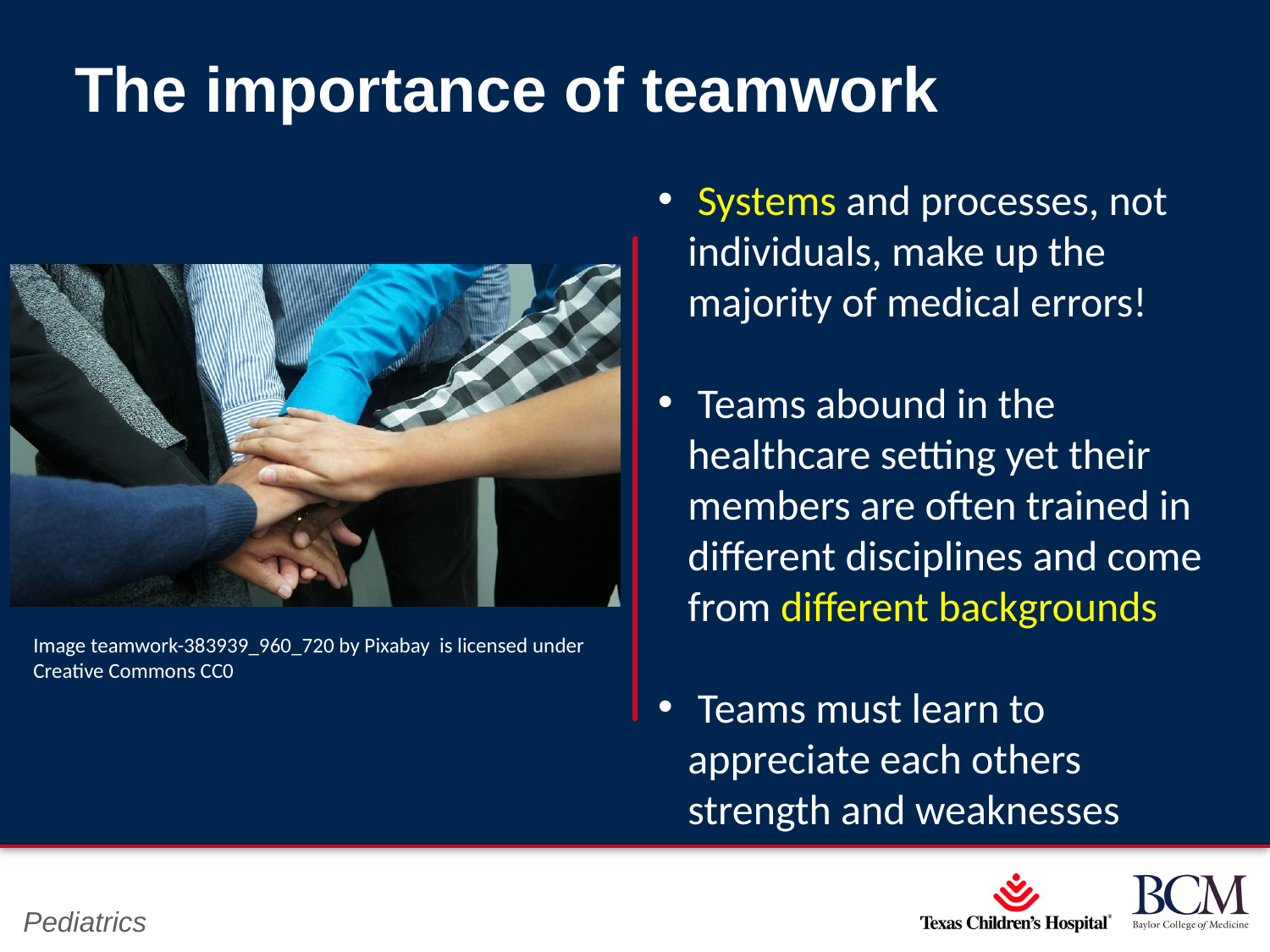

# The importance of teamwork
 Systems and processes, not individuals, make up the majority of medical errors!
 Teams abound in the healthcare setting yet their members are often trained in different disciplines and come from different backgrounds
 Teams must learn to appreciate each others strength and weaknesses
Image teamwork-383939_960_720 by Pixabay is licensed under Creative Commons CC0

## Slide 27
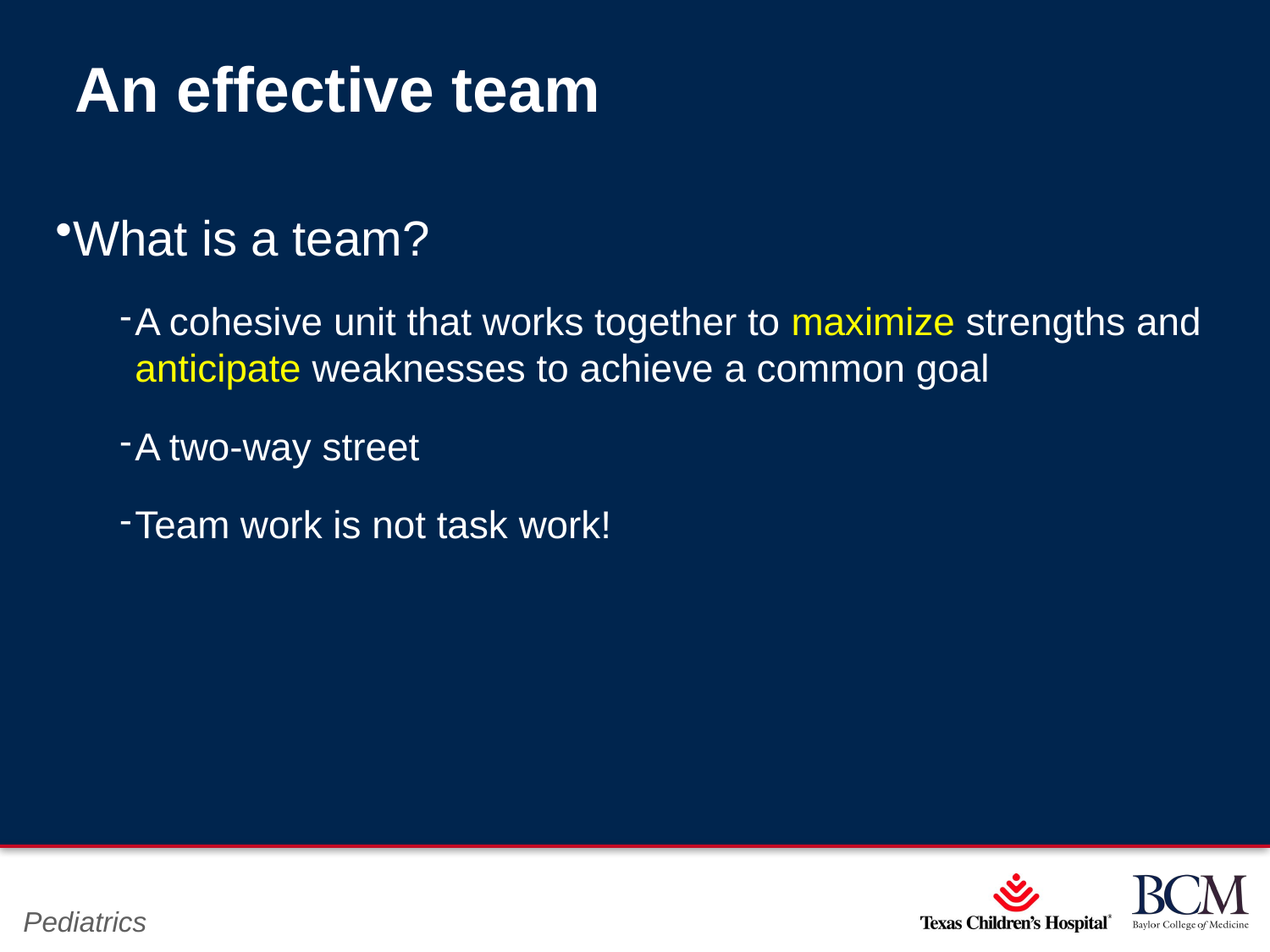

# An effective team
What is a team?
A cohesive unit that works together to maximize strengths and anticipate weaknesses to achieve a common goal
A two-way street
Team work is not task work!

## Slide 28
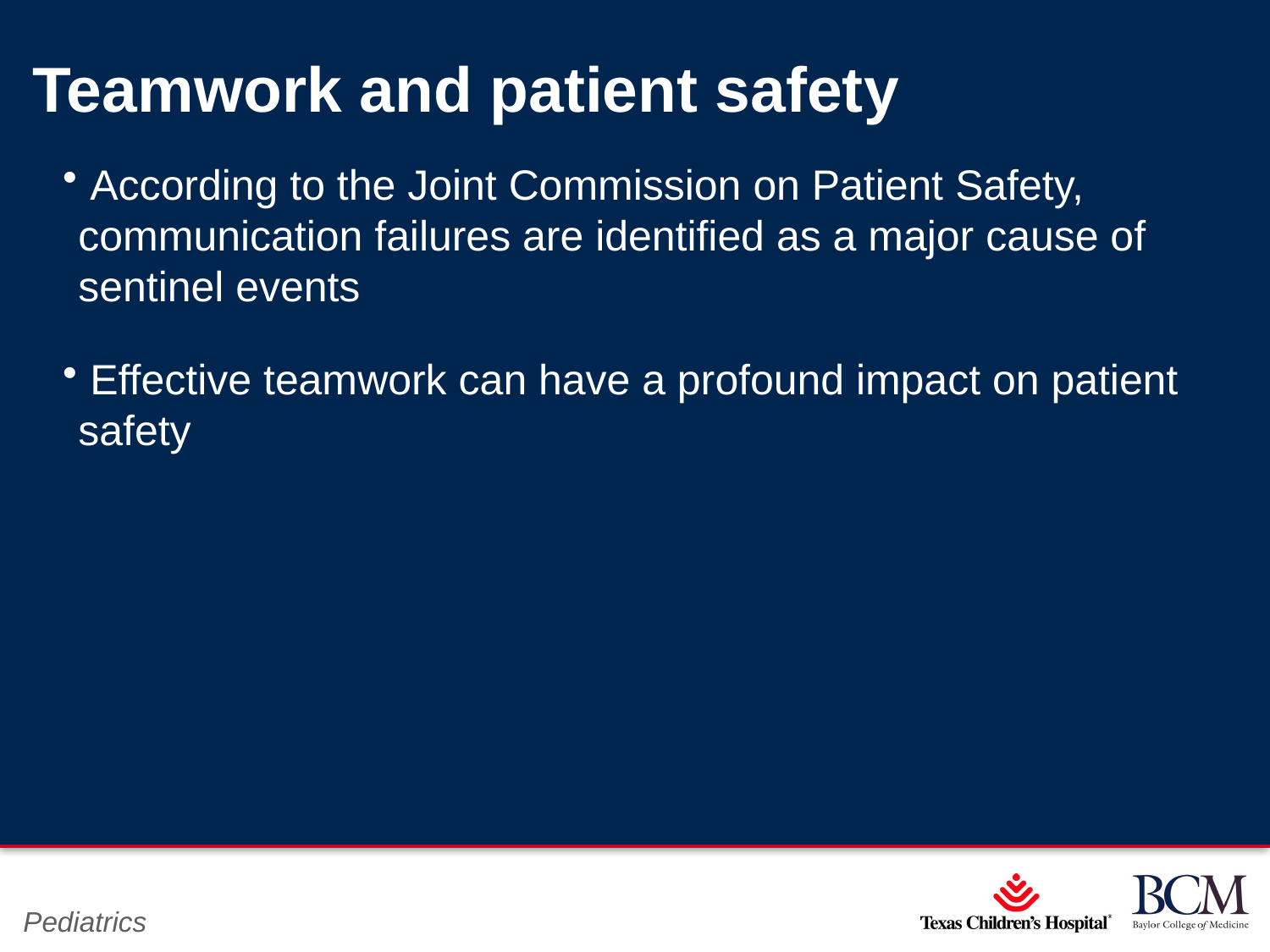

# Teamwork and patient safety
 According to the Joint Commission on Patient Safety, communication failures are identified as a major cause of sentinel events
 Effective teamwork can have a profound impact on patient safety

## Slide 29
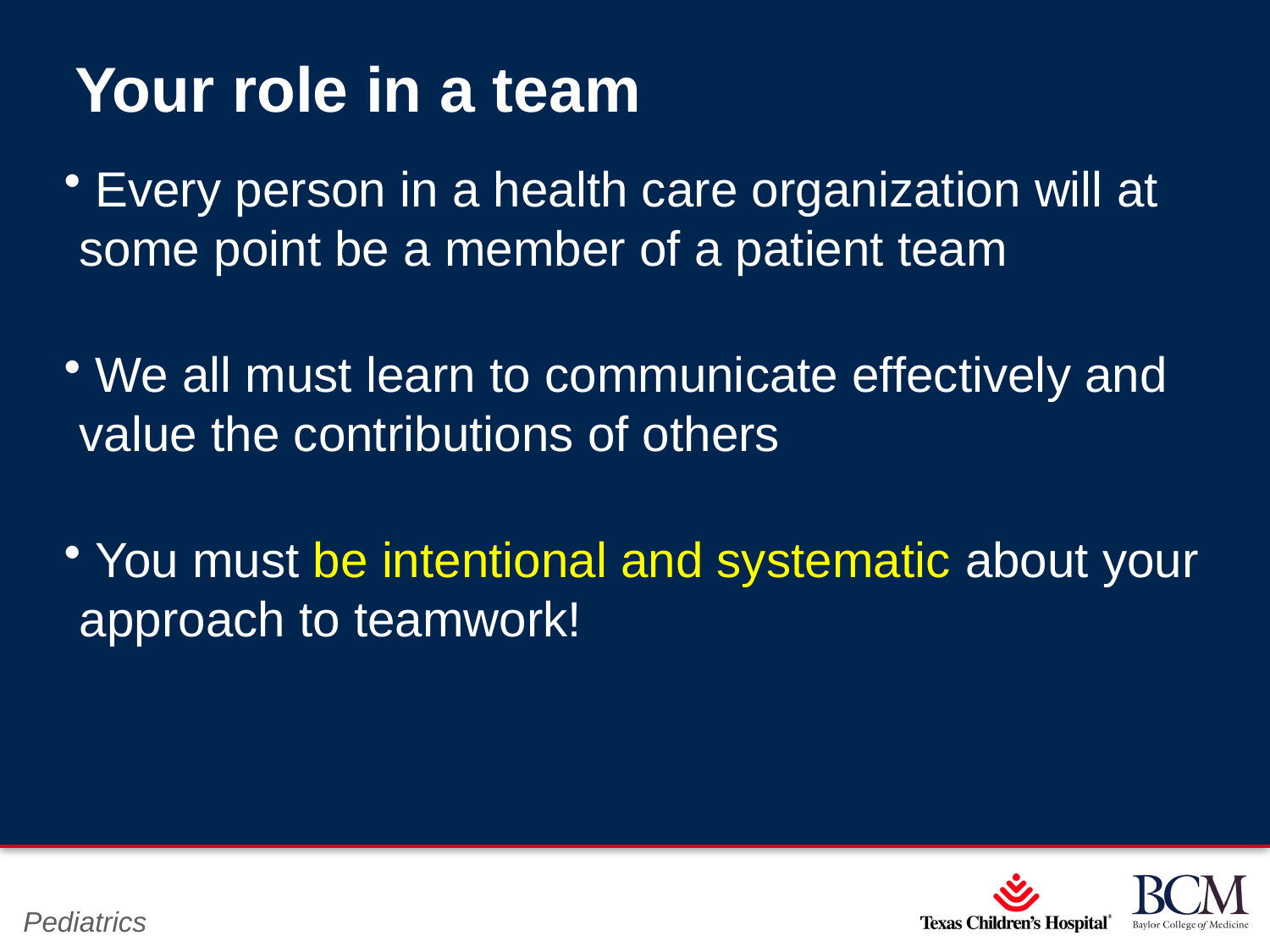

# Your role in a team
 Every person in a health care organization will at some point be a member of a patient team
 We all must learn to communicate effectively and value the contributions of others
 You must be intentional and systematic about your approach to teamwork!

## Slide 30
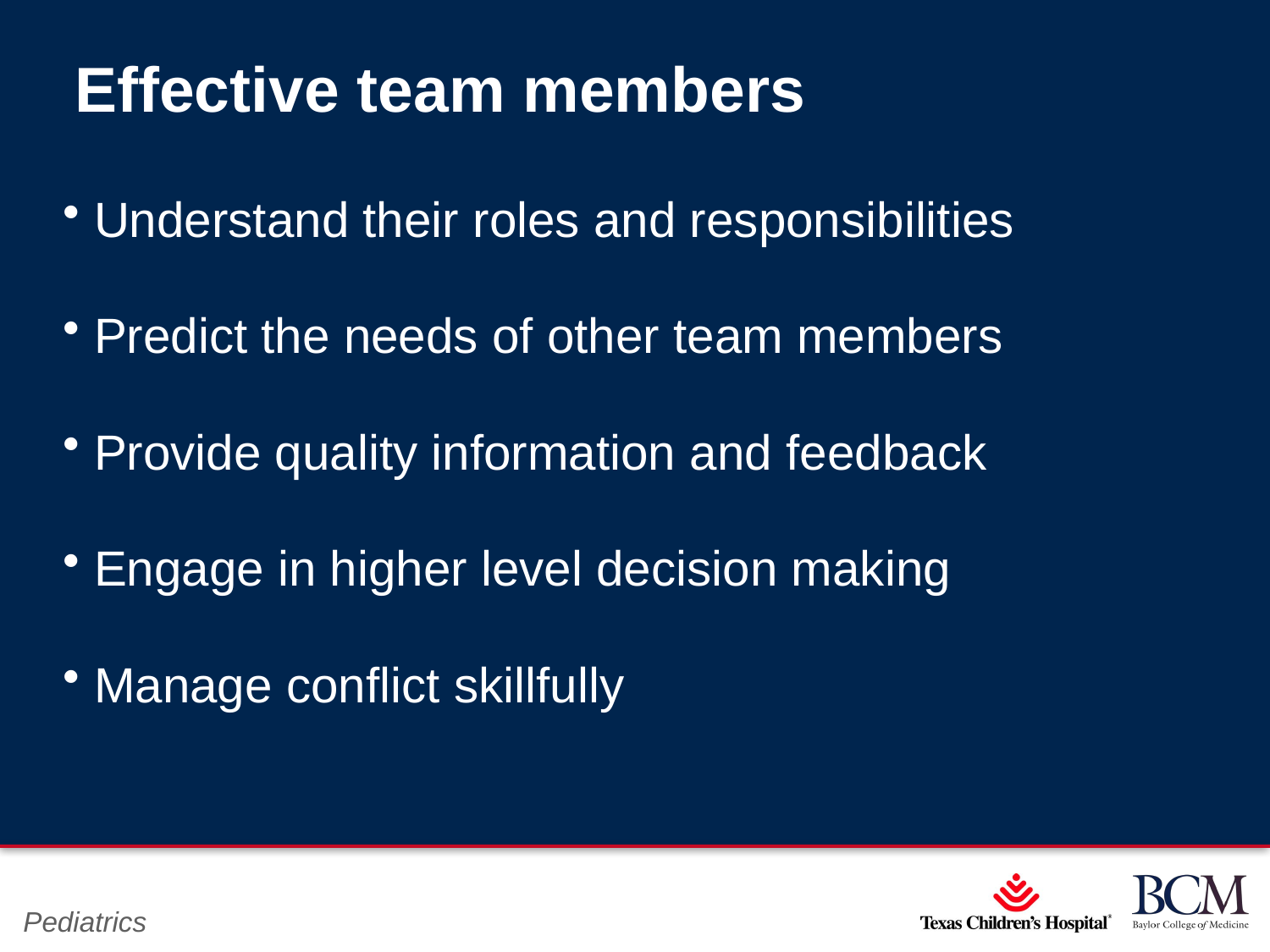

# Effective team members
 Understand their roles and responsibilities
 Predict the needs of other team members
 Provide quality information and feedback
 Engage in higher level decision making
 Manage conflict skillfully

## Slide 31
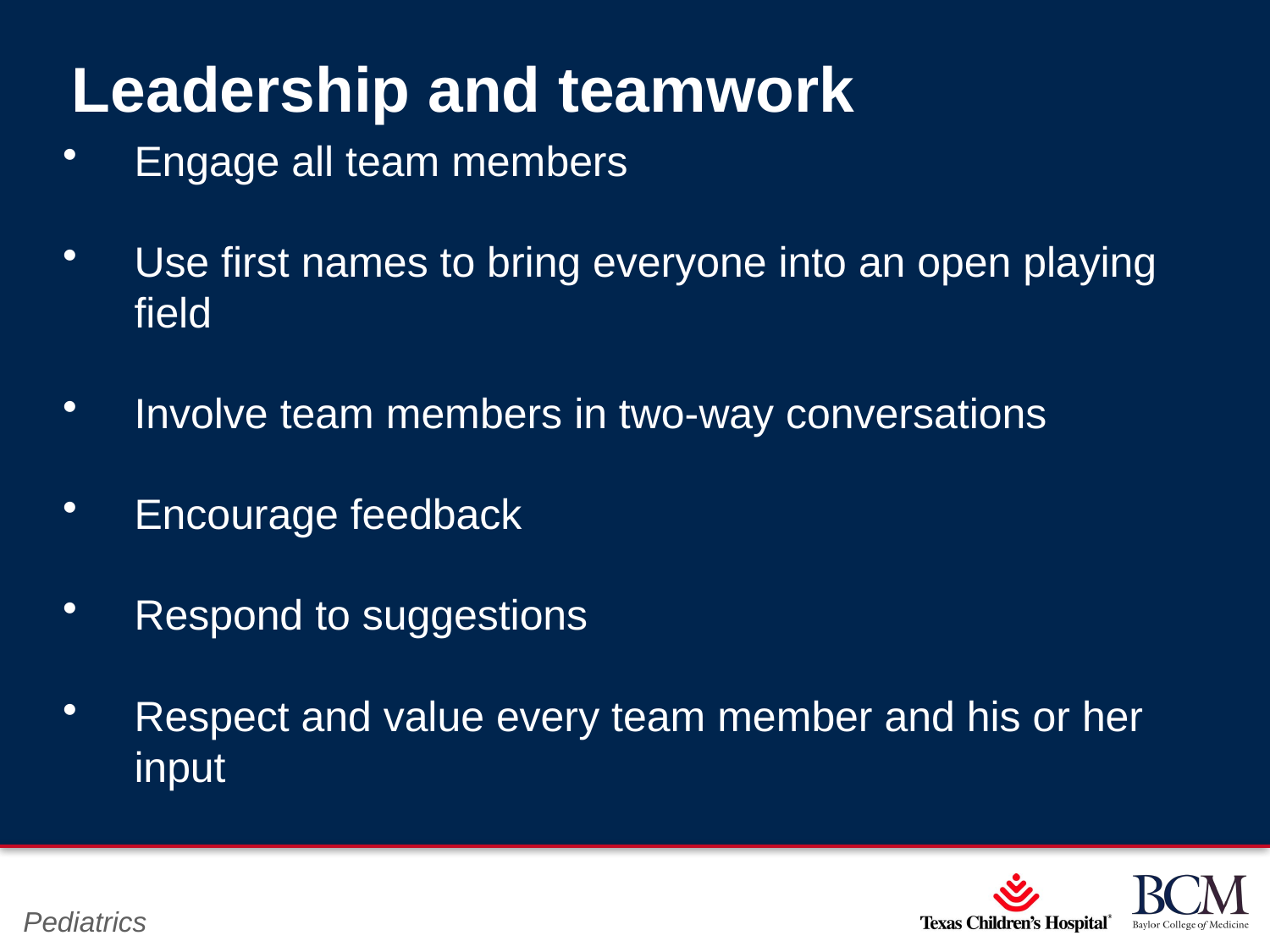

# Leadership and teamwork
Engage all team members
Use first names to bring everyone into an open playing field
Involve team members in two-way conversations
Encourage feedback
Respond to suggestions
Respect and value every team member and his or her input

## Slide 32
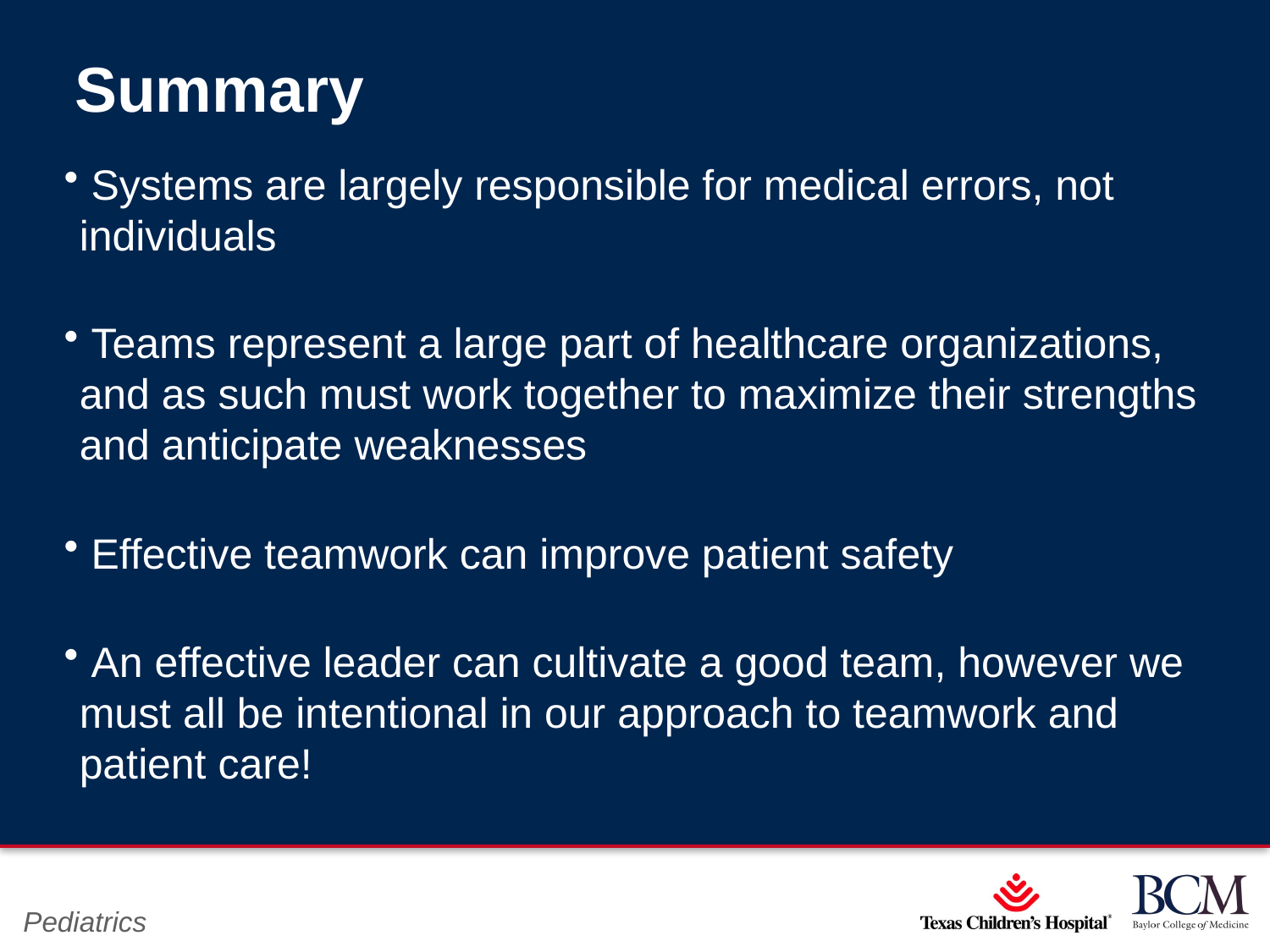

# Summary
 Systems are largely responsible for medical errors, not individuals
 Teams represent a large part of healthcare organizations, and as such must work together to maximize their strengths and anticipate weaknesses
 Effective teamwork can improve patient safety
 An effective leader can cultivate a good team, however we must all be intentional in our approach to teamwork and patient care!

## Slide 33
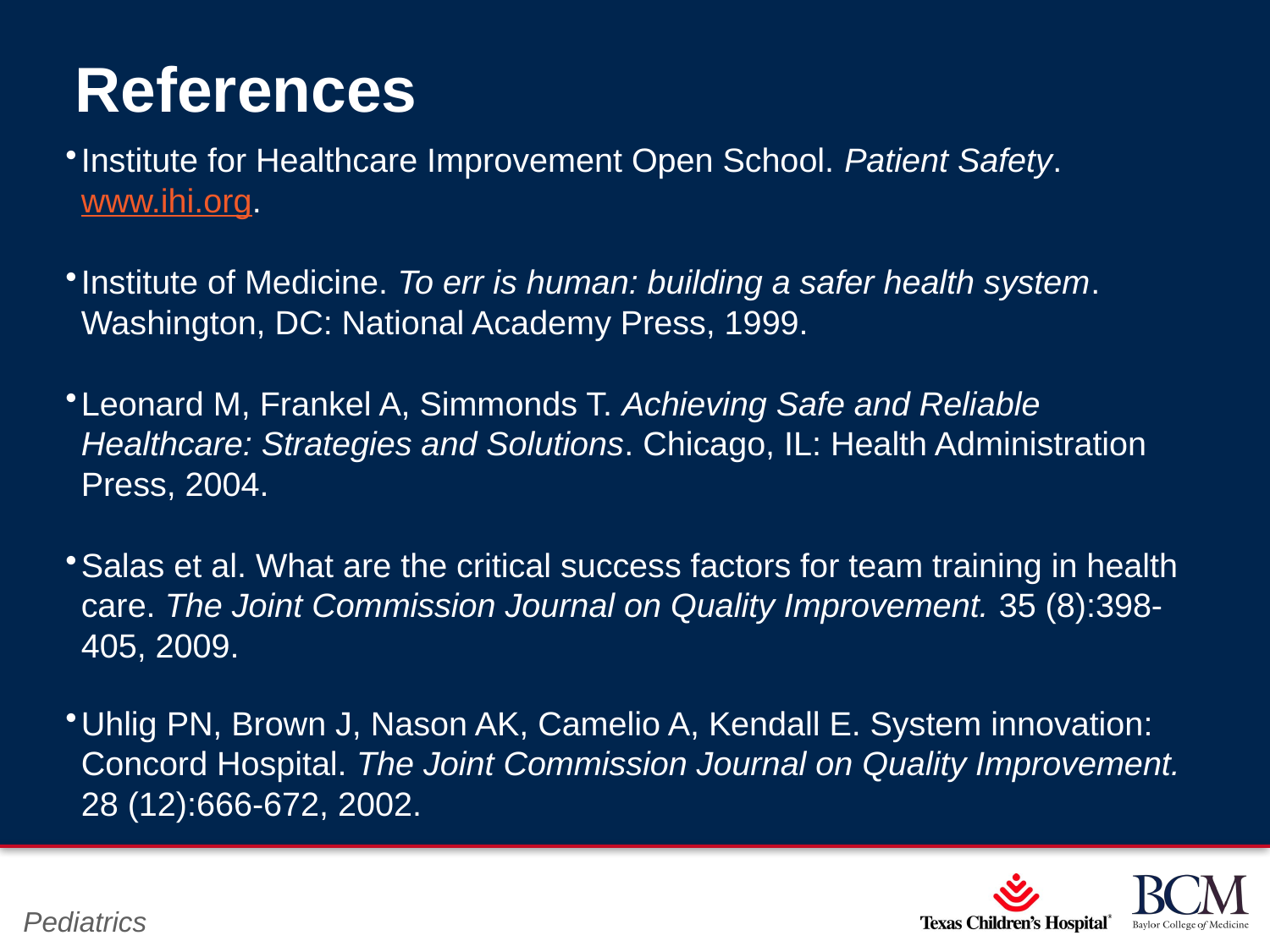

# References
Institute for Healthcare Improvement Open School. Patient Safety. www.ihi.org.
Institute of Medicine. To err is human: building a safer health system. Washington, DC: National Academy Press, 1999.
Leonard M, Frankel A, Simmonds T. Achieving Safe and Reliable Healthcare: Strategies and Solutions. Chicago, IL: Health Administration Press, 2004.
Salas et al. What are the critical success factors for team training in health care. The Joint Commission Journal on Quality Improvement. 35 (8):398-405, 2009.
Uhlig PN, Brown J, Nason AK, Camelio A, Kendall E. System innovation: Concord Hospital. The Joint Commission Journal on Quality Improvement. 28 (12):666-672, 2002.
