# Supplementary material for: Engaging Learners Through Modules in Quality Improvement and Patient Safety
Source: MedEdPORTAL. 2016 Oct 13;12:10482. doi: 10.15766/mep_2374-8265.10482 (PMC6440404; doi:10.15766/mep_2374-8265.10482)
Supplement: Supplementary file 1 — A. Instructor's Guide.docx B. PowerPoint Talking Points.docx C. Knowledge Survey.docx D. Attitude Survey Questions.docx E. Fundamentals of QI.pptx F. Fundamentals of Patient Safety.ppt G. Evidence-Based Practice and QI Improvement Research.pptx H. QI and PS Potpourri.pptx [file mep-12-10482-s001.zip › _Educational Summary Report_10482.pdf]

MedEdPORTAL®
The Journal of Teaching and Learning Resources

Original Publication OPEN ACCESS

Engaging Learners Through Modules in Quality Improvement and Patient Safety

Diana Stewart, MD, MBA*, Cara Lye, MD, Michelle Lopez, MD, MPH, Brent Mothner, MD, Elizabeth Camp, PhD, Joyee Vachani, MD, MEd
*Corresponding author: anukwuem@bcm.edu

Abstract

Introduction: Residents are on the front lines of medical care in academic institutions. Their daily interactions are crucial to the quality of care received by patients in these settings, and thus, knowledge of patient safety and quality improvement is essential. The Accreditation Council for Graduate Medical Education requires all residents to participate in quality improvement and patient safety programs as part of their residency training. To meet this need, we developed a curriculum in patient safety and quality improvement for pediatric residents. **Methods:** This curriculum describes four short modules focused on quality improvement, patient safety, evidence-based practice, and other quality improvement–related topics. These modules can be given during one rotation, throughout residency, or partnered with a practical application, such as a project. . A 17-question quality improvement and patient safety knowledge test was developed after an extensive literature review to reflect module goals and objectives. A validated, 12-question attitudes survey was administered before and after the modules. **Results:** Of the 57 eligible residents, 42 completed the knowledge pretest, and 20 completed the posttest. Mean posttest results (M = 91.00 [± 9.12]) were considerably higher than mean pretest scores (M = 75.24 [± 11.74]) when utilizing the independent t test ($p < .001$). Of the 57 eligible residents, 11 completed the attitude presurvey, and 13 completed the attitude postsurvey. Median responses from the survey mostly fell within the 2-3 range of slightly to moderately comfortable. Significant differences showing improvement between presurvey and postsurvey time frames were found in identifying and comparing best practices ($p = .02$), using the PDSA model ($p = .002$), and identifying how data are linked ($p = .001$). **Discussion:** Knowledge and perception surveys suggest that resident knowledge and attitudes statistically improved, and faculty and residents participated in even more quality improvement initiatives after completing the curriculum.

Keywords

Patient Safety, Quality Improvement

Educational Objectives

After completing this curriculum, residents will be able to:

1. Define the model for improvement and utilize the format in future work.
2. Explain the components of an aim statement and write example statements.
3. Compare adverse and sentinel events and contrast them using specific examples.
4. Describe how evidence-based practice relates to quality improvement and list potential interventions for change.
5. Systematically analyze their own current practice using quality improvement methods and identify areas of potential modification.
6. Review how effective leadership and multidisciplinary teams promote safe care and apply it to their health care system.
7. Apply learned concepts to future quality improvement initiatives at their institution.

Citation: Stewart D, Lye C, Lopez M, Mothner B, Camp E, Vachani J. Engaging learners through modules in quality improvement and patient safety. *MedEdPORTAL*. 2016;12:10482. https://doi.org/10.15766/mep_2374-8265.10482

Copyright: © 2016 Stewart et al. This is an open-access article distributed under the terms of the Creative Commons Attribution license.

Appendices

A. Instructor's Guide.docx
B. PowerPoint Talking Points .docx
C. Knowledge Survey.docx
D. Attitude Survey Questions .docx
E. Fundamentals of QI.pptx
F. Fundamentals of Patient Safety.ppt
G. Evidence-Based Practice and QI Improvement Research.pptx
H. QI and PS Potpourri.pptx

All appendices are peer reviewed as integral parts of the Original Publication.

Introduction

Residents are essential to patient care in academic institutions. Their daily interactions are important to the quality of care received and patient outcomes in these settings, and therefore, knowledge of patient safety and quality improvement is essential. The Accreditation Council for Graduate Medical Education (ACGME) requires all residents to participate in quality improvement and patient safety programs as part of their residency training.[1-3] The ACGME Clinical Learning Environment Review (CLER) provides programs with periodic feedback regarding trainee education in six areas, two of these being patient safety and quality improvement.[3] Recently, the Association of Pediatric Program Directors developed a survey of quality improvement curricula for program directors and found that they were rarely longitudinal (12%) and that the major barriers to teaching were time (66%), funding (39%), and lack of quality improvement expertise (33%).[4] To meet this need, we developed a curriculum in patient safety and quality improvement for pediatric residents. This curriculum addresses all of these barriers, allowing faculty with minimal quality improvement training, limited time, and minimal resources to teach quality improvement.

In preparation, we reviewed similar MedEdPORTAL publications available on this topic. Two curricula focus on the model for improvement and incorporate a simultaneous quality improvement project.[5,6] Tapper, Sullivan, and Tess outline a curriculum to teach quality improvement on wards using a longitudinal iterative approach focusing on a quality improvement project in an ambulatory rotation while including evaluations that use CLER-specific language.[7] Tad-y, Price, Cumbler, Levin, Wald, and Glasheen provide a handbook of resources that can be used to implement quality improvement curricula.[8] Most of these focus on an experiential component. Our curriculum describes short modules that can be given as a supplement to a project-focused curriculum or can stand alone. While previous curricula have focused on quality improvement only, these modules are not limited to quality improvement but also teach elements of patient safety and evidence-based practice, realizing that all three topics operate along a continuum. In order to keep patients safe, the care they are provided must be continuously assessed and improved, while incorporating new evidence.

The target learners for this curriculum are pediatric residents of all postgraduate levels; however, this curriculum can be easily adapted to any residency training program. Additionally, other learner groups would also benefit from this tool, including medical students and interprofessional trainees (e.g., nurses, physician assistants, etc.). We currently give these lectures to our first-year pediatric residents. Using core features of adult education theory and quality improvement and patient safety methodology, we developed modules that provide residents with fundamentals of quality improvement and patient safety to increase their ability to apply learned principles to practice.

Methods

Learners receive education via PowerPoint presentations, small-group discussions with case discussions, and feedback and reflection with the course facilitators as the materials are presented.

This curriculum consists of four modules, each 30 minutes or less. These modules can be given during one rotation, throughout residency, or partnered with a practical application such as a project. These modules are currently given at our institution during an ambulatory rotation for first-year pediatric residents by faculty facilitators.

Appendix A includes an instructor's guide to aid in implementation. The talking points of the presentations are included in Appendix B. Appendices C and D include the knowledge and attitude surveys used for evaluation. Below is a detailed description of each module.

Module 1. Fundamentals of Quality Improvement (Appendix E)

The goal of this module is to provide a basic understanding of quality improvement. It reviews the Institute of Medicine's aims for improvement as well as the Institute for Health Care Improvement model for improvement. After completing this module, learners will be able to describe why quality improvement is important, recite the Institute of Medicine aims for improvement, define the model for improvement, generate an aim statement, and practice the plan, do, study, act (PDSA) cycle.

Module 2. Fundamentals of Patient Safety (Appendix F)
The goal of this module is to provide a basic overview of patient safety. This module explores how medical error is a leading cause of death in our country and discusses methods at our institution to reverse this alarming statistic. After completing this module, learners will be able to define adverse and sentinel events, list types of medical errors, and outline how to file an incident report involving a safety issue at their institution.

Module 3. Evidence-Based Practice and Quality Improvement Research (Appendix G)
The primary goal of this module is to define evidence-based practice, introduce basic concepts of evidence-based practice and quality improvement research, and discuss how both of these concepts can be applied to daily patient care. After completing this module, learners will be able to define evidence-based practice and apply it to quality improvement, identify and know how to access appraisal tools, and recognize different types of quality improvement studies.

Module 4. Quality Improvement and Patient Safety Potpourri (Appendix H)
This module includes three topics: quality improvement and health care policy, leadership and quality improvement, and team effectiveness and quality improvement. Many learners do not understand how quality improvement initiatives impact society and populations locally, nationally, and globally. However, health care policy can dictate and guide the aims and metrics of an institution. Trainees must also recognize that effective leadership and teams are essential to successful quality improvement initiatives, the promotion of a culture of safety, and decreasing medical errors. After completing this module, learners will be able to discuss how quality improvement impacts health care policy, examine qualities of an effective quality improvement leader and team, and recognize how effective leadership and multidisciplinary teams promote and enhance safe care.

The resources needed for this curriculum include the faculty and residents' time, a laptop or computer connected to a projector to run the PowerPoint, and learning space. Each module is about 30 minutes; faculty will need to allocate 1.5 hours to teach all four modules. Additional preparation time is needed prior to the module for faculty to familiarize themselves with the material. The preparation time will vary for each facilitator. Average preparation time at our institution was about an hour, which decreased with subsequent sessions. Residents are scheduled to receive the lectures during an outpatient ambulatory rotation and do not have any clinical responsibilities while they are receiving the didactics. A block of time will need to be allocated for learners to receive the modules.

Statistical Analysis
Unmatched pre- and posttest comparisons were made among the 44 residents enrolled in the study. Test score distributions were found to be skewed using the Shapiro-Wilk test for normality. Therefore, nonparametric testing was utilized to determine statistical significance in addition to parametric testing. Nonparametric testing (the Mann-Whitney *U* test) was also utilized in analyzing unmatched pre- and posttest self-efficacy survey results since the survey consisted of Likert-based responses, utilizing a 1-4 scale. Means, standard deviations, medians, and interquartile ranges are provided, with statistical significance defined as a *p* value less than .05. All statistics were computed using the Statistical Package for the Social Sciences (SPSS) Version 23 software (IBM Corp., Armonk, NY). Qualitative and quantitative assessments of faculty were not obtained.

Results

Kirkpatrick's model of training evaluation describes levels of learning from the most basic reaction up to learning, transfer, and results.[9] Miller's pyramid of clinical competence describes the transition from a novice, who gathers facts and interprets, to an expert, who demonstrates learning and integrates the facts into practice.[10] Integrating these frameworks, we developed tools to assess learner knowledge and attitudes. A 17-question quality improvement and patient safety knowledge test was developed after an extensive literature review to reflect module goals and objectives. A validated, 12-question attitudes survey was administered before and after the modules.[6] Appendix C contains the knowledge test questions and answers, and Appendix D includes the attitudes survey.

Of the 57 eligible residents, 42 completed the knowledge pretest, and 20 completed the posttest. Statistical analysis used unmatched/unpaired data comparisons of pre- and posttest scores due to the variance in subject size for each test. All scores were included in the tests' individual data sets (i.e., 42 pretest scores and 20 posttest scores). Significant differences were seen between pre- and posttest knowledge results regardless of the statistical test utilized. Mean posttest results (M = 91.00 [± 9.12]) were considerably higher than mean pretest scores (M = 75.24 [± 11.74]) when utilizing the independent t test (p < .001; see Table 1). Comparisons of median values using the Mann-Whitney test also resulted in the same finding, with posttest results (90.0 [82.50, 100.0]) higher than pretest scores (75.0 [70.0, 80.0]; see Table 2).

Table 1. Unmatched Comparisons of Knowledge Pretests and Posttests Using the Independent *t* Test

Test	*N*	*M* (*SD*)	*t* (*df*)	*p*
Pretest	42	75.24 (11.74)		
Posttest	20	91.00 (9.12)		
Pretest vs. posttest			−5.780 (47.189)	<.001

Table 2. Unmatched Comparisons of Knowledge Pretest and Posttests Using the Mann-Whitney Test

Test	*N*	*Mdn* (IQR)	*z*	*p*
Pretest	42	75.0 (70.0, 80.0)		
Posttest	20	90.0 (82.5, 100.0)		
Pretest vs. posttest			−4.469	<.001

Abbreviation: IQR, interquartile range.

A validated, 12-question attitude survey from the Quality Assessment and Improvement Curriculum was administered.[6] Of the 57 eligible residents, 11 completed the attitude presurvey, and 13 completed the attitude postsurvey. Median responses from the survey mostly fell within the 2-3 range of slightly to moderately comfortable. Significant differences showing improvement between presurvey and postsurvey time frames were found in identifying and comparing best practices (p = .02), using the PDSA model (p = .002), and identifying how data are linked (p = .001; see Table 3).

Table 3. Unmatched Comparisons of Attitudes Presurvey and Postsurvey

Question[a]	Presurvey *Mdn* (IQR)[b]	Postsurvey *Mdn* (IQR)[c]	*p*[d]
How comfortable were you with the following?			
1. Writing a clear problem statement (goal, aim).	3.0 (2.0, 3.0)	3.0 (2.5, 3.0)	0.35
2. Applying the best professional knowledge.	2.0 (2.0, 3.0)	3.0 (2.0, 3.0)	0.87
3. Using measurement to improve your skills.	3.0 (2.0, 3.0)	3.0 (2.0, 3.0)	0.47
4. Studying the process.	2.0 (2.0, 3.0)	2.0 (2.0, 3.0)	0.18
5. Making changes in a system.	2.0 (2.0, 2.0)	2.0 (2.0, 3.0)	0.49
6. Identifying whether a change leads to an improvement in your skills.	2.0 (2.0, 3.0)	3.0 (2.0, 3.0)	0.28
7. Using small cycles of change.	2.0 (1.0, 3.0)	3.0 (2.0, 3.0)	0.06
8. Identifying best practices and comparing these to your local practice/skills.	2.0 (2.0, 2.0)	3.0 (2.0, 3.0)	0.02
9. Implementing a structured plan to test a change.	2.0 (2.0, 3.0)	3.0 (2.0, 3.0)	0.09
10. Using the PDSA model as a systematic framework for trial and learning.	2.0 (1.0, 2.0)	3.0 (2.0, 3.0)	0.002
11. Identifying how data is linked to specific processes.	2.0 (1.0, 2.0)	3.0 (2.0, 3.0)	0.001
12. Building your next improvement upon prior success or failure.	2.0 (2.0, 3.0)	3.0 (2.0, 3.0)	0.09

Abbreviations: IQR, interquartile range; PDSA, plan, do, study, act.
[a]Answer scale: 1 = *Not at all*, 4 = *Extremely.*
[b]*N* = 11.
[c]*N* = 13.
[d]*p* value was calculated using the Mann-Whitney test.

Curriculum Assessment

The goal of this curriculum was to give residents an understanding of quality improvement and patient safety principles as well as the tools to apply these principles to practice.

Our hypothesis was that residents and faculty facilitators would choose to participate in more quality improvement–related activities and that resident knowledge and attitudes would improve after completing these modules. This curriculum was evaluated using the following metrics:

- Pre- and postscore comparisons on knowledge, skills, and attitude surveys over time. Residents' attitudes and skills statistically improved.

- Number of quality improvement projects that residents or faculty participated in. After completing these modules, residents or faculty participated in four projects aligned with the curriculum.
- Scholarly output of faculty or residents after participating in this curriculum. Of the above four projects, all were presented at peer-reviewed local or national conferences.

Finally, we elicited feedback after every module and made improvements based on responses.

Discussion

We developed these four modules to address the requirements of ACGME and the needs identified by our pediatric resident learners. The modules provide didactics on quality improvement, patient safety, and evidence-based practice. These didactics can be given alone or as a supplement to a project-focused curriculum.

The results of the knowledge and perception surveys suggest that resident knowledge and attitudes improved after completing the curriculum. Even though the results were significant, the numbers of participants in the attitude survey were low; residents who participated in the curriculum did have the option to not participate in the research portion.

Moreover, there were several lessons learned while developing this curriculum and determining the ideal setting for implementation. Key stakeholder engagement (resident program leadership, learners, and faculty) was essential early in the initiative to promote buy-in and sustainability of the program. Additionally, learner involvement early in the program assisted with needs assessment, curricular design, and evaluation of the program, specifically, placement of the curriculum in an already stretched learner group. Facilitator engagement was also key to success; hospital medicine faculty were already expected to do quality improvement work so this project served as an accessible platform.

Future directions for development include combining these modules with simulated quality improvement projects, development of more robust evaluation tools including qualitative assessments of learners and faculty, and linking learning knowledge and skill acquisition to patient outcomes.

Diana Stewart, MD, MBA: Assistant Professor and Hospitalist, Departments of Pediatrics and Internal Medicine, Baylor College of Medicine

Cara Lye, MD: Assistant Professor and Hospitalist, Departments of Pediatrics, Baylor College of Medicine

Michelle Lopez, MD, MPH: Assistant Professor and Hospitalist, Departments of Pediatrics, Baylor College of Medicine

Brent Mothner, MD: Assistant Professor and Hospitalist, Departments of Pediatrics, Baylor College of Medicine

Elizabeth Camp, PhD: Instructor and Statistician, Departments of Pediatrics, Baylor College of Medicine

Joyee Vachani, MD, MEd: Assistant Professor and Hospitalist, Departments of Pediatrics, Baylor College of Medicine

Disclosures
None to report.

Funding/Support
None to report.

Prior Presentations
Material from this publication has been presented at workshops, including at Pediatric Academic Societies and Pediatric Hospital Medicine conferences.

Ethical Approval
This publication contains data obtained from human subjects and received ethical approval.

References

1. Craig MS, Garfunkel LC, Baldwin CD, et al. Pediatric resident education in quality improvement (QI): a national survey. *Acad Pediatr*. 2014;14(1):54-61. http://dx.doi.org/10.1016/j.acap.2013.10.004
2. ACGME program requirements for graduate medical education in internal medicine. Accreditation Council for Graduate Medical Education Web site. http://www.acgme.org/portals/0/pfassets/programrequirements/140_internal_medicine_2016.pdf. Accessed February 18, 2015.
3. Clinical Learning Environment Review. CLER pathways to excellence: expectations for an optimal clinical learning environment to achieve safe and high quality patient care. Accreditation Council for Graduate Medical Education Web site. http://www.acgme.org/acgmeweb/Portals/0/PDFs/CLER/CLER_Brochure.pdf. Accessed April 23, 2015.
4. Mann KJ, Craig MS, Moses JM. Quality improvement educational practices in pediatric residency programs: survey of pediatric program directors. *Acad Pediatr*. 2014;14(1):23-28. http://dx.doi.org/10.1016/j.acap.2012.11.003
5. Djuricich A. A continuous quality improvement (CQI) curriculum for residents (out of print). *MedEdPORTAL Publications*. 2007;3:468. http://dx.doi.org/10.15766/mep_2374-8265.468
6. Reed D, Wittich C, Drefahl M, McDonald F. A quality improvement curriculum for internal medicine residents. *MedEdPORTAL Publications*. 2009;5:7733. http://dx.doi.org/10.15766/mep_2374-8265.7733
7. Tapper E, Sullivan A, Tess A. Teaching quality improvement on the wards: how we do it. *MedEdPORTAL Publications*. 2015;11:10211. http://dx.doi.org/10.15766/mep_2374-8265.10211
8. Tad-y D, Price L, Cumbler E, Levin D, Wald H, Glasheen J. An experiential quality improvement curriculum for the inpatient setting—part 1: design phase of a QI project. *MedEdPORTAL Publications*. 2014;10:9841. http://dx.doi.org/10.15766/mep_2374-8265.9841
9. Kirkpatrick DL. *Evaluating Training Programs: The Four Levels*. San Francisco, CA: Berrett-Koehler Publishers; 1994.
10. Miller GE. The assessment of clinical skills/competence/performance. *Acad Med*. 1990;65(9):S63-S67. http://dx.doi.org/10.1097/00001888-199009000-00045

Received: April 24, 2016 | **Accepted:** September 16, 2016 | **Published:** October 13, 2016
