# Supplementary material for: Engaging Learners Through Modules in Quality Improvement and Patient Safety
Source: MedEdPORTAL. 2016 Oct 13;12:10482. doi: 10.15766/mep_2374-8265.10482 (PMC6440404; doi:10.15766/mep_2374-8265.10482)
Supplement: Supplementary file 1 — A. Instructor's Guide.docx B. PowerPoint Talking Points.docx C. Knowledge Survey.docx D. Attitude Survey Questions.docx E. Fundamentals of QI.pptx F. Fundamentals of Patient Safety.ppt G. Evidence-Based Practice and QI Improvement Research.pptx H. QI and PS Potpourri.pptx [file mep-12-10482-s001.zip › A. Instructor's Guide.docx]

**Appendix A. Instructor’s Guide**

**Background**

Residents are on the frontlines of medical care in academic institutions. Their daily interactions are crucial to the quality of care received by patients in these settings, and thus knowledge of patient safety and quality improvement are essential. The Accreditation Council for Graduate Medical Education (ACGME) requires all residents to participate in quality improvement (QI) and patient safety (PS) programs as part of their residency training. The ACGME Clinical Learning Environment Review (CLER) provides programs with periodic feedback regarding trainee education in 6 areas, two of these being patient safety and quality improvement. Recently, the Association of Pediatric Program Directors developed a survey of QI curricula for program directors and found they were rarely longitudinal (12%) and the major barriers were time (66%), funding (39%), and lack of QI expertise (33%). To meet this need we developed a curriculum in patient safety and quality improvement for pediatric residents. This curriculum addresses all of these barriers allowing faculty with minimal QI training, limited time, and minimal resources to teach QI.

We reviewed the MedEd portal submissions available on this topic. Djurich et. al and Reed et. al both outline curricula which focus on the model for improvement and incorporate a simultaneous quality improvement project. Tapper et al. outlines a curriculum to teach QI on wards using a longitudinal iterative approach focusing on a QI project in an ambulatory rotation while including evaluations that use CLER specific language. Tad-y et al. provides a handbook of resources that can be used to implement QI curricula. Most of these focus on an experiential component. Our curriculum describes short modules that can be given as a supplement to a project-focused curriculum or can stand alone. While previous curricula have focused on QI only, these modules are not limited to quality improvement but also teach elements of patient safety and evidence based practice, realizing that all three topics operate along a continuum. In order to keep patients safe, the care we provide must be continuously assessed and improved, while incorporating new evidence.

**Target Learner**

The target learners for this curriculum are pediatric residents of all post-graduate levels; however, this curriculum can be easily adapted to any residency training program. Additionally, other learner groups would also benefit from this tool, including medical students and interprofessional trainees (i.e. nurses, physician assistants, etc.). We currently give these lectures to our first year pediatric residents.

**Instructor Qualification**

At our institution, this curriculum is taught by pediatric hospital medicine faculty with varying quality improvement expertise. This curriculum can be taught by faculty at any level (instructor to professor). Non-physicians who are involved in quality improvement such as nurses, other non-physician clinicians, or administrators should be able to teach it as well. Instructors who have not received any quality improvement training should consider doing the online quality improvement modules at the Institute for Health care improvement open school ([www.ihi.org](http://www.ihi.org)) as a primer; however, this curriculum can be utilized without this pre-requisite.

**Goals and objectives**

Using core features of adult education theory and QI/PS methodology, we developed modules that would provide residents fundamentals of quality improvement and patient safety to increase their ability to apply learned principles into practice.

After completing this curriculum, residents will be able to:

- Define the model for improvement
- Explain components of an aim and strategies to test small cycles of change
- Compare and contrast adverse and sentinel events
- List types of medical error
- Describe how evidence based practice fits into quality improvement
- Systematically analyze current practice using quality improvement methods
- Review how effective leadership and multidisciplinary teams promote safe care
- Apply given tools to educate health care professionals on quality and safety issues
- Integrate learned concepts into future quality improvement initiatives at their institution

**Education Methods**

Residents will receive education using the following teaching methods:

- PowerPoint presentations
- Small group discussions with case discussions will supplement learning
- Feedback and reflection with course facilitators as materials are presented

This curriculum consists of four modules. These modules are currently given at our institution during an ambulatory rotation for first year pediatric residents. There are 4 modules, and each module is 30 minutes or less. These modules can be given during one rotation, throughout residency, or partnered with a practical application such as a project. Below is a detailed description of each module. The talking points of the presentations are included in Appendix B.

**Module 1. Fundamentals of Quality Improvement.** The goal of this module is to provide a basic understanding of QI. It reviews the Institute of Medicine (IOM) 6 aims for improvement as well as the Institute for Health Care Improvement (IHI) Model for improvement. After completing this module residents will be able to:

- Describe why QI is important
- Recite the Institute of Medicine 6 aims for improvement
- Define the model for improvement
- Outline components of an aim and strategies to test small cycles of change using the Plan Do Study Act (PDSA) cycle

**Module 2. Fundamentals of Patient Safety.** The goal of this module is to provide a basic overview of patient safety. This module explores how medical error is a leading cause of death in our country and discusses methods at our institution to reverse this alarming statistic. After completing this module, residents will be able to:

- Define adverse and sentinel event
- List types of medical error
- Outline how to file an incident report at their institution

**Module 3. Evidence Based Practice and Quality Improvement Research.** The primary goal of this module is to define evidence-based practice (EBP), introduce basic concepts of EBP and QI research, and discuss how both of these concepts can be applied to daily patient care. After completing this module residents will be able to:

- Define evidence-based practice (EBP) and apply it to QI
- Identify and know how to access appraisal tools
- Recognize different types of QI studies

**Module 4. Quality Improvement and Patient Safety Potpourri.** This module includes 3 topics: QI and health care policy, leadership and QI, and team effectiveness and QI. Many learners do not understand how quality improvement initiatives impact society and populations locally, nationally, and globally, however, healthcare policy can dictate and guide the aims and metrics of an institution. Trainees must also recognize effective leadership and teams are essential to a successful quality improvement initiative, promote a culture of safety, and decrease medical errors. After completing this module residents will be able to:

- Discuss how QI impacts healthcare policy
- Examine qualities of an effective QI leader and team
- Recognize how effective leadership and multidisciplinary teams promote and enhance safe care

**Alternatives**

We deliver this curriculum during an ambulatory rotation where the learner has decreased clinical responsibilities. Given the short nature of the modules, this curriculum can be employed on any rotation, potentially even during scheduled conference time. This curriculum can also be delivered in a classroom setting. Other alternatives include using technology.

**Curriculum Implementation**

The resources needed for this curriculum are: faculty and resident time, laptop/projector, space. Each module is about 30 minutes; faculty will need to allocate 1.5 hours to teach all four modules. Additional prep time is needed prior to the module to familiarize themselves with the material. The prep time would vary for each faculty member. Average prep time at our institution was about an hour which decreased with subsequent lectures. Residents are scheduled to receive the lectures during an outpatient ambulatory rotation; they do not have any clinical responsibilities while they are receiving the didactics. A block of time will need to be allocated for residents to receive the didactics. A laptop or computer connected to a project is needed to run the PowerPoint.

**Facilities**

The room set-up would depend on the number of participants. At our institution, we deliver this material in an intimate setting, average 3 to 5 residents, but these modules can be given to a larger audience. We recommend limiting the session to 30 learners, thus maintaining the reflection and discussion aspect of each module. The optimal room set-up would be a room in which the learners are in a circle or oval (multiple circles if you have a bigger class); this will allow learners to participate in the discussion and be able to visualize the PowerPoint.

**Advance Preparation**

The facilitator should review the PowerPoint and script. We also recommend the Institute of Health Care Improvement ([www.ihi.org](http://www.ihi.org)) open school modules, specifically the Fundaments of QI and Fundamentals of Patient Safety modules (optional)

**Lessons Learned**

The key to any successful project is key stakeholder engagement (resident program leadership, learner, and faculty) early in the initiative to promote buy-in and sustainability of the program. Additionally, learner involvement early in program assisted with needs assessment, curricular design, and evaluation of the program specifically placement. Initially modules were given during busy inpatient ward months; this was gradually moved to an outpatient ambulatory month based on learner feedback with dedicated time for modules. Facilitator engagement was also key to success; hospital medicine faculty were already expected to do QI work so this project served as an accessible platform.
