# Supplementary material for: Engaging Learners Through Modules in Quality Improvement and Patient Safety
Source: MedEdPORTAL. 2016 Oct 13;12:10482. doi: 10.15766/mep_2374-8265.10482 (PMC6440404; doi:10.15766/mep_2374-8265.10482)
Supplement: Supplementary file 1 — A. Instructor's Guide.docx B. PowerPoint Talking Points.docx C. Knowledge Survey.docx D. Attitude Survey Questions.docx E. Fundamentals of QI.pptx F. Fundamentals of Patient Safety.ppt G. Evidence-Based Practice and QI Improvement Research.pptx H. QI and PS Potpourri.pptx [file mep-12-10482-s001.zip › B. PowerPoint Talking Points.docx]

**Appendix B. Presentation Slide Talking Points**

Below are the scripts of each module by slide. We recommend reviewing these scripts before giving the module.

**MODULE 1: Fundamentals of Quality Improvement Power Point Scripts**

Instructors: One instructor is needed to facilitate and to deliver the content.

Learners: Interns

Media Requirements: Computer with internet access and PowerPoint capabilities

Time Allotment: minimum of 30 min (can be longer based on discussion)

Implementation tips for faculty: This module is aimed at providing very basic concepts in QI. It reviews the Institute of Medicine (IOM) 6 Aims for Improvement as well as the Institute for Health Care Improvement (IHI) Model for improvement. It is important that you are familiar with these topics so that you can answer any questions your learners may have.

Session Content:

Slide #1 – Intro slide: Hello, welcome to module 1: Fundamental Quality Improvement (QI) Concepts.

Slide #2 – This module is the 1^st^ of 4 the Quality Improvement and Patient Safety (QI/PS) Curriculum. The modules that will follow are: Fundamental Patient Safety (PS) Concepts, Evidence Based Practice and QI Research, and QI/PS Potpourri.

Slide #3 – Please take a moment and review the goal for this module. Speaker pauses ~12 sec to read the goal- Using this online learning module, first year pediatric residents will understand fundamentals of quality improvement including being able to identify and perform the components of a PDSA cycle and generate a SMART Aim statement based on the established guidelines.

Slide #4 – The objectives for this module include the following: Describing QI and why it’s important, reciting the Institute of Medicine 6 aims for improvement, defining the model for improvement, generating a SMART Aim statement, and identifying and performing the components of a PDSA cycle.

Slide #5 – Outline: in order to achieve these goals and objectives for this module we will discuss the following: Why is QI important? What can you do? In order to answer this 2^nd^ question, we will look at the IOM’s 6 aims for Improvement as well as the IHI’s Model for improvement, which include the PDSA cycle and creating a SMART Aim statement.

Slide #6 – So let’s begin…Why is QI important?

Slide #7 – Why is QI Important continued: According to the AAP EQUIPP QI basics module, QI uses a formal and systematic approach to continuously improve patient care in a measurable way. Additionally, patients and healthy outcomes depend on the quality of care received.

Slide #8 – Why is QI Important continued: According to the IOM’s to Err is Human from 1999, Deaths in US hospitals are attributable to medical error in 44,000-98,000/year. This translates to an estimated cost of $17-50 billion. More recent estimates in 2013 approximate 200,000 deaths. In 2016, medical error was deemed to be the third leading cause of death by a study done at John Hopkins.

Slide #9 – Why is QI Important continued: Finally, let’s take a look at the American Customer Satisfaction index. Where do hospitals stand on a scale of 0 to 100? List on screen shows Soft drinks, Internet Portals & search engines, hospitals, federal government, and airlines. After question is posed, “hospitals” are highlighted in red showing score of 77, above federal government and airlines but below soft drinks and internet portals and search engines. All of this translates to a growing dissatisfaction for the health care system and has significant implications for quality and safe patient care.

Slide #10 –We see now why QI is so important but what can you do?

Slide #11 – IOM 6 Aims for Improvement: Let’s start by reviewing the 6 Aims of Improvement or domains of quality as described in the IOM’s 2001 report, “Crossing the Quality Chasm”. You can remember the 6 Aims with the pneumonic “STEEEP”: Safe Timely Effective Efficient Equitable Patient-centered.

Slide #12 – Case Scenario: Let’s look at a case scenario. One of our own Texas Children’s Pediatric residents identified a problem (This can also be replaced with a local case at your institution for added learning). There was no clear way for residents to get in touch with other residents. Let’s look at this from a QI perspective. Think about how we can create a QI project that delivers safe, timely, effective, efficient, equitable, and patient-centered quality care.

Slide #13 – Next, let’s look at the IHI’s model for improvement. This model provides a systematic approach to plan, test and implement changes and has been used extensively in both health care and non-health care settings to implement process changes quickly and effectively. (Speaker then reviews in step-wise fashion the image of the model) The first step in this model is the AIM: what are we trying to accomplish? The 2^nd^ step are the MEASURES: How will we know a change is an improvement? The 3^rd^ step are the CHANGES: What changes can we make that will result in improvement. The final step is the PDSA CYCLE, which stands for Plan Do Study Act.

Slide #14 – Creating a SMART Aim Statement: The first step in the Model for Improvement and for our QI project is to create a SMART Aim statement. Aim statements should be Specific- what is the goal or intent. Focus on achieving only one thing and as you formulate your question, consider drivers and stakeholders in the project. They should be Measurable- so that you can monitor your progress over time. They should be Actionable- so the team can take action to overcome known barriers and achieve proposed results. An aim statement should also be Realistic- so that we can use the given resources and achieve the projected results. Finally, an Aim Statement should be Timely- set a target date and interim milestones in between so that the project can be completed.

Slide #15 – Case Scenario: Aim Statement: So going back to our case scenario, what would your AIM statement be for this project? (You can write what they come up with on the board). Is it SMART? Improve the proper identification of resident (or team) by 70% on the hospitalist service to begin Sept 2016

Slide #16 – Case Scenario: Measures: What would you measure to assess the situation? (Some examples are % of hospitalist patients with correct resident assigned; survey nurses to determine how many times they contact the incorrect team and assess this throughout your interventions; survey nurses to assess satisfaction with new process of assigning resident teams). You may also add or delete measurements based on your intervention (see talking points for slide #19)

Slide #17 – Case Scenario: PDSA cycle. Now let’s continue with the PDSA cycle. What are the next steps you’d consider? Identify one change that might we worth testing. (Have the residents think about this as you move on to Slide #18 and explain the PDSA cycle). Some examples may include signs with assigned resident name or adjusting the computer system to include resident assigned next the patient name.

Slide #18 – Case Scenario: PDSA cycle (Image of PDSA cycle with description of each component, color coded). Now that you have set your Aim, established measures, and selected your ideas for change, think about the steps you will take. Al of these previous steps were in the ‘Plan’ part of the PDSA cycle. For the ‘Do’, try your change on a small scale. For example, with a few patients, a pilot unit, or for a short time and see what happens. For the ‘Study’, evaluate the changes you try and see if they result in improvement by comparing the results to the baseline and the goal. For ‘Act’, adopt, reject, or modify the change so that the next cycle can begin. Decide what you want to do differently in this next cycle.

Slide #19 – Back to our Case Scenario: So let’s go back to our case scenario. What happened to our highly motivated Pediatric Resident and her first PDSA cycle? She decided to clearly identify team signs on each door with colors, pictures, and contact numbers on all the doors in a pilot unit on the 7^th^ floor where all the Pediatric Hospital Medicine patients are. The initial response has been great with plans to expand and look at the data.

Slide #20 – Summary: QI is important for a number of reasons. You can do things to advocate for quality patient care in your everyday practice via dedicated QI projects. When doing these projects, remember the IOM 6 Aims for Improvement as well as the IHI’s model for improvement. Keeping in mind the PDSA cycle and how to create a SMART Aim statement.

Slide #21 – Thank you for your attention and time. We hope we have provided you with some fundamentals in QI so that you can continue to provide quality and safe patient care in your practice.

**MODULE 2: Fundamentals of Patient Safety Power Point Scripts**

Instructors: One instructor is needed to facilitate and to deliver the content.

Learners: Interns

Media Requirements: Computer with internet access and PowerPoint capabilities

Time Allotment: minimum of 30 min (can be longer based on discussion)

Implementation tips for faculty: The primary purpose of this module is to define the fundamentals of patient safety, medical errors, and the communication to patients and families following a medical error. Learners may have different level of exposure to these topics, so it is important to be familiar with the PowerPoint in order to answer any questions that may arise.

Session Content:

Slide #1 – Welcome to the module. Today we will discuss patient safety, medical errors, and the communication to patients following an error.

Slide #2 - Please take a moment to review the goals for this presentation. Then pause to allow them time to read (~ 7 sec). The goals will be to understand the concept of patient safety, the exploration of medical errors, and to discuss the importance of communication and the response to medical errors.

Slide #3 - Please take a moment to familiarize yourself with the objectives. Then pause to allow them time to read (~10 sec). The objectives are to define adverse and sentinel events, list three types of medical errors, and demonstrate how to file an incident report at your institution (This can be tailored to your institution based on what’s available).

Slide #4 - People often make comments like “I’m only human.” or “Nobody’s perfect.”

The Institute of Medicine recognized the fallibility of humans and released a report entitled “To Err is Human” in 1999. This report explored the reasons why medical errors should not be blamed on one individual. To err is human…. humans will make mistakes. Healthcare systems need to be designed with safeguards to prevent against one mistake resulting in a medical error.

Slide #5 - Before we continue, it is important to be familiar with a few of the common definitions. First-As we explore patient safety today, we are focusing a great deal on medical errors. The definition of an error may be apparent, but remember that it is not only the commission-doing something wrong- but also the act of omission (failing to do the right thing). It is much more difficult to recognize and prevent acts of omission.

These acts of commission or omission can lead to either a near miss or an adverse event. In a near miss, there is potential for an undesired outcome but by chance the adverse event does not occur. In patient safety, it is essential to learn from these “close calls” in order to prevent adverse events.

The definition of adverse event is broad and includes any injury caused by medical care. Therefore, errors can lead to adverse events, but adverse events are not always caused by errors. The injury can result from any aspect of diagnosis or treatment.

When an adverse event is caused by an error and leads to serious harm or death, it is called a sentinel event. These events are understood to be egregious and the faults in the system which allowed such an event to occur must be reviewed.

Slide #6 - One way to explain faults in a system is the “Swiss Cheese Model”.

Slide #7 - In an ideal healthcare environment, there would be no holes and every component of healthcare would be intact. However, because humans err, there will be holes. A well-constructed healthcare system would have blocks at the next slice so that these errors would not progress any further and the holes would not result in an adverse event. However, there are times when the many layers line up perfectly and result in an opportunity for an adverse event. When you have holes in your defenses (layers), they result in harm to the patient. The goal is build a system with many defense layers that will stop errors from reaching the patient.

Slide #8 - Consider a Morphine dosing error being transmitted through the various layers of a hospital system and resulting in an adverse event with a patient overdosing and requiring Naloxone.

What are examples of the layers in a hospital system that are built upon each other to prevent such an error from occurring?

In this example, the physician had to make two errors. The physician ordered the wrong dose and ignored the computerized warning. The computer warning is an example of a system solution, which we will discuss more later. Previously, when using paper charts, the physician had no check at the time of ordering the medication.

Another point to think about…there are many reasons that the pharmacy may have dispensed the medication as written or that the nurse would administer it. One of these reasons may be a fear of speaking up. As busy physicians, when a pharmacist or nurse calls to check on one of our orders, we many feel frustrated. However, it is important to remember that they are doing their job and serving as a defense against a medical error. We should be patient and appreciate their role in ensuring patient safety

Slide #9 - There are many types of medical errors. We have explored one example of a medication error using the Swiss cheese model. We will further explore the following:

Slide #10 – Medication Errors

Slide #11 - One key part of patient safety is to find ways to decrease the chance of people making mistakes. Traditionally, infant’s acetaminophen has come in a more concentrated form of 80 mg per 0.8 ml, which equals 500 mg per 5 ml. Children’s acetaminophen has come in 160 mg/5 ml. Several reports were filed of parents mistaking the two concentrations and as a result giving 3 x the intended dose. In 2009, manufacturers agreed to move to a standard concentration for infant and children’s formulations. The new infant’s acetaminophen became available in late 2011.

Slide #12 - Please take a moment to review possible solutions in medication errors.

Slide #13 - Did you ever play the telephone game as a kid? It basically involved a message being passed along to several people. At the end of the game, you compared the original message to the last one received. They rarely, if ever, matched up. Now take a moment to count how many times a patient is handed off on a normal day…. when you have clinic…. when you are off; now consider the times when you’ve received a direct admission to the hospital medicine service from another institution, how well do you understand the patient’s situation when they first arrive? Do you feel confident that you have received all of the essential information needed to care for that patient?

Each time a patient is handed off there is the potential to miss the communication of vital information

Slide #14 - Healthcare literature has documented that work hour restrictions have led to an increased number of handoffs. These errors can happen at all levels including from person to person and from site to site. Successful handoffs strategies include having designated times, minimal distractions, and the discussion of likely scenarios. Again, information technology will be key to solutions along with possibly the implementation of standard protocols.

Slide #15 – Next Session is on Diagnostic Errors.

Slide #16 – Types of Diagnostic Errors. Please take a moment to read the definitions for these various

types of diagnostic errors.

Slide #17 - Imagine if you were involved in the care of a family who experienced a medical error that resulted in

death of their child. How would you approach them knowing that you and your team were wrong about his diagnosis

and knowing that this patient was in critical condition? What would you say? How would you apologize? We will

review why communication is important, how to break bad news, and how to approach the apology.

Slide #18 - If you sat for a moment on the previous slide and put yourself in the position of the medical team taking care of this child, you might be able to imagine how they felt. Why is it so hard to tell families the truth? In a book entitled medical errors and medical narcissism, the authors explored some of the psychological reasons for the difficulty we experience communicating after adverse events. Can read the other reasons from the slide.

Slide #19 - When faced with the difficult situation of having to tell a family about an error, it is essential to remember why what you are doing is so important. Not only is communicating openly with patients the right thing to do, it will likely benefit the patient and the caregiver by addressing their suspicions. This may allow them to trust in the honesty of their team. It is part of our professional code of conduct and being honest and open with patients has been shown to decrease the risk of lawsuit. Finally, it is just the right thing to do.

Slide #20 - The following is one possible approach to dealing with medical errors. Take a moment to review the steps. (Speaker to pause for about 5 sec).

In your initial communication, speak clearly, slowly, and directly. Pause often to allow the listeners to collect their thoughts. In the initial discussions with parents, do not speculate on the cause of an error. It is important to acknowledge that the event occurred and to express empathy and compassion, whether or not you know what caused the event. You should also let the family know what will be done to make the error less likely to occur in the future. It’s important to reassure patients and families that you have the same goal.

Slide #21 - At our institution the event reporting system is called **Safety Scoop** (Discuss your event reporting system at your institution). An online format available to all employees, it is easily accessed and reports are delivered to the Quality and Safety Department for review. These reports are then filtered to leadership of the responsible departments within the hospital.

Slide #22 - Once the report is reviewed, any deviation in care that reaches the patient is reviewed by Intense or Root Cause analysis; an in-depth multi-disciplinary care team analysis of the event and the steps that led to the event. A confidential phone hotline is also available for reporting any concerns by staff. (Insert initiatives at your institution here).

Slide #23 - Creating and sustaining a culture of safety and change is imperative to achieving improvement within a system. People are encouraged to take action when change is needed. Leadership at all levels must be committed to improvement and provide support and the resources needed.

Slide #24 – Summarizes the points covered in this module. Please read each point.

**MODULE 3: Evidence Based Practice and Quality Improvement Research Power Point Scripts**

Instructors: One instructor is needed to facilitate and to deliver the content.

Learners: Interns

Teaching Methods: self-guided online PowerPoint modules with voice-overs

Media Requirements: Computer with internet access and PowerPoint capabilities

Time Allotment: minimum of 30 min (can be longer based on discussion)

Implementation tips for faculty: The primary purpose of this module is to define evidence-based practice (EBP) and introduce basic concepts of EBP and QI research. Learners may have different exposure depending to these topics, so it is important to be familiar with the PowerPoint in order to answer any questions that may come up.

Session Content:

Slide #1 – Welcome to the module and today we will be exploring Evidenced based practice and Quality Improvement Research. Throughout this presentation evidence based practice and evidence-based medicine will be used synonymously.

Slide #2 – Please take a moment to review the goals for this presentation. Then pause to allow them time to read (~ 7 sec). Goals: Provide and introduction to evidence-based practice (EBP), help the learner identify ways to incorporate EBP into his or her daily patient care, and provide an introduction to research in quality improvement.

Slide #3 – Please take a moment to review the objectives (~ 10 sec). Objectives: Define evidence-based practice (EBP), Formulate a PICO question, Identify and know how to access one literature source other than PubMed, Identify and know how to access one appraisal tool, recognize different types of QI studies, and recognize how EBP and QI differ and how they complement each other in practice.

Slide #4 – What is Evidence Based Practice? Speaker gives learner ~13 sec to read the definition themselves then begins to explain. Speaker then reviews 2 key points that are highlighted in blue on the slide: *individual clinical expertise and best available external clinical evidence.*

Slide #5 – How do we practice EBM? Speaker introduces the 5 steps for practicing EBM proposed by Straus et al in 2011- “Evidence based practice and how to teach it”. Then, there is a pause for ~15 sec to allow learner to read through image of the 5 steps on their own. Step 1- Convert the need for information into an answerable question. Step 1- Track down the best evidence with which to answer that question. Step 3- Critically appraise it for its validity, impact, and applicability. Step 4- Integrate the critical appraisal with your clinical expertise and with your patient’s unique biology, values, and circumstances. Step 5- Evaluate your effectiveness and efficiency in executing steps 1-4 and seek ways to improve them both for next time.

Slide #6 – EBP in action: Provides an example of a patient scenario that can be used for EBM. A mother wants to know if inhaled steroids for asthma will stunt her daughter’s growth.

Slide #7 – Step 1- Formulate the question. Introduces concept of PICO and defines each part: Population- group being investigated. Intervention- therapy, test, or any other interventions done to patient. Comparison- made to group of patients who did not have intervention done. This step is frequently omitted because it is not always practical to include. Outcome- effect on patient- time to resolution of illness, long-term effects, length of hospital stay, mortality, and so on.

Slide #8 – Step 1 continued: Let’s formulate a PICO question for our clinical scenario.

Population = children with asthma Intervention = inhaled steroids Control = children not receiving inhaled steroids Outcome = adult height

Putting it all together, our PICO question becomes: In children with asthma, does long-term inhaled steroid use lead to decreased adult height? (Note: the comparison to non-use of inhaled steroids is implied).

From the PICO question we can pick out the key terms and phrases when we go to make our search. The key words/phrases are: children, asthma, inhaled steroids, adult height. Sometimes we have to play around with search terms depending if initial search needs to be broader or narrower. Some trial and error is involved with search.

Slide #9 – Step 2: Track down the best evidence. There’s more to life than PubMed. Speaker introduces that she would like to draw learner to other resources.

Slide #10 – Cochrane Collaboration Database. [www.cochrane.org](http://www.cochrane.org). Contains systematic reviews of primary research in human health care done by a group experts looking to answer a clinical question just like you are. All the existing research on specific topic that meets certain criteria is searched for and collated and assessed using stringent guidelines to establish whether or not there is conclusive evidence about a specific treatment. Reviews are updated regularly. If there is one available on your topic you are in luck because someone has already done much of the work for you. You can reach this database through the Texas medical center library. As a resident you can apply for online access to this database and others. Slide then shows picture of TMC Library. (This is our local library, please insert the best way for your learners to access it. If there is no direct available, consider putting a screen shot of www.cochrane.org).

Slide #11 – Agency for Healthcare Research and Quality (AHRQ) - [www.ahrq.gov](http://www.ahrq.gov). There is a screen shot of the AHRQ homepage. Collects reports from dedicated EBP centers who review all relevant scientific literature on clinical behavioral and organization and finance topics to produce evidence reports that are that are then made available to practicing physicians. The results are organized by topic.

Slide #12 – National Guideline Clearinghouse. [www.guideline.gov](http://www.guideline.gov). There is a screenshot of the homepage. Collection of all clinical guidelines organized by topic and searchable for by keyword.

Slide #13 – Additional guidelines: published by individual organizations. Examples: AAP Guidelines, University of Michigan Evidence-Based Practice, Cincinnati Children’s Hospital EB Guidelines, and Texas Children’s Hospital EB Guidelines. *Links to all of these provided on slide except for Texas Children’s. (Consider adding your guideline page if available)

Slide #14 – Let’s go back to our example of newly diagnosed asthmatic. We can do a PubMed search for asthma and inhaled steroids and height, limit to articles English, on humans age 0-18 years. Returns 133 articles. A good starting point.

Compares results using the National Guideline Clearing House. When using terms asthma/height shows 24 guidelines vs asthma/children with 126 guidelines. Searching the University of Michigan shows a critically appraised topic called long-term treatment with inhaled steroids doesn’t affect adult height. Notice use of different search terms in different databases based upon specificity of articles returned.

Slide #15 – Step 3: To critically appraise the evidence. We are not alone in our task. Slide has graphic showing many critical appraisal tools to help us evaluate the quality of the evidence we have found. Please take a moment to review the resources and the studies for which they are used. Includes: CONSORT, CASP, PRISMA, AGREE, GRADE. Speaker pauses ~25 sec to let learner review content of the slide. All tools are available free online.

Slide #16 – Let’s go back to our example. Reviews an interesting article from NEJM from 2000 on our topic. We then review the abstract and see that it is a prospective cohort study and seems reasonably well done.

Slide #17 – CASP tool: Google CASP cohort and print out copy of CASP tool. Slide has image of CASP tool and speaker pauses for ~ 9 sec to allow learner to review content.

Slide #18 – Step 4- Integrate. Integrating what we have learned from evidence with what we know from our own experience, and what we know about our patient and her unique biology and preferences. We consider her age/ethnicity of our patient as well as the severity of her asthma and decide if it’s reasonable to extrapolate the results from the NEJM study to her particular case. We weigh the likelihood for the effect on adult height with the strength of the patient’s desire to achieve maximal adult height along with desirable effects of inhaled steroids on asthma control. All of this is discussed with the family.

Slide #19– Step 5- Reflect and Improve. Think of ways we can improve next time by comparing your findings with others’, refine your search method for next time, and reflect on how long task took us. All this is important for how we as individuals practice evidence-based medicine.

Slide #20 – How can we as an institution perform EBP on a regular basis? One way is through use of evidence based guidelines, commonly called clinical guidelines. Please read the IOM’s definition for clinical practice guidelines below. Speaker pauses ~ 7 sec for learner to read definition. Note that it does not explicitly mention evidence, but we know that the best clinical guidelines will have evidence at their root of their development.

Slide #21 –How can we perform EBP on a routine basis? Through the use of quality improvement (QI) interventions. QI interventions attempt to change clinician behavior and thereby achieve improved patient outcomes. Studies in QI evaluate the effectiveness of those interventions.

Slide #22 – What comprises QI research? Image showing pyramid representing the hierarchy of evidence in QI studies. RCT at the top, anecdotal reports similar to case reports are at the bottom of the research hierarchy. Unfortunately, the current research methodologies in QI intervention studies fall in the bottom half of pyramid. Many factors contribute to this- ex: randomization impractical. However, goal should still be to have QI studies that are performed just as rigorously as primary clinical research.

Slide #23 – Guide for assessing QI articles. Many individuals use the SQUIRE guidelines to formally appraise QI articles. There are some other general guidelines to assess QI articles as delineated in 2010 by Fan et al in their article “How to use an article on QI”. We should always ask ourselves: are results valid? What were the results? How can I apply the results?

Slide #24- EBP vs QI…what’s the difference? Traditionally, evidence based medicine is based upon external research and evidence. It is focused on ‘doing the right things’. Quality Improvement is based more on local processes and focuses on ‘doing things right’. They each have similar goals of improving patient care, but they focus on a different part of the problem. EBM focuses on discovering what the best available evidence for a problem is whereas QI aims to ensure actions are done thoroughly, efficiently, and timely in an effort to improve patient care.

Slide #25 EBP vs QI…what’s the difference? Each face their own unique problems. With EBM, often there is an Evidence-Practice gap: there is an abundance of information and determining what is most valid and relevant can be difficult with the sheer volume. This leads to information overload and makes it very difficult to stay up to date. Additionally, not everyone is equipped with knowledge or skills to determine what is the best evidence available for a certain question or problem. With QI, there tends to be more of a ‘knowing-doing’ gap. We know what we should be doing but we fail to do it or fail to do it correctly as individuals or as an institution

Slide #26 How do EBM and QI relate? In a paper published in 2011 in the BMJ Quality and Safety Journal, Glasziou et al looked at the relationship between EBM and QI and illustrated with this image. As you can see here, a sequence of EBM is followed by QI initiatives ultimately leading to good patient care.

Slide #27 How do EBM and QI relate? Used together, these two techniques can be used to address the research-practice gap. For QI, thorough and accurate review of the data early in the process can drive measures and proposed changes. Questions to be asked are: What does the evidence say is standard of care? What does the evidence say is the best way to achieve this? How can this be applied to my institution? For EBM, QI is needed to disperse and implement information the researcher discovers. Researchers need to be conscious of QI methods when formulating their questions to ensure that what they discover can actually reach the patient and those caring for them. Research should focus on what is the next step of action and not just appraisal to truly make positive impact on patient care.

Slide #28 EBM and QI: Doing right things right. Here you can see visually how EBM and QI relate. We start with a clinical problem, then we apply EBM steps to formulate an answerable question, find the best evidence, critically appraise the evidence, and work to apply it to individual and systems of care. In order to this last step, we must have QI initiatives in place that are based upon the evidence. We must create a clear aim of what we are trying to accomplish, determine strong measures to determine if a change leads to improvement, and manage the changes through multiple PDSA cycles.

Slide #29 – Summary slide: The practice of evidenced based medicine means integrating individual clinical expertise with the best available external clinical evidence from systematic research. Steps to practicing EBM include: 1. Ask the right question (often done in PICO format) 2. Search the literature (through multiple avenues) 3. Critically appraise the literature 4. Apply it to your individual patient 5. Self-assess and improve your technique

Slide #30 – Summary cont’d: There are many appraisal tools available online that help you critically evaluate the literature. Evidence-based guidelines are systematically derived statements to help guide you in patient care. EB guidelines and their associated tools are available on the TCH homepage.

Slide #31 – Studies in QI research attempt to evaluate the effectiveness of interventions designed to change clinician behavior and thereby achieve improved patient outcomes. QI studies should be as rigorously designed, executed, and evaluated as studies in clinical research. The connection between EBP and QI is crucial in implementing sustainable evidence-based change that will positively impact the patient

Slide #32 – List of resources available to you as you practice and improve your evidence-based technique. 5 different sources listed. You can refer back to this module at any time to obtain these references.

Slide #33 – Additional articles used for talk, list of 4

Slide #34– Thank you for your time and best luck

**MODULE 4: Quality Improvement and Patient Safety Potpourri Power Point Scripts**

Instructors: One instructor is needed to facilitate and to deliver the content.

Learners: Interns

Teaching Methods: self-guided online PowerPoint modules with voice-overs

Media Requirements: Computer with internet access and PowerPoint capabilities

Time Allotment: minimum of 30 min (can be longer based on discussion)

Implementation tips for faculty: This module includes 3 different topics: QI and Health Care Policy, Leadership and QI, and Team effectiveness and QI. It is important to familiarize yourself with all three topics so that you can answer any questions that may come up after the session. Additionally, the goal is for the learner to see how all three of these components interact and relate to QI and patient safety.

Session Content:

Slide #1 – Welcome to the QI Potpourri module. This module is made up of 3 mini-modules. The first is Quality Improvement and Health Policy

Slide #2 – Review of the goals: To highlight the importance of QI in health care and to explain the role of physician in Health Care Policy and QI.

Slide #3 – Review of the Objectives: To list 3 examples of how QI impacts health care policy.

Slide #4 – Why is this important? It is crucial for physicians to understand the importance of QI and health care. The evidence suggests that the quality of health care that Americans receives is not ideal, what constitutes good quality health care is not well defined, quality varies widely, and quality of health care varies across populations.

Slide #5 – Quality Varies Across Populations: the disparity of quality among certain populations is one reason why QI is so important. Table on slide illustrates how certain ethnic groups are more or less likely to receive prenatal care based upon ethnicity. Relative risk of 2 means that Hispanic mothers are twice as likely not to have received prenatal care. This is an example of variation in quality across populations.

Slide #6 – Health Policy and QI: It is important for physicians to know how QI has been integrated into health care policy. Effective Health Care Program, the Agency for Healthcare Research and Quality (AHRQ), the Affordable Care Act, and the Health Care Quality Improvement Act of 1986 are good examples of how QI has been integrated into Healthcare policy. We will now talk about some of these in more detail.

Slide #7 – The Effective Health Care Program: works with AHRQ to produce effective research. There are 3 primary products: research reviews, original research reports, and summary guides.

Slide #8 – Research Reviews: one of the primary products of Effective Health Care Program. There are different types and patients can search in many ways, most simply by keywords. These guides can help patients understand conditions and options for treatment as well as risks/benefits of treatments. Making information accessible to patients is one-way health care policy has improved the quality of care that patients receive.

Slide #9 – AHRQ is a division of the US Department of Health and Human Services (HHS). It is the knowledge base for what works and what doesn’t work in health care. Furthermore, AHRQ works to translate this knowledge into everyday practice and policy making. The National Health Care Quality Report and the National Health Care Disparities Report are 2 key products of the AHRQ.

Slide #10 – The AHRQ is our 2^nd^ example of how health policy has improved the quality of healthcare. AHRQ has 2 primary products as mentioned before. AHRQ has been providing these 2 reports since 2003 as a means of highlighting specific areas that need improvement in healthcare. The National Health Care Disparities Report of 2008 is a good example of this. It reports that 60% of overweight children have never been diagnosed as obese or counseled by a health care provider. Almost 40% of children have never been screened for vision problems. As you can see, these startling statistics highlight the need for QI in health care. They also emphasize how the AHRQ can promote improvement in health care.

Slide #11 – The Health Care Quality Improvement Act of 1986 is our 3^rd^ and final example of how QI has been integrated into health care policy. A major section of this act establishes the National Practitioner Data Bank. This federally sponsored database receives reports of malpractice decisions, denials of medical staff privileges, and loss of medical licenses. Reporting is required at multiple levels and by multiple organizations. Now, information for health care providers and consumers is centralized. This includes physicians who have lost their license to practice medicine, or those physicians with major malpractice settlements. This easily accessible information has offered improved access to quality health care. However, this accessibility to information has introduced new challenges. For example, there was and continues to be controversy between physician privacy and public’s right to knowledge. As evidenced by articles from the medical literature, this is a debate that continues today (slide shows Title of article: “What Every Physician Should Know About the National Practitioner Data Bank”)

Slide #12 – What does this mean for health care providers? The AMA has outlined many goals for QI and health care. Several of the key goals are listed here: 1. Enhancing the patient experience of care 2. Providing more efficient care 3. Improving the health of specified patient populations.

Slide #13 – Summary: QI in health care is important for many reasons, including that quality care is not well defined and varies widely among populations. Several programs such as The Effective Health Care Program, the AHRQ, and the Health Care QI Act of 1986 have worked to promote QI. The AMA has also outlined key goals to help direct providers in our pursuit to provide quality health care. We also know that leadership and team effectiveness are crucial components to effective and quality health care delivery. We will now talk more about each of these components.

Slide #14 – Leadership in QI- intro slide

Slide #15 – Goals: To review the potential challenges of leadership and why it is important and secondly to examine the qualities of effective leadership in QI.

Slide #16 – We all know that leadership is a key component for success, but why is it so important for QI? As the medical community continues to expand and become more complex, there is a growing pressure to perform to meet their needs. Additionally, as patients are more informed, and patient safety and satisfaction are a major focus of health care, there must be ways to see if we are indeed meeting these needs and living up to the new standard of care. Such outcome performance must be measured at multiple levels in the health care system. This is where QI comes into play and why it is so important to have effective leaders in these areas. Often times the problem is we know what needs to be done but we don’t know how to do it. Additionally, many leaders are removed from direct patient care, which makes implementing effective change difficult. Any change can be daunting so it is important to identify key areas to target.

Slide #17 – What qualities make an effective leader? Must have a will to achieve, ability to generate strong ideas for improvement, and capacity to execute them effectively. To ensure that the change is ongoing, an effective leader must also adapt, spread, and sustain ideas to meet new improvement needs. By setting a clear direction and establishing solid foundation they can generate ideas that lead to long-term changes. Additionally, the Complex Systems Theory suggests that we cannot tackle QI needs all at once, but instead should focus on critical areas or ‘leverage points’ that will lead to big improvement. Effective leaders must be able to identify these points.

Slide #18 – The Baldridge Performance Excellence Program is a public/private partnership dedicated to performance excellence and aimed at improving the competitiveness and performance of US organizations. They have developed of specific criteria that serve as a guideline for excellence. Leadership is one of the 7 criteria they outline.

Slide #19 – What does the Baldridge Program list as important qualities for effective leaders to possess in order to achieve excellence? Guide and sustain organization, communicate with workforce, participate and promote learning, foster an innovative environment, create environment of patient safety and satisfaction, promote legal and ethical behavior, encourage two-way conversation, and train future leaders. Many of their points are similar to what we discussed earlier (speaker reinforces points made earlier).

Slide #20 – Institute for Health Care Improvement (IHI) have outlined 7 key leadership leverage points as followed: 1. Establish and oversee specific system-level aims. 2. Develop an executable strategy and oversee execution. 3. Channel leadership attention to system-level improvement. 4. Make CFO quality champion- help them see how QI changes can help cut cost. 5. Put patients and families on improvement teams to keep leadership in tune with their needs. 6. Engage physicians- if not engaged they can hinder forward progress. 7. Build improvement capacity- supportive environment where team members are not afraid to question the status quo.

Slide #21 – Summary slide: Leadership in QI is important because of the growing needs of medical communities and the need for performance measures. We often know what needs to be done but effective leaders can help drive the mechanism of making changes happen. Effective leaders identify ‘leverage points’ for change and generate and sustain ideas that lead to improvement in critical areas. Effective leaders must have a will to achieve, direct interaction with all members of the team, and a creative environment for learning. Must promote patient safety and satisfaction, foster an environment of open communication.

Slide #22 – Reference slide with articles and links

Slide #23 – Intro slide to Team Effectiveness and QI

Slide #24 – Learning goals: Speaker pauses ~13 sec for learner to read them: explore the elements of an effective team, relate the concept of team effectiveness to QI and patient safety, encourage reflection on the physician’s role in a patient team, and understand the importance of effective leadership.

Slide #25 – Learning objectives: By the end of this module, you should be able to: define the characteristics of an effective team, identify why effective teamwork is important to safety and provide examples, clarify the physician’s role as a member of a patient team, and formulate a list of qualities needed for effective leadership.

Slide #26 – The importance of teamwork: first, take a moment to think about a health care team that you thought was effective. List all members of this health care team. What was it that made it effective? Speaker pauses ~73 sec. During that time on the slide an image of the famous report “To Err is Human” appears on left side of screen. To the right, several bullet points show up discussing teams that the learner can read. They read as follows: 1. Systems and processes, not individuals, make up the majority of medical errors. 2. Teams abound in the health care setting yet their members are often trained in different disciplines and come from different backgrounds. 3. Teams must learn to appreciate each other’s strengths and weaknesses.

Slide #27 – An effective team: what constitutes an effective team? A cohesive unit that works together to maximize strengths and anticipate weaknesses to achieve a common goal. A team is more than just a group of people who work side by side. A team functions as a cohesive unit in which frequent two-way communication allows everyone to participate fully and perform to their full potential. Such a group can anticipate mistakes, overcall obstacles, and navigate through difficult situations. Next point on slide is written not spoken: Team work is not task work!

Slide #28 – Teamwork and patient safety: Team work is especially important in the health care setting. People working together in teams are able to communicate more effectively and that can work to reduce medical error. In fact, according to the Joint Commission on Patient Safety, communication failures are the #1 identified cause of sentinel events, which include patient injury and even death. For example, think about a surgeon who is preparing to operate on the wrong knee of a surgical patient. A nurse in the operating room realizes the surgeon’s mistake but is too afraid to speak up about it. The surgery goes forward and the wrong knee is operated on.

Or, think about a pharmacist who asks his assistant to fill a prescription in which there is a mistake. The assistant who is afraid of their boss initially refuses to fill the prescription, saying there is a mistake on the order. The pharmacist does not listen and again instructs her to fill the prescription, again she refuses. The pharmacist is irate and orders her to fill the prescription. Finally, feeling like she has no other choice she fills the prescription. It is for 4x the correct dose.

Teamwork and patient safety cont’d: Now, take a moment to think about an example of poor communication leading to a negative outcome. Perhaps you yourself were involved in a situation where you were hesitant to speak up and did not feel like a valued member of the team (speaker pauses several seconds for reflection). However, it is important to note that while ineffective teamwork can lead to medical error, on the other hand good teamwork can help improve patient safety.

Teamwork and patient safety cont’d: Take for example congenital heart surgery at Texas Children’s Hospital. The institution of case conferences amongst the cardiovascular surgeons, cardiologists, and anesthesiologists, along with creation of multidisciplinary team has greatly reduced post-operative mortality and improved the overall quality of health care delivered to children born with congenital heart disease.

You do not have to discuss all these examples; you can choose one or two to discuss. We recommend also asking the learner do they have any examples of team work and patient safety.

Slide #29 – Your role in a team: Every person in health care organization will at some point be a member of a patient care team no matter what their role. Thus, we all must learn to communicate effectively and value the contributions of other team members in order to maximize patient safety. The processes of health care are too complex to be safely carried out by individuals who try hard. You must be intentional and systematic about your approach to teamwork.

Slide #30 – Effective Team Members: Speaker asks learner to review qualities listed (pauses ~6 sec). Effective teams should have a shared vision and clearly delineated roles and responsibilities for their members. An effective team member is someone who knows their role on the team. They can predict the needs of other team members, provide quality information and feedback to team as a whole, and engage in higher level decision making. They should also be able to manage conflict skillfully to improve the overall functioning of the team.

Slide #31 – Leadership and Teamwork: Finally, it is very likely that you may find yourself in a team leadership position at some point in your career. The role of the team leader is to set the tone for collaboration and ensure that all members of the team feel valued and will work together as a cohesive group rather than a collection of individual experts. A team leader’s responsibilities include engaging all members of the team, using first names to bring everyone into an open playing field, involving team members in a two-way conversation. Team leaders should encourage feedback and respond to suggestions in a timely manner. Finally, a team leader should respect and value every team member and his or her input.

Slide #32 – Summary slide: Now think back to the team you recalled at the beginning of the module. Was it truly an effective team? Are there any improvements that could have been made to improve team effectiveness? Was there a team leader and if so did they fulfill the qualities of good leadership. I hope this module has emphasized the importance of team work. Systems are largely responsible for medical errors, not individuals. Teams represents a large part of health care organizations and as such must work together to maximize their strengths and anticipate weaknesses. Effective team work can improve patient safety. An effective leader can cultivate a good team, however we must all be intentional in our approach to teamwork and patient care!

Slide #33 – References: list of 5 references including articles and websites.
