# Supplementary material for: Engaging Learners Through Modules in Quality Improvement and Patient Safety
Source: MedEdPORTAL. 2016 Oct 13;12:10482. doi: 10.15766/mep_2374-8265.10482 (PMC6440404; doi:10.15766/mep_2374-8265.10482)
Supplement: Supplementary file 1 — A. Instructor's Guide.docx B. PowerPoint Talking Points.docx C. Knowledge Survey.docx D. Attitude Survey Questions.docx E. Fundamentals of QI.pptx F. Fundamentals of Patient Safety.ppt G. Evidence-Based Practice and QI Improvement Research.pptx H. QI and PS Potpourri.pptx [file mep-12-10482-s001.zip › C. Knowledge Survey.docx]

**Appendix C. Knowledge Test Questions and Answers**

The knowledge questions are listed below and the answers for each questions are underlined.

1. The practice of evidence based medicine means utilizing clinically relevant research to make decisions about the care of individual patients, rather than using the judgment that individual clinicians acquire through clinical experience and clinical practice.

1. True
2. False
3. Which one of the following is NOT a goal of the American Medical Association?

Select one:

1. Enhancing the patient experience of care
2. Providing more efficient care by reducing or controlling the per capita cost of care for
3. Improving the health of specified patient populations
4. Limiting availability of health care to poor and underserved populations
5. Quality improvement studies should be held to the same rigorous standards that are used to evaluate clinical studies.
6. True
7. False
8. Which of the following do effective leaders do?
9. Know the correct answer in any situation
10. b. Have the most experience in the team
11. c. Seek input from all members of the team
12. d. Ensure that they have the final say in all team decisions
13. A study of pediatric thoracic surgery patients compared patient outcomes at multiple points in time between a baseline period and a post-intervention period, after the implementation of an evidence-based clinical guideline for postoperative management. The authors reported significant improvements in outcomes in the post-intervention period vs. the baseline period. However, re-analysis revealed a significant pre-intervention trend, and time-series regression techniques demonstrated no significant differences after the intervention. What design was used for this study?

a. Randomized controlled trial

b. Stepped-wedge decision

c. Time series design

d. Controlled before-after study

1. Which of the following is NOT a critical appraisal tool you can use to evaluate studies or evidence-based guidelines?
2. PRISMA
3. CONSORT
4. DELTA
5. AGREE
6. Of the following, which supports the importance of quality improvement in health care?
7. Evidence indicates that the quality of health care that Americans receive is not ideal.
8. What constitutes good quality healthcare is not clearly defined
9. Quality varies widely
10. Quality of Health Care varies across populations
11. All of the above
12. Which of the following is not a challenge in Quality Improvement (more than one answer may be selected)?
13. Growing pressure to perform
14. Many leaders are removed from patient care, don’t have personal connection
15. Ability to effectively execute ideas
16. No direct connection with people they are asking changes of
17. Don’t know what to implement strategic small changes to make larger impact
18. Quality Improvement and idea for change can be very daunting
19. Eagerness of front-line workers to support
20. You cannot make an evidence-based recommendation on a clinical topic if there are no large randomized studies addressing the topic.
21. True
22. False
23. The best way to ensure safe, high quality healthcare is to be an individual expert in your field.
24. True
25. False
26. Which of the following is not a quality of an effective leader?
27. Will to achieve
28. Generate/find ideas
29. Discourage members of organization to question the status quo
30. Execute and sustain ideas
31. Connect on personal level with patients and members of team
32. The following are part of the 6 Aims for Improvement EXCEPT:
33. Effective
34. Efficient
35. Evidence-based
36. Equitable
37. The IHI Model for Improvement suggests creating an aim, defining measures, and consider changes prior to beginning a PDSA cycle.
38. True
39. False
40. Is the following a SMART aim statement?
    *By June 2013, 50% of pediatric interns at Baylor College of Medicine will be trained in the PHM Quality Improvement and Patient Safety Curriculum.*
41. Yes
42. No
43. Which of the following is an example of a medical error?
44. Medication Errors
45. Errors in Handoff
46. Diagnostic Errors
47. All of the above
48. Match the following diagnostic errors with their definitions. We have paired the bias with the correct answer

| Anchoring bias | Relying on first impression |
| --- | --- |
| Availability bias | Assuming first possibility that comes to mind |
| Confirmation bias | Focusing on evidence that supports rather than refutes; |
| Blind obedience | Deferring to authority/hierarchical superiors or technology |
| Premature closure | Holding a narrow-minded belief in a single idea |

1. Disclosure of a medical error has been shown to decrease the risk of lawsuit.
2. True
3. False
