# Supplementary material for: Engaging Learners Through Modules in Quality Improvement and Patient Safety
Source: MedEdPORTAL. 2016 Oct 13;12:10482. doi: 10.15766/mep_2374-8265.10482 (PMC6440404; doi:10.15766/mep_2374-8265.10482)
Supplement: Supplementary file 1 — A. Instructor's Guide.docx B. PowerPoint Talking Points.docx C. Knowledge Survey.docx D. Attitude Survey Questions.docx E. Fundamentals of QI.pptx F. Fundamentals of Patient Safety.ppt G. Evidence-Based Practice and QI Improvement Research.pptx H. QI and PS Potpourri.pptx [file mep-12-10482-s001.zip › D. Attitude Survey Questions.docx]

**Appendix D. Attitude Survey**

Please answer the following: BEFORE and AFTER your participation in these modules, how comfortable are you in your current skills with the following aspects of quality assessment and improvement? (1) Not at all, (2) Slightly, (3) Moderately, and (4) Extremely

1. Writing a clear problem statement (goal, aim).
2. Applying the best professional knowledge.
3. Using measurement to improve your skills.
4. Studying the process.
5. Making changes in a system.
6. Identifying whether a change leads to an improvement in your skills.
7. Using small cycles of change.
8. Identifying best practices and comparing these to your local practice/skills.
9. Implementing a structured plan to test a change.
10. Using the PDSA model as a systematic framework for trial and learning.
11. Identifying how data is linked to specific processes.
12. Building your next improvement upon prior success or failure.
