# Supplementary material for: Engaging Learners Through Modules in Quality Improvement and Patient Safety
Source: MedEdPORTAL. 2016 Oct 13;12:10482. doi: 10.15766/mep_2374-8265.10482 (PMC6440404; doi:10.15766/mep_2374-8265.10482)
Supplement: Supplementary file 1 — A. Instructor's Guide.docx B. PowerPoint Talking Points.docx C. Knowledge Survey.docx D. Attitude Survey Questions.docx E. Fundamentals of QI.pptx F. Fundamentals of Patient Safety.ppt G. Evidence-Based Practice and QI Improvement Research.pptx H. QI and PS Potpourri.pptx [file mep-12-10482-s001.zip › E. Fundamentals of QI.pptx]

## Slide 1
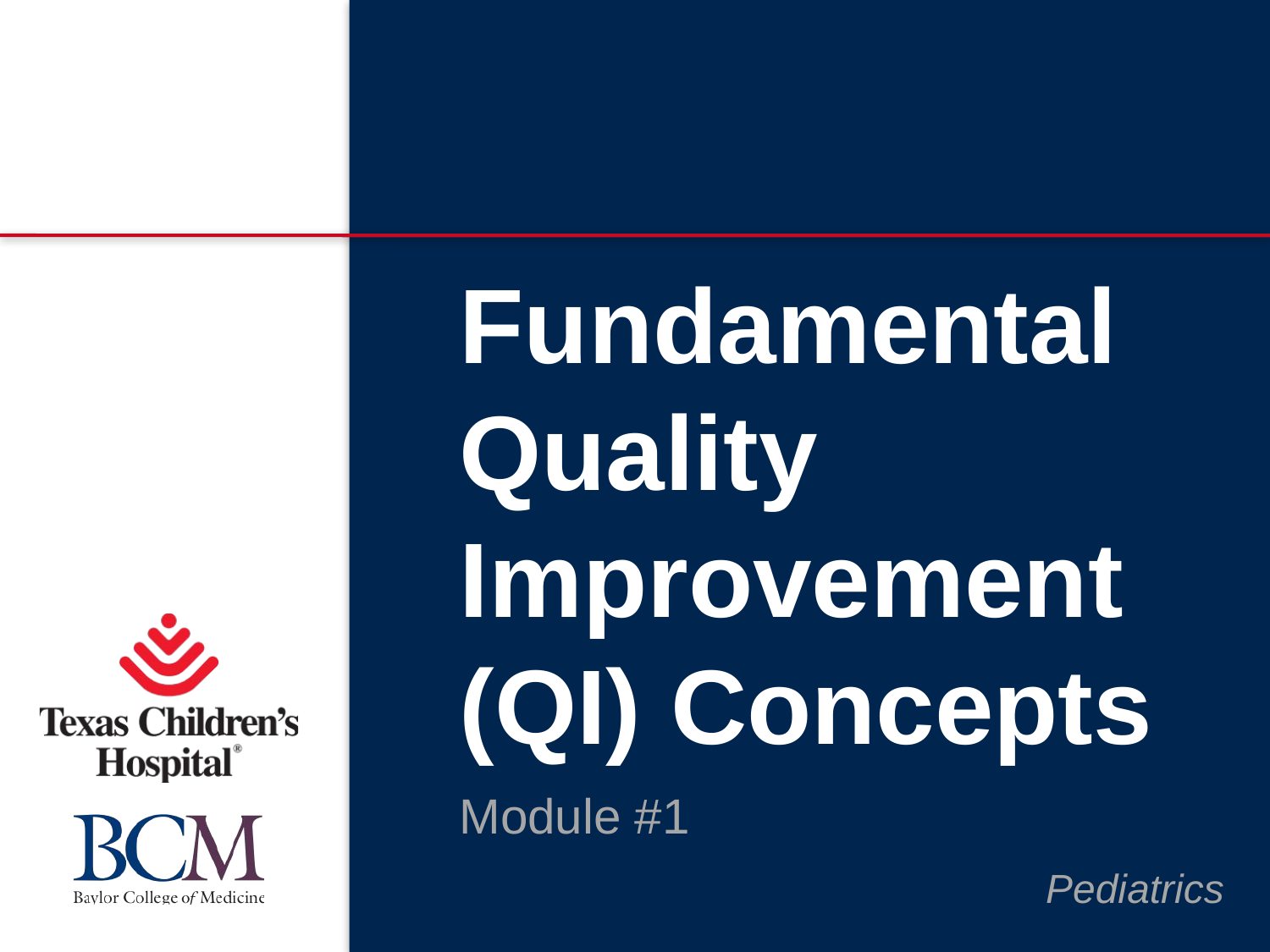

# Fundamental Quality Improvement (QI) Concepts
Module #1

## Slide 2
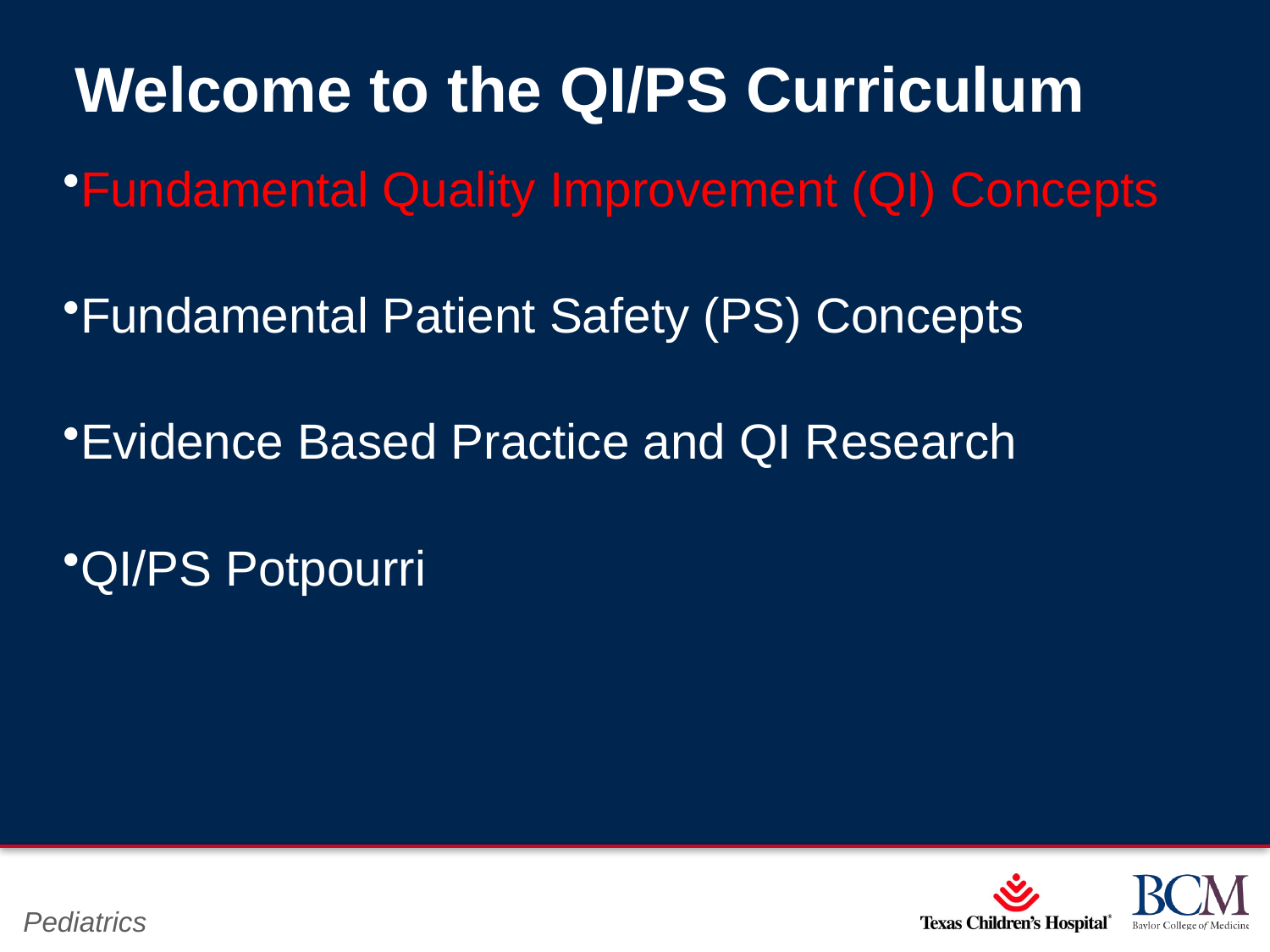

# Welcome to the QI/PS Curriculum
Fundamental Quality Improvement (QI) Concepts
Fundamental Patient Safety (PS) Concepts
Evidence Based Practice and QI Research
QI/PS Potpourri

## Slide 3
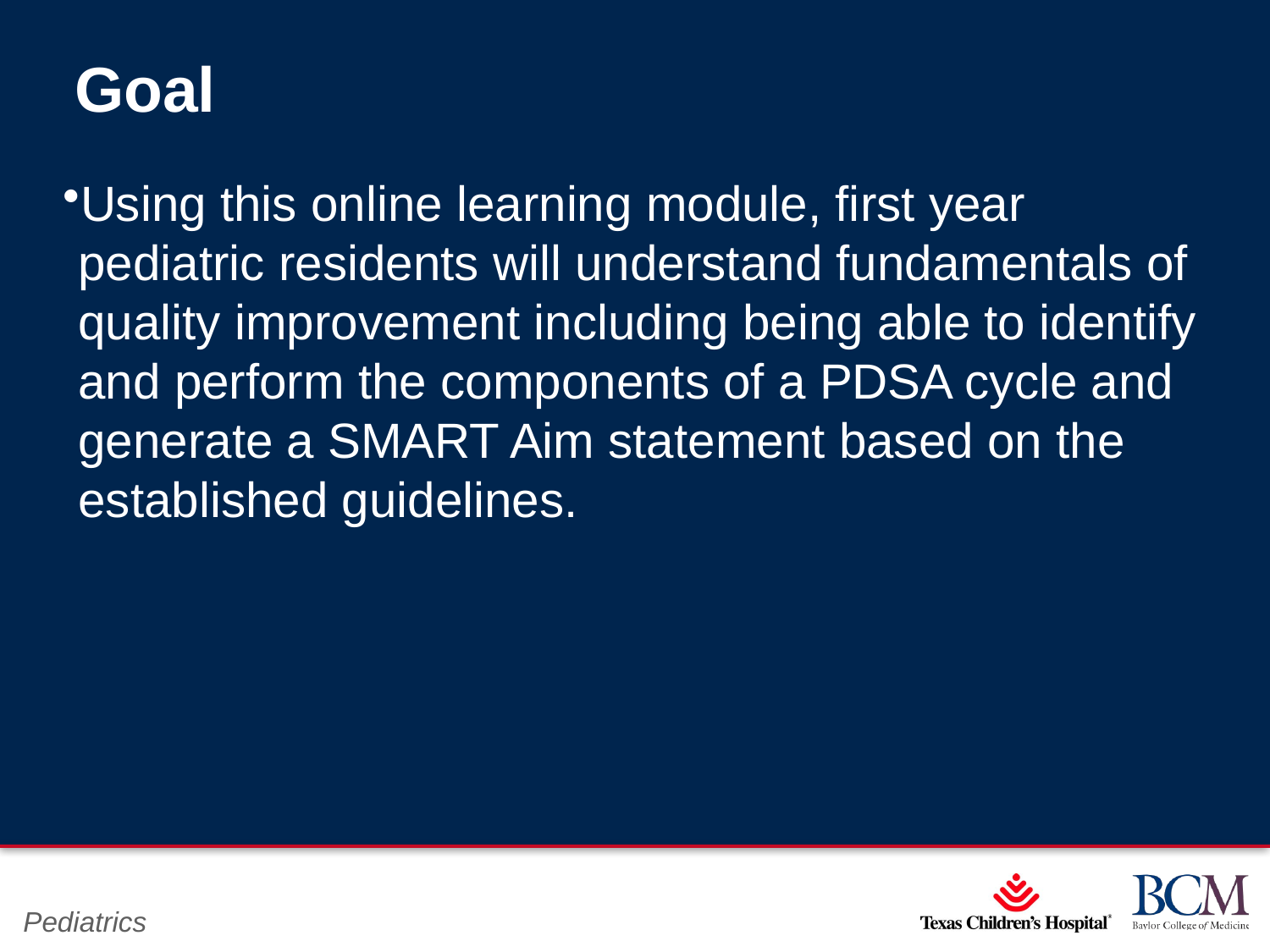

# Goal
Using this online learning module, first year pediatric residents will understand fundamentals of quality improvement including being able to identify and perform the components of a PDSA cycle and generate a SMART Aim statement based on the established guidelines.

## Slide 4
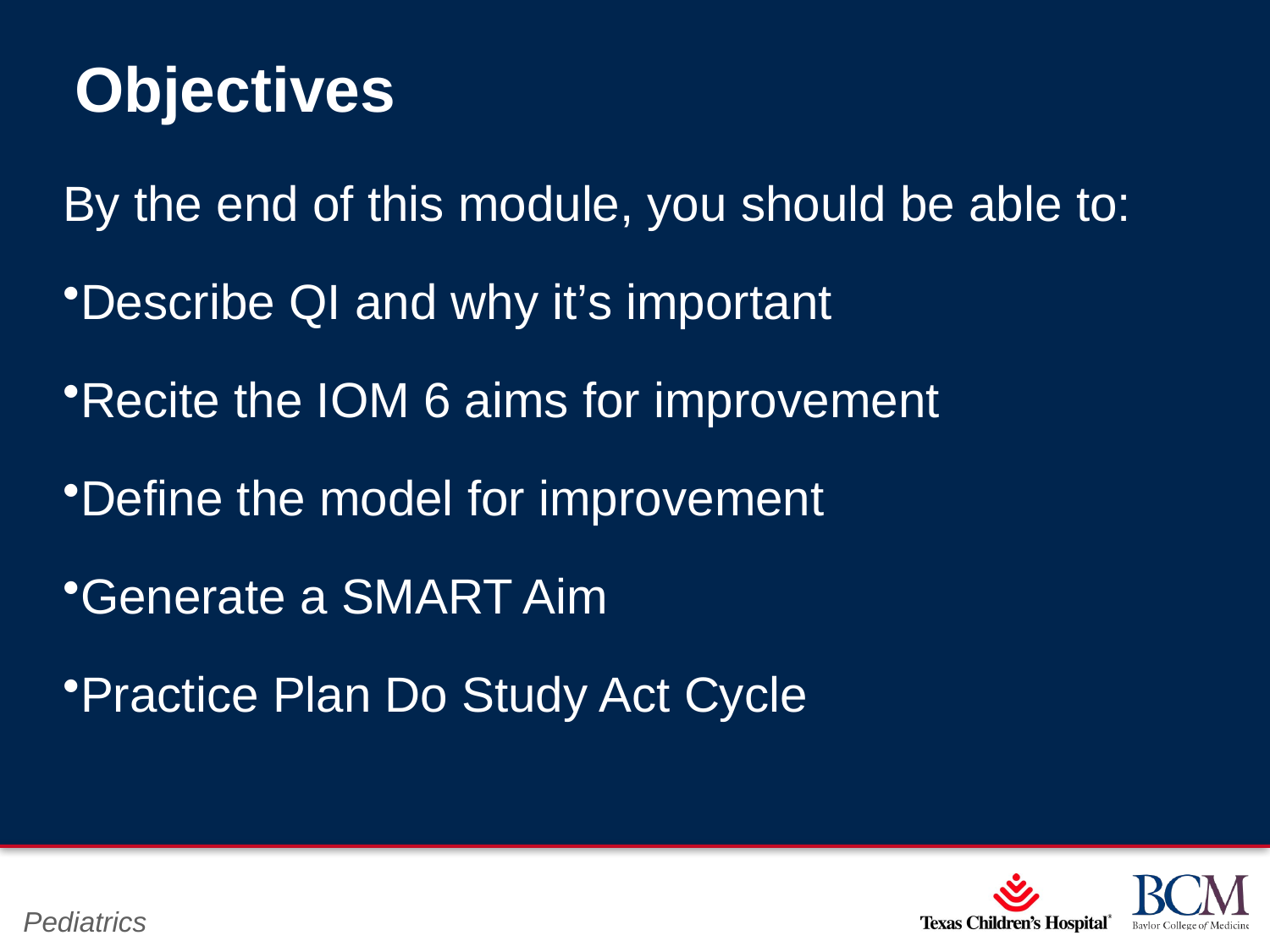

# Objectives
By the end of this module, you should be able to:
Describe QI and why it’s important
Recite the IOM 6 aims for improvement
Define the model for improvement
Generate a SMART Aim
Practice Plan Do Study Act Cycle

## Slide 5
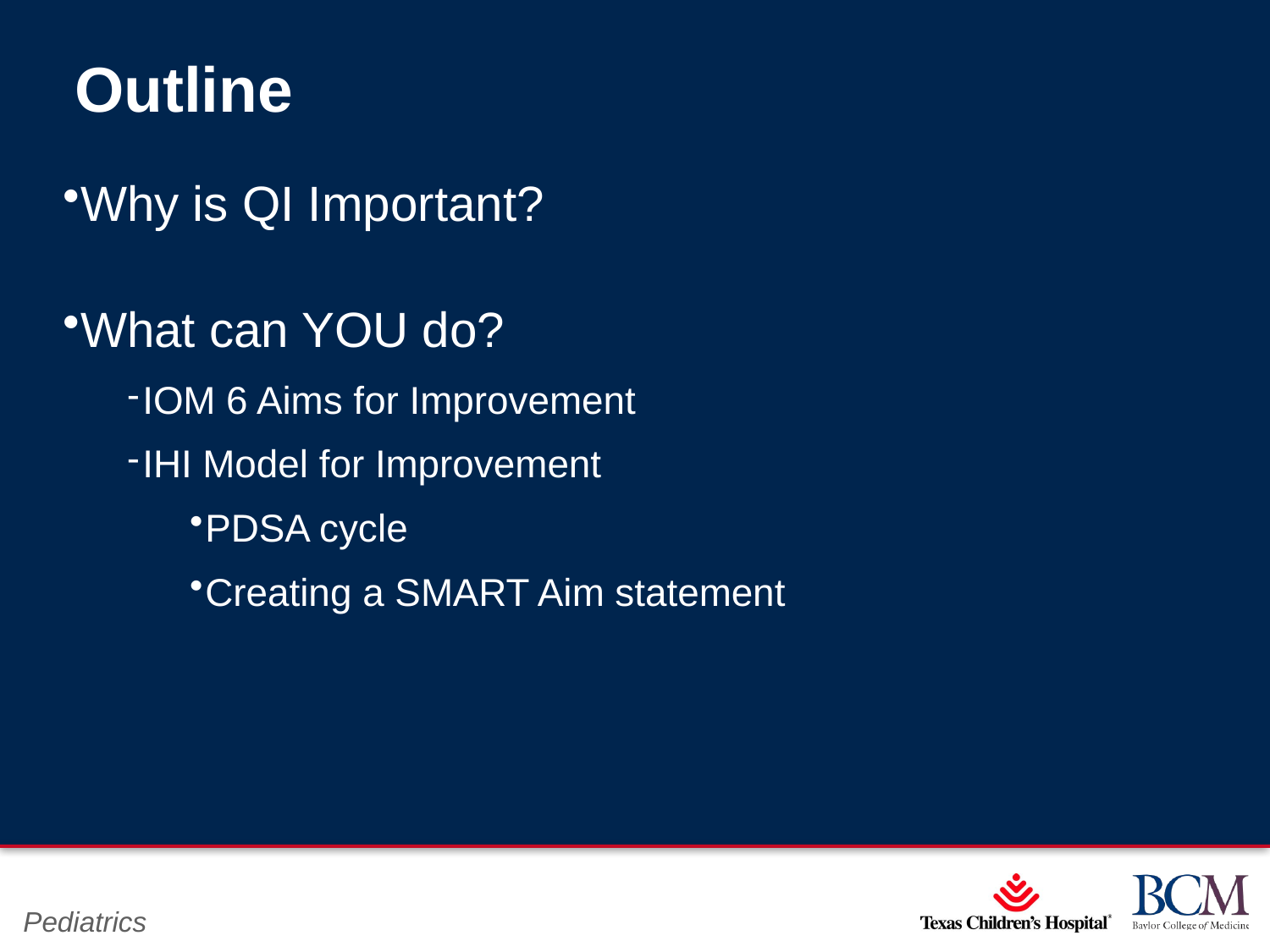

# Outline
Why is QI Important?
What can YOU do?
IOM 6 Aims for Improvement
IHI Model for Improvement
PDSA cycle
Creating a SMART Aim statement

## Slide 6
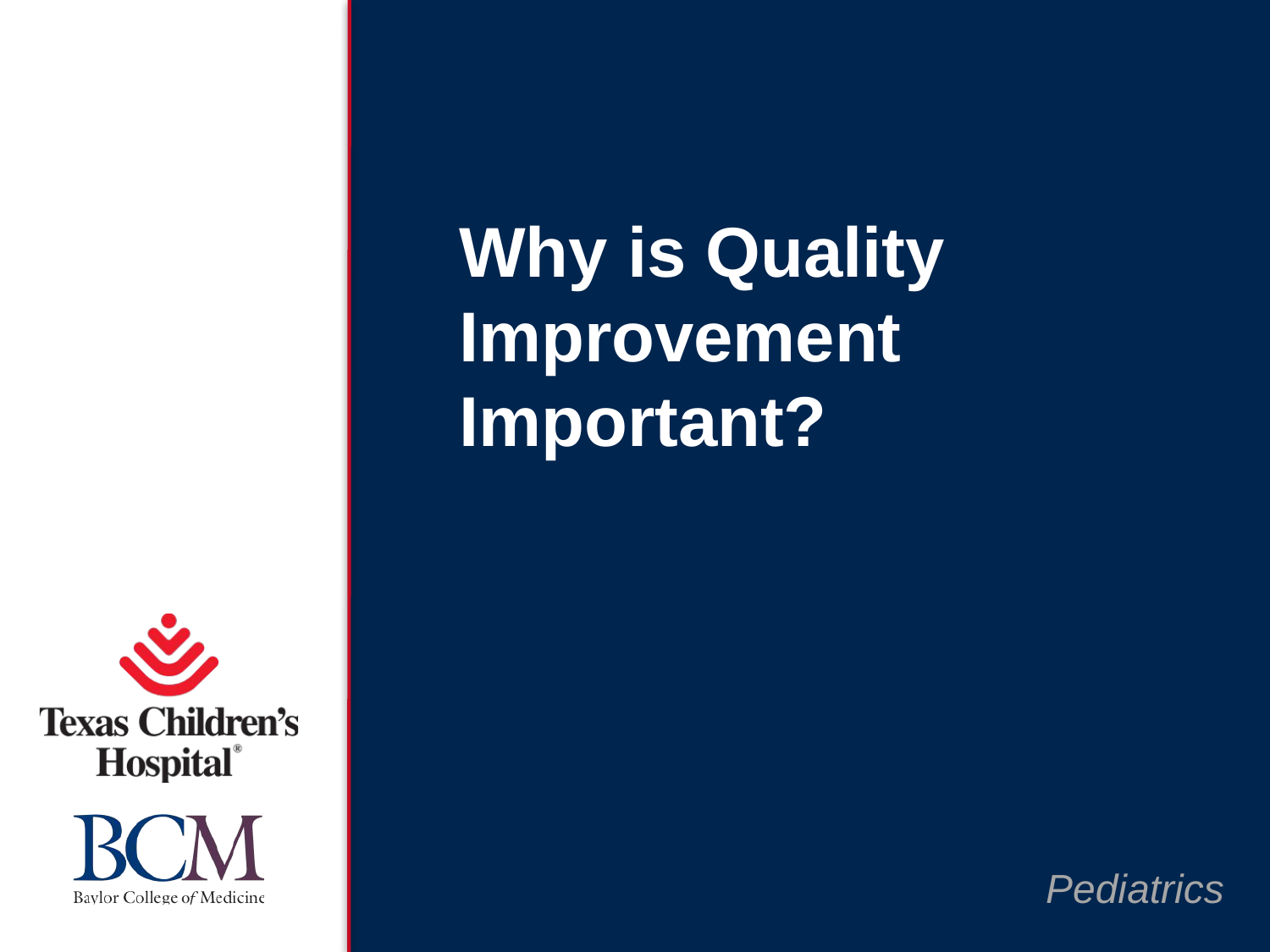

# Why is Quality Improvement Important?

## Slide 7
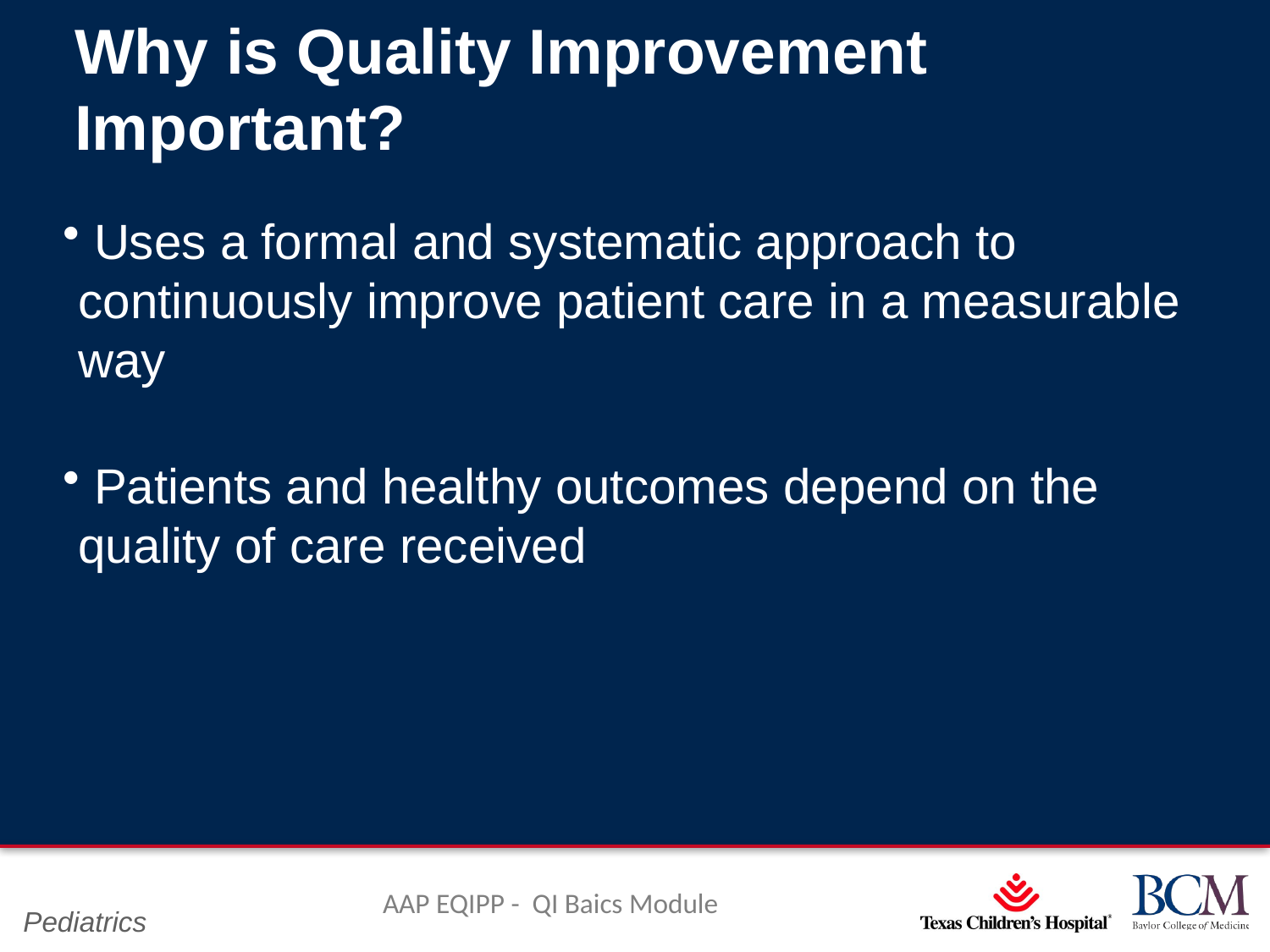

# Why is Quality Improvement Important?
 Uses a formal and systematic approach to continuously improve patient care in a measurable way
 Patients and healthy outcomes depend on the quality of care received
AAP EQIPP - QI Baics Module

## Slide 8
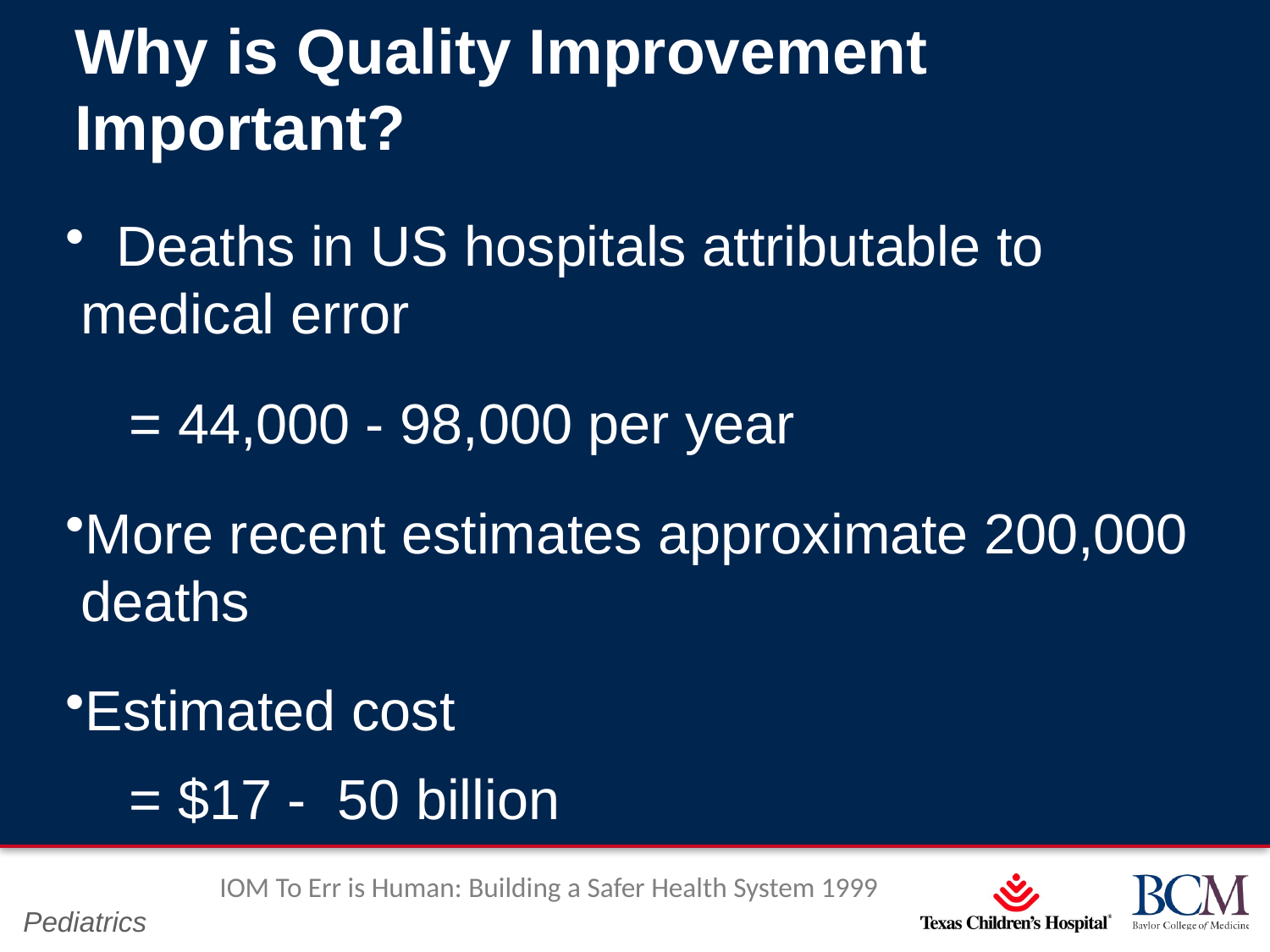

# Why is Quality Improvement Important?
 Deaths in US hospitals attributable to medical error
= 44,000 - 98,000 per year
More recent estimates approximate 200,000 deaths
Estimated cost
= $17 - 50 billion
IOM To Err is Human: Building a Safer Health System 1999

## Slide 9
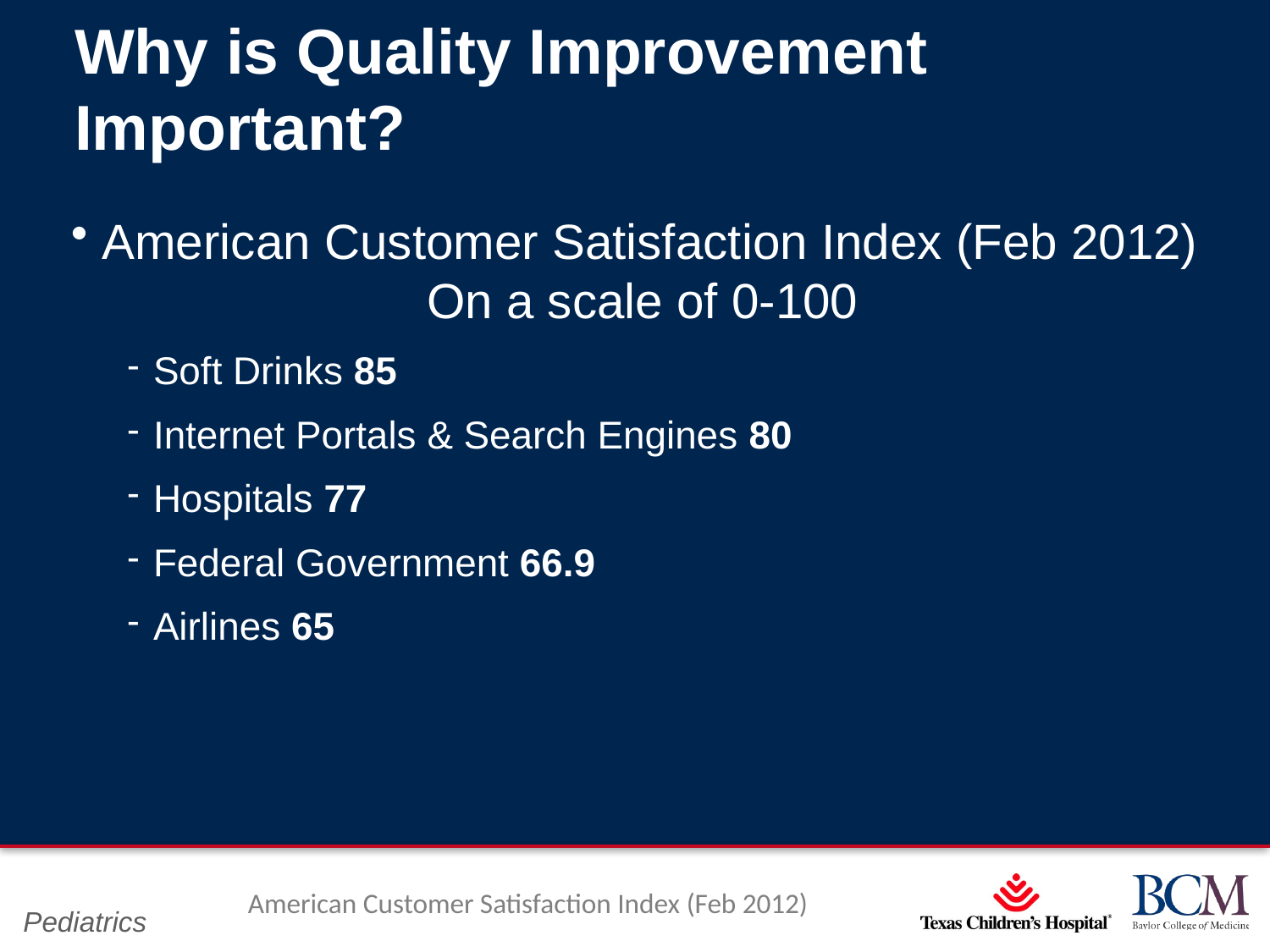

# Why is Quality Improvement Important?
 American Customer Satisfaction Index (Feb 2012) On a scale of 0-100
 Soft Drinks 85
 Internet Portals & Search Engines 80
 Hospitals 77
 Federal Government 66.9
 Airlines 65
American Customer Satisfaction Index (Feb 2012)

## Slide 10
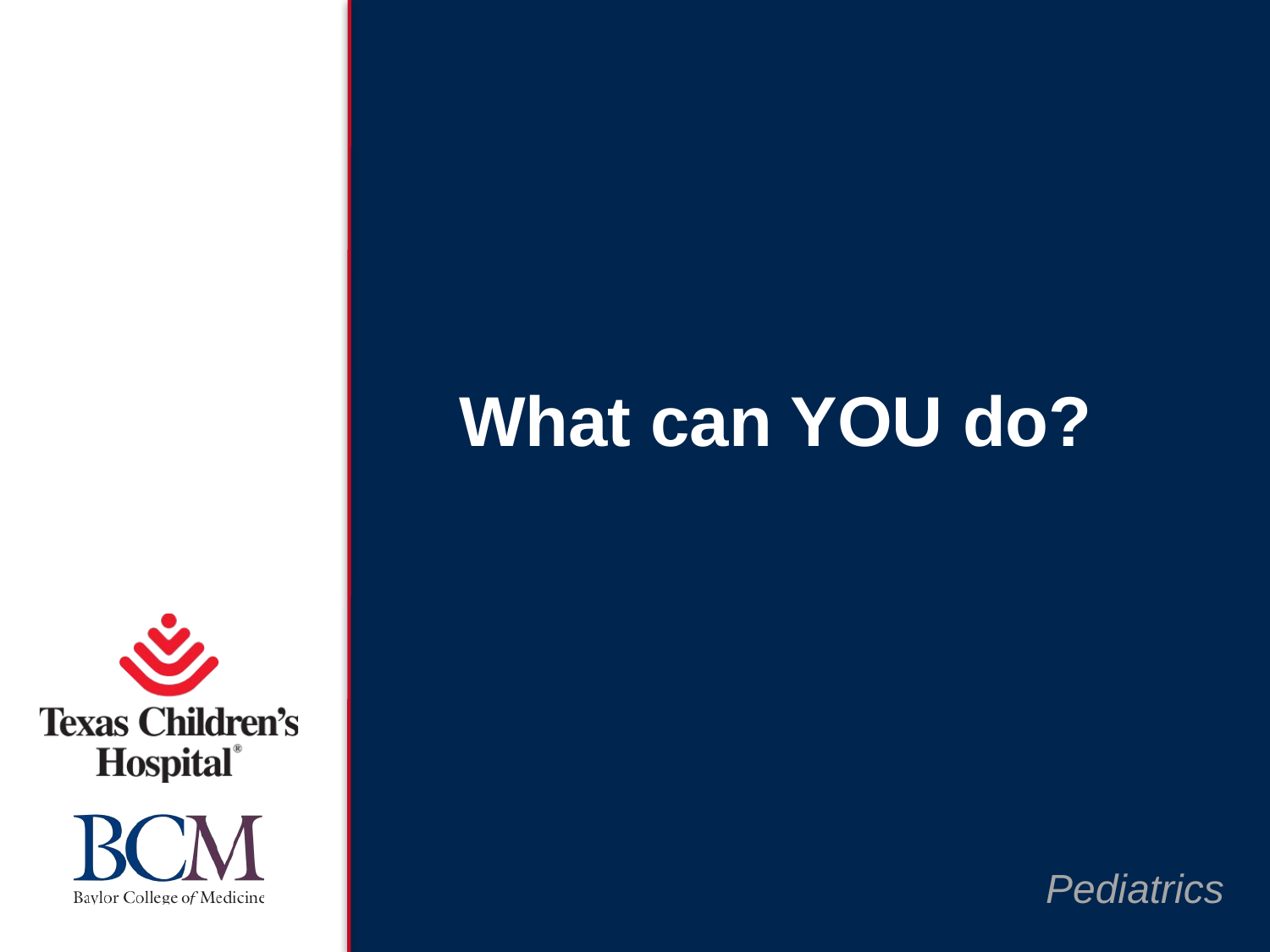

# What can YOU do?

## Slide 11
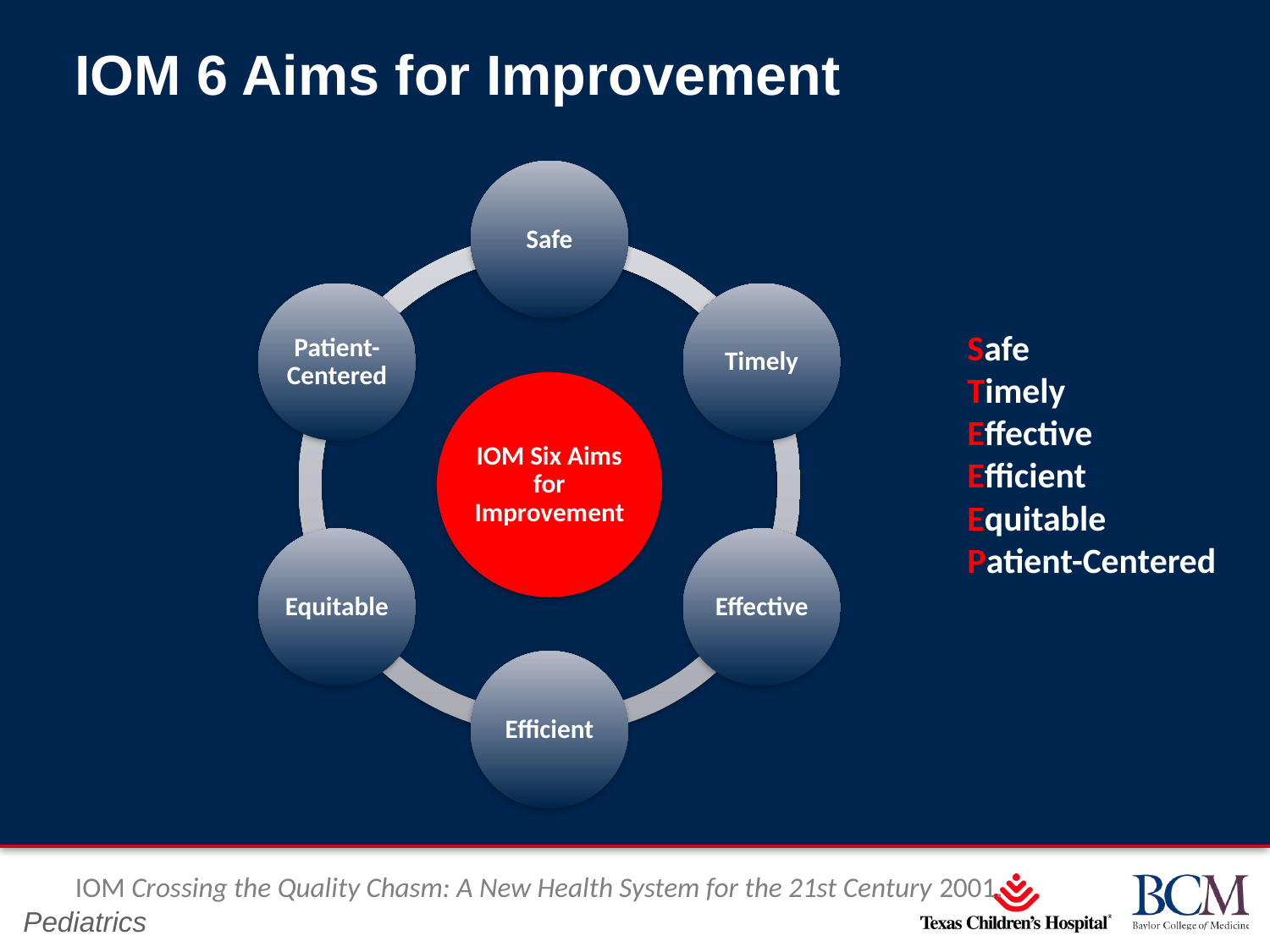

# IOM 6 Aims for Improvement
Safe
Timely
Effective
Efficient
Equitable
Patient-Centered
IOM Crossing the Quality Chasm: A New Health System for the 21st Century 2001.

## Slide 12
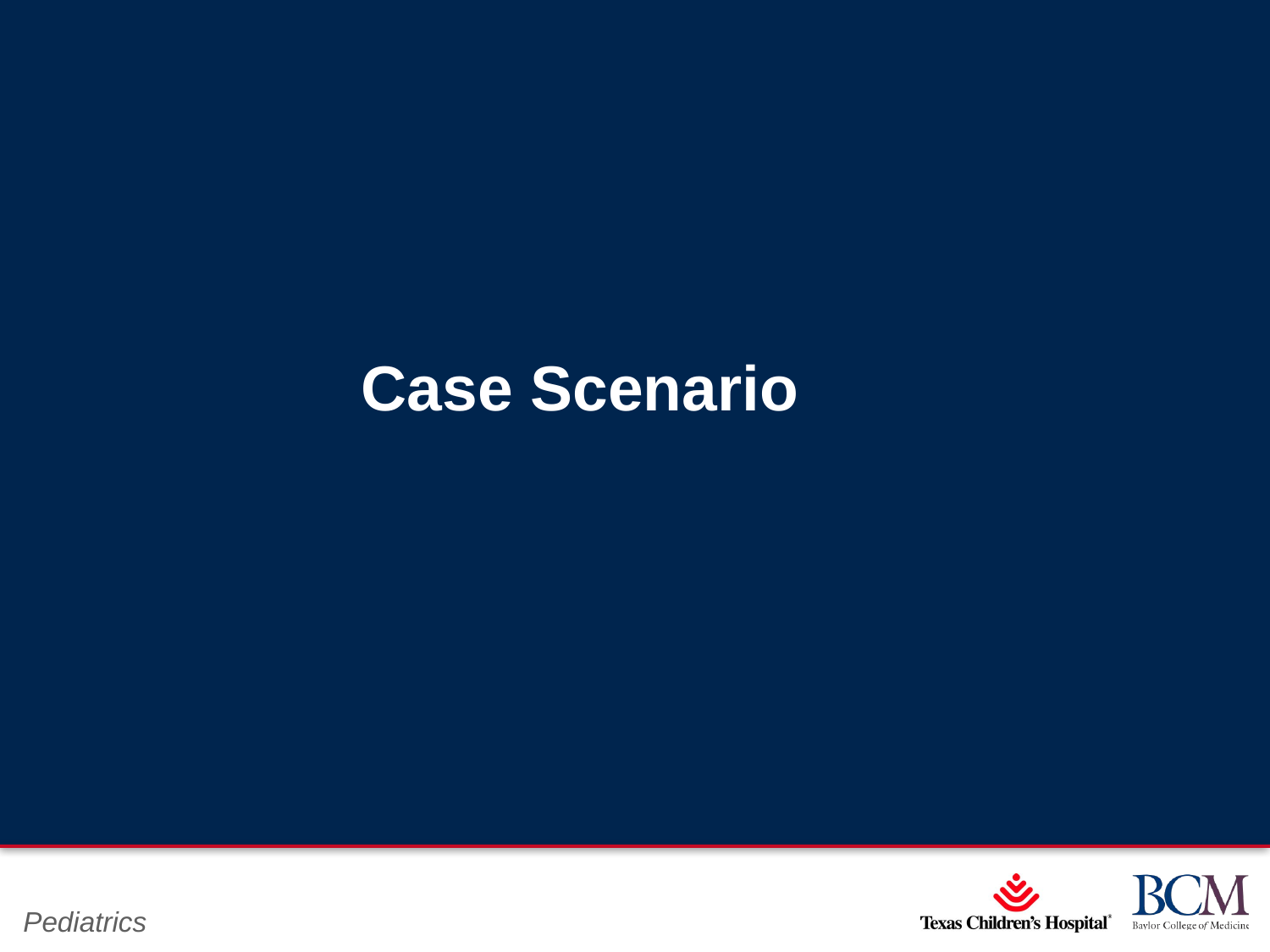

# Case Scenario

## Slide 13
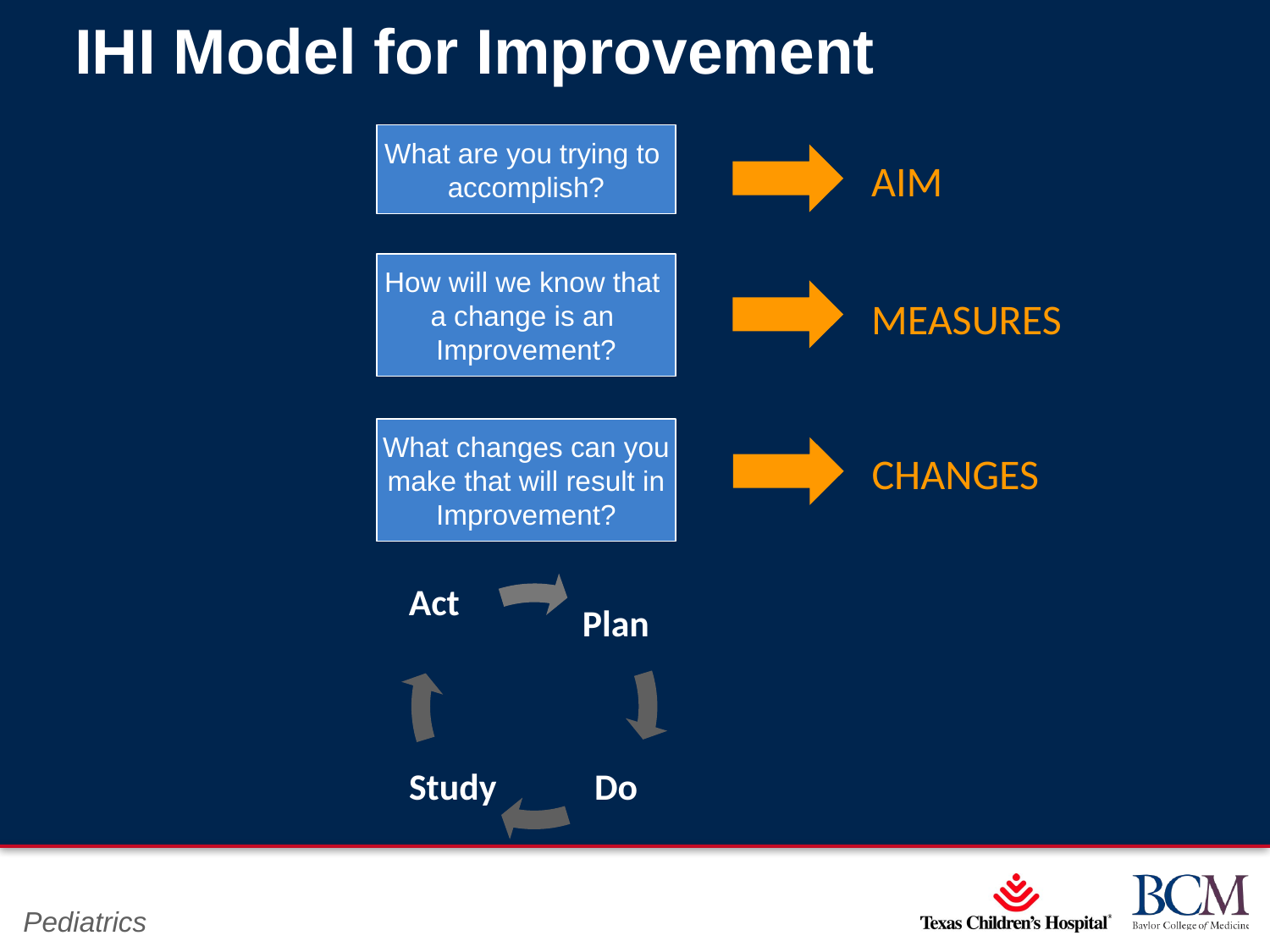

# IHI Model for Improvement
What are you trying to
accomplish?
AIM
How will we know that
a change is an
Improvement?
MEASURES
What changes can you
make that will result in
Improvement?
CHANGES

## Slide 14
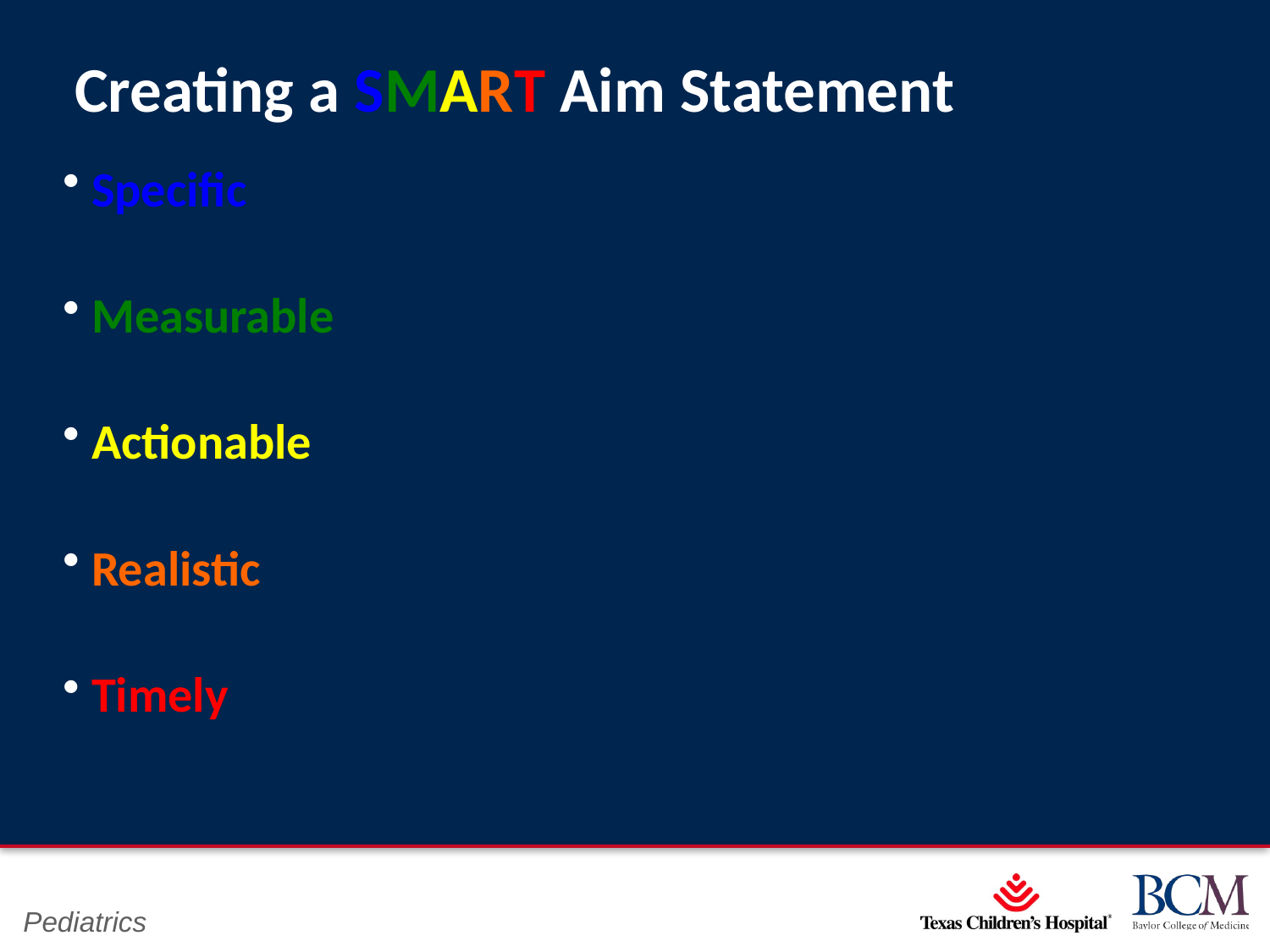

# Creating a SMART Aim Statement
 Specific
 Measurable
 Actionable
 Realistic
 Timely

## Slide 15
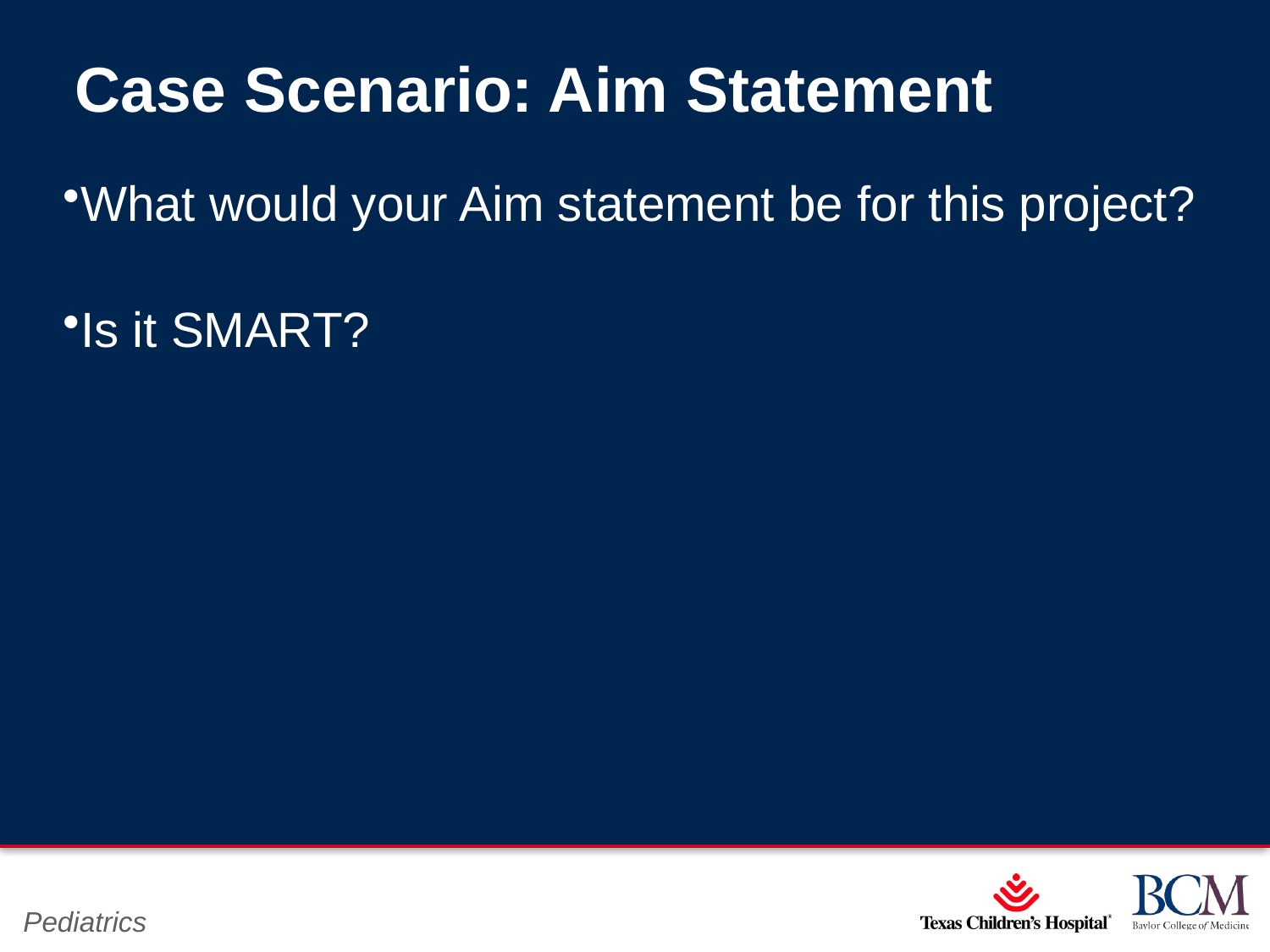

# Case Scenario: Aim Statement
What would your Aim statement be for this project?
Is it SMART?

## Slide 16
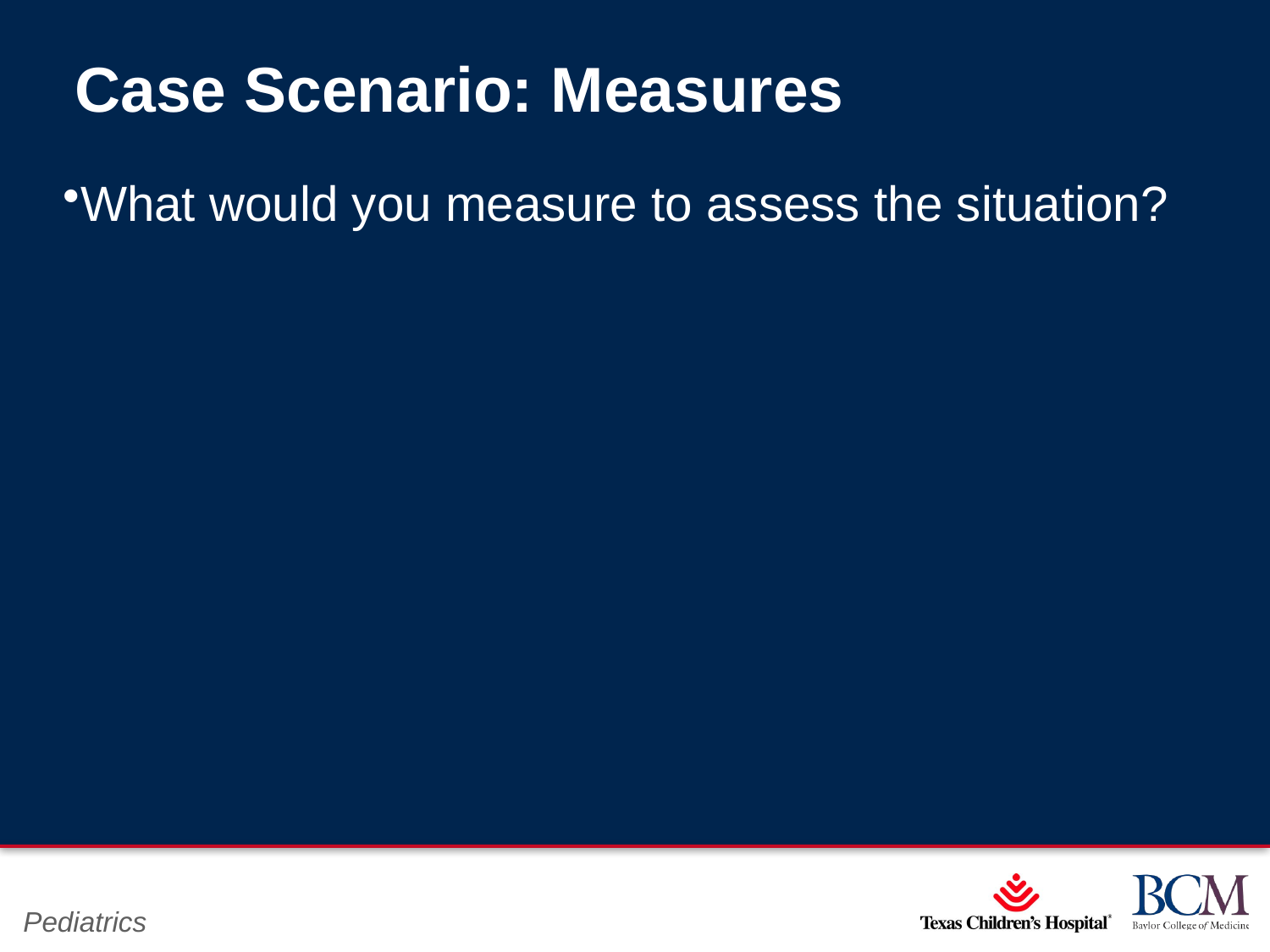

# Case Scenario: Measures
What would you measure to assess the situation?

## Slide 17
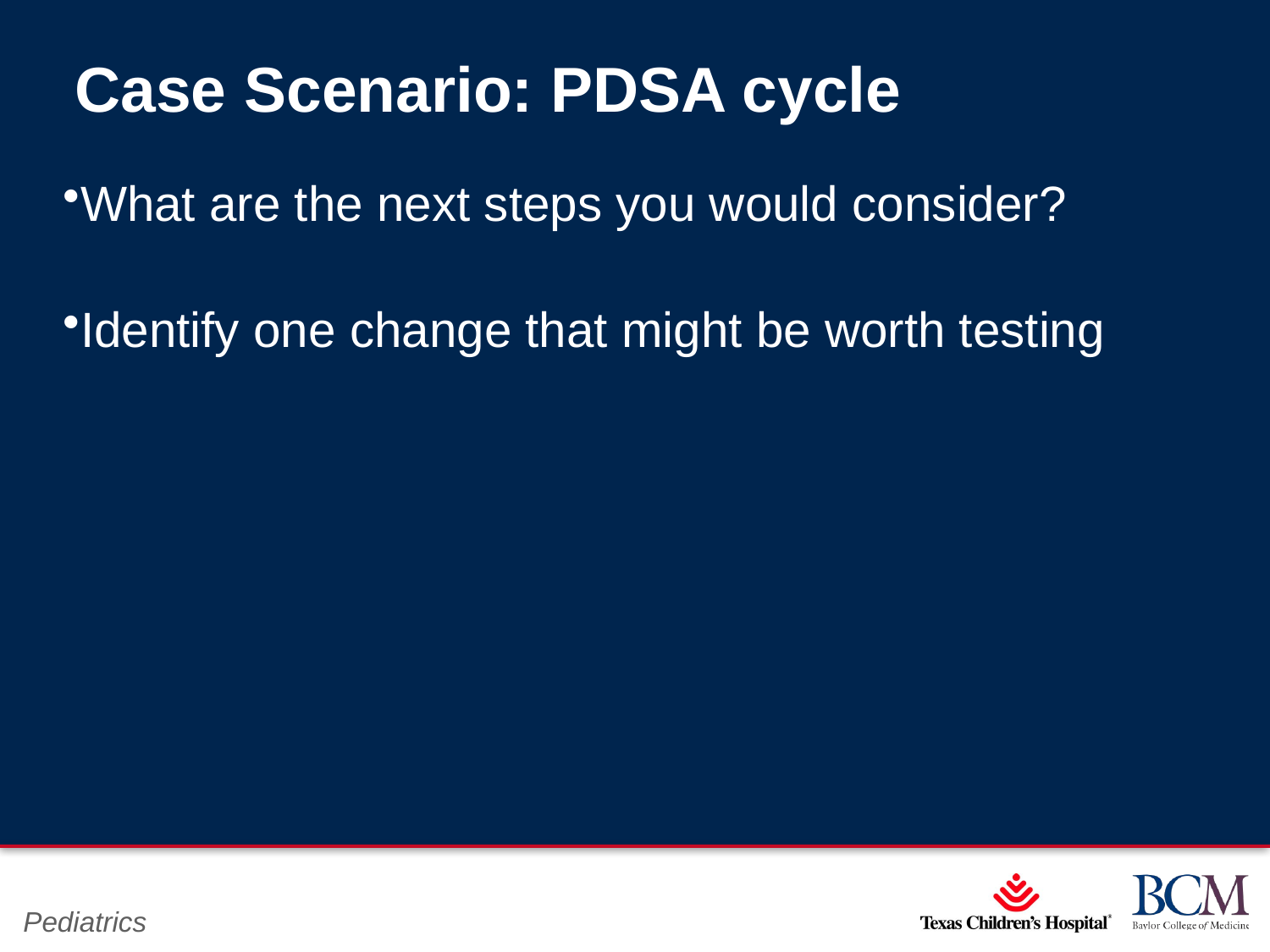

# Case Scenario: PDSA cycle
What are the next steps you would consider?
Identify one change that might be worth testing

## Slide 18
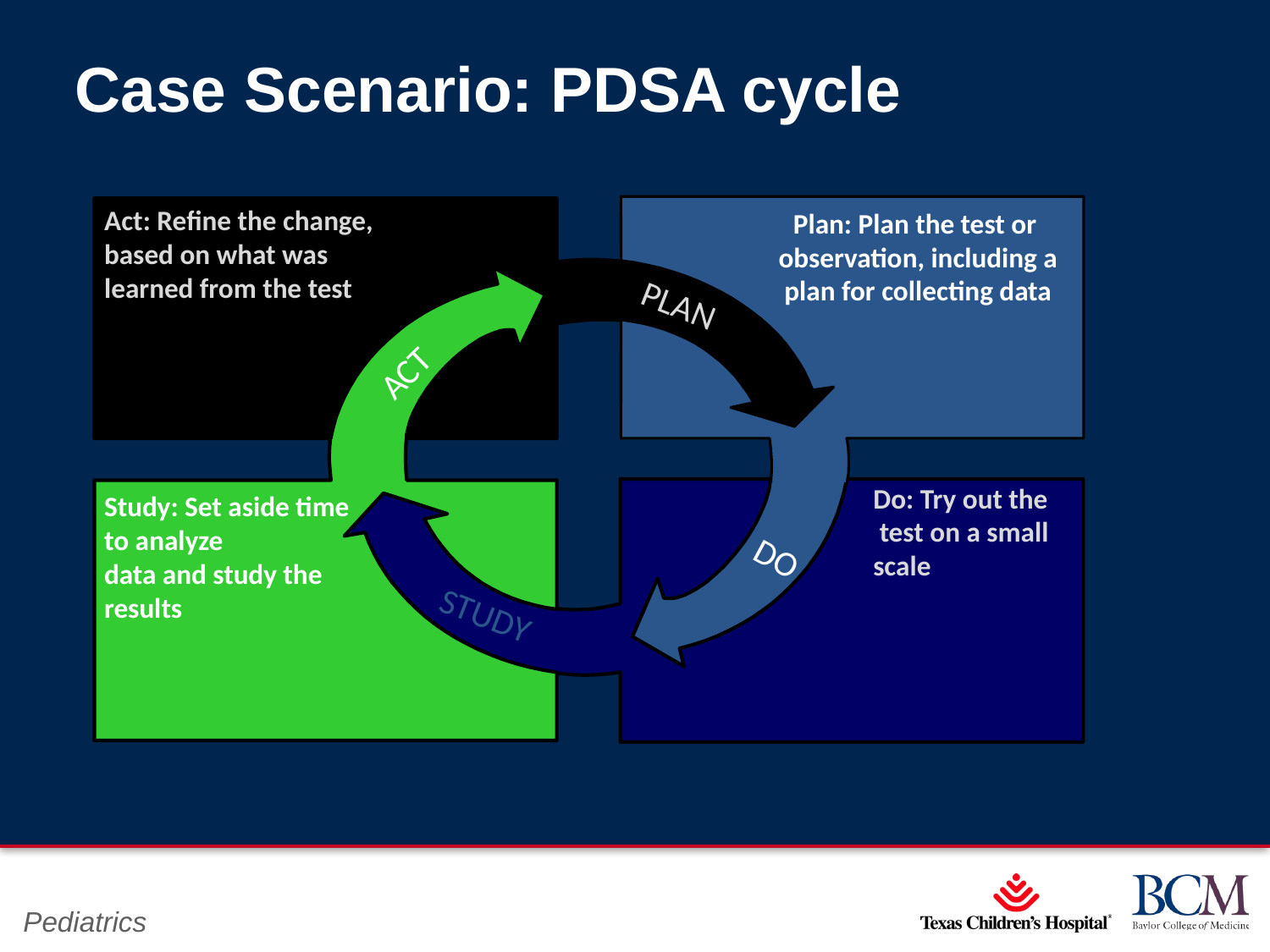

# Case Scenario: PDSA cycle
Act: Refine the change, based on what was learned from the test
Plan: Plan the test or
observation, including a
plan for collecting data
PLAN
ACT
Do: Try out the
 test on a small
scale
Study: Set aside time to analyze
data and study the results
DO
STUDY

## Slide 19
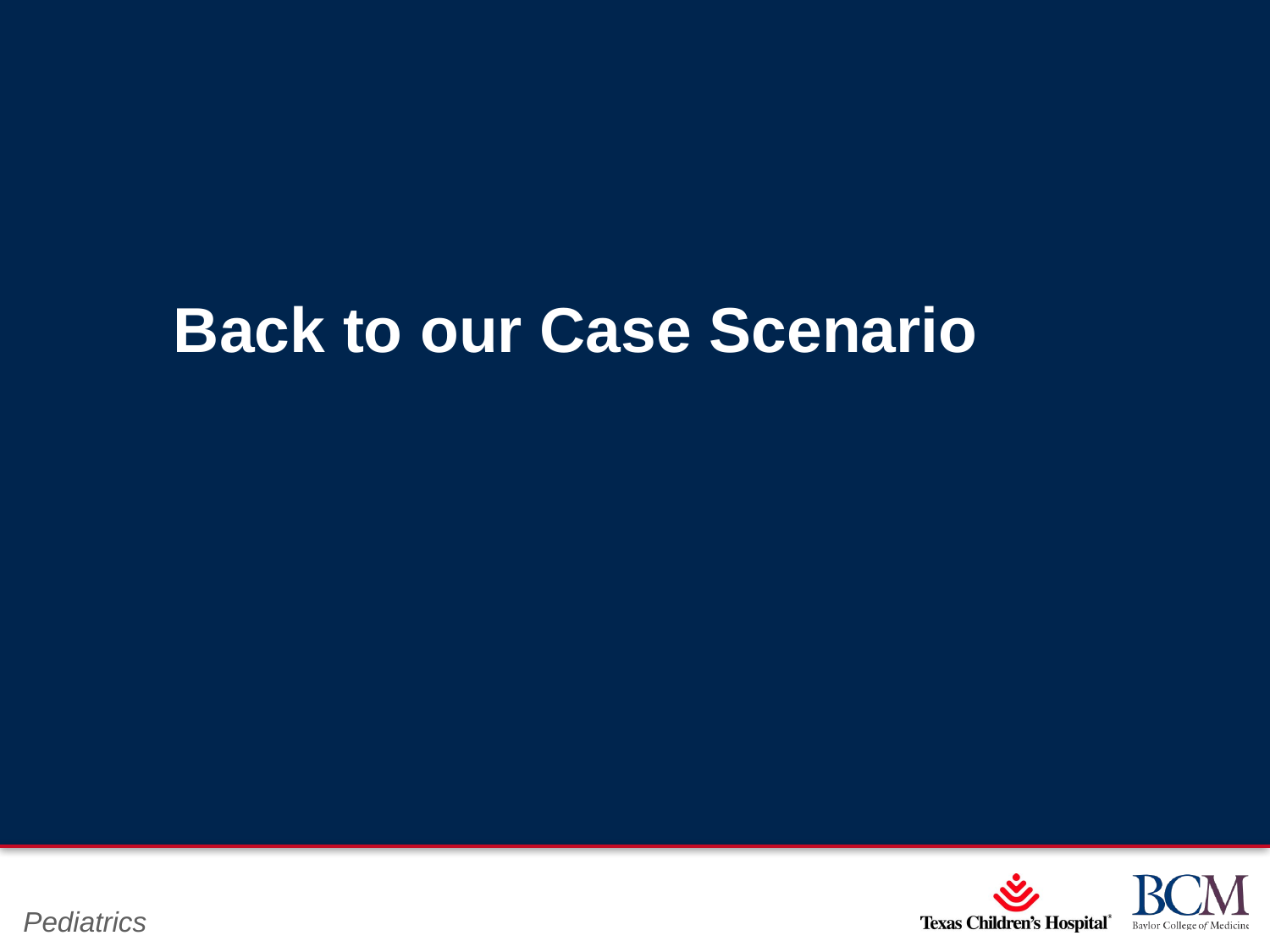

# Back to our Case Scenario

## Slide 20
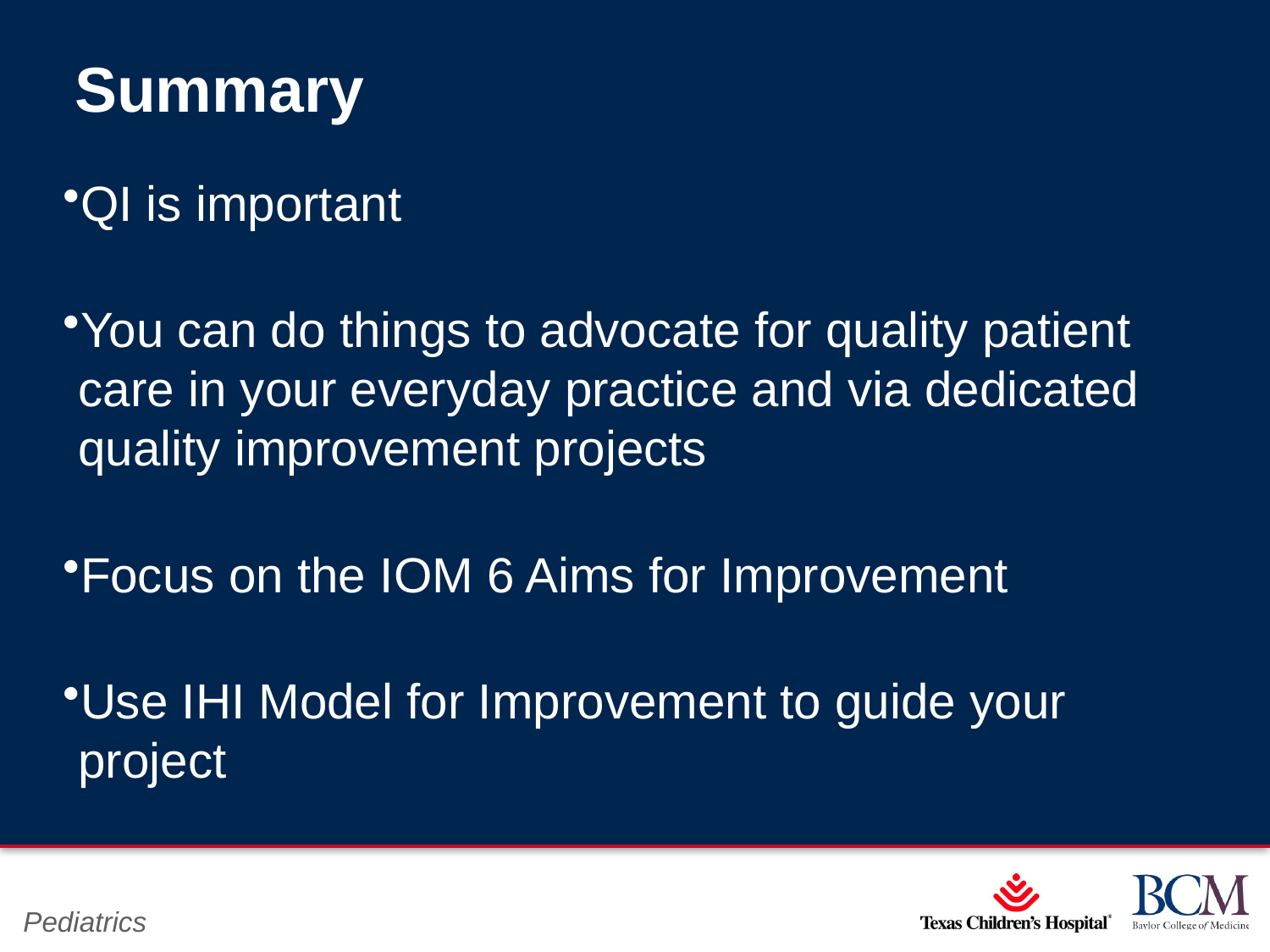

# Summary
QI is important
You can do things to advocate for quality patient care in your everyday practice and via dedicated quality improvement projects
Focus on the IOM 6 Aims for Improvement
Use IHI Model for Improvement to guide your project

## Slide 21
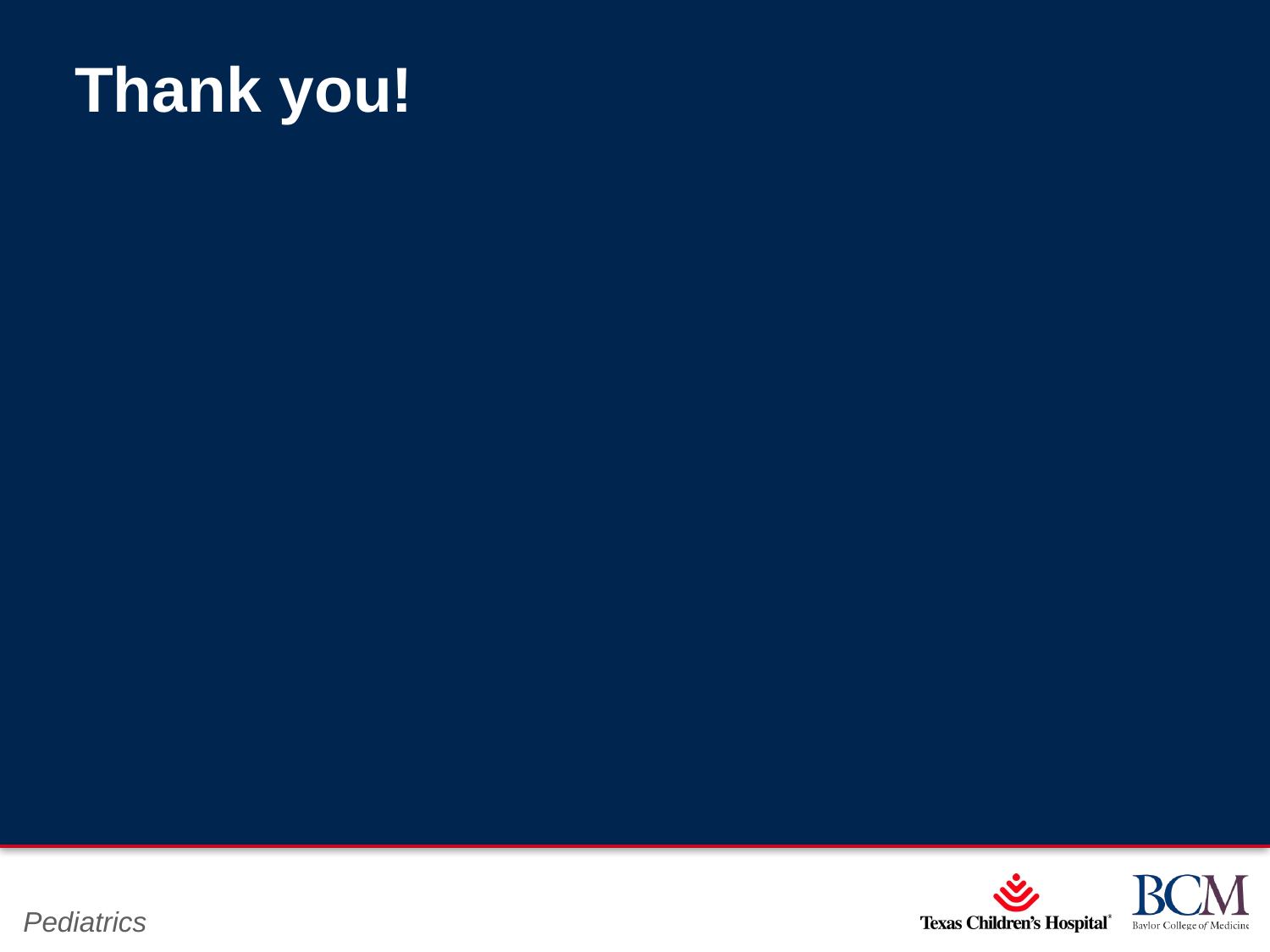

# Thank you!
