# Supplementary material for: Engaging Learners Through Modules in Quality Improvement and Patient Safety
Source: MedEdPORTAL. 2016 Oct 13;12:10482. doi: 10.15766/mep_2374-8265.10482 (PMC6440404; doi:10.15766/mep_2374-8265.10482)
Supplement: Supplementary file 1 — A. Instructor's Guide.docx B. PowerPoint Talking Points.docx C. Knowledge Survey.docx D. Attitude Survey Questions.docx E. Fundamentals of QI.pptx F. Fundamentals of Patient Safety.ppt G. Evidence-Based Practice and QI Improvement Research.pptx H. QI and PS Potpourri.pptx [file mep-12-10482-s001.zip › G. Evidence-Based Practice and QI Improvement Research.pptx]

## Slide 1
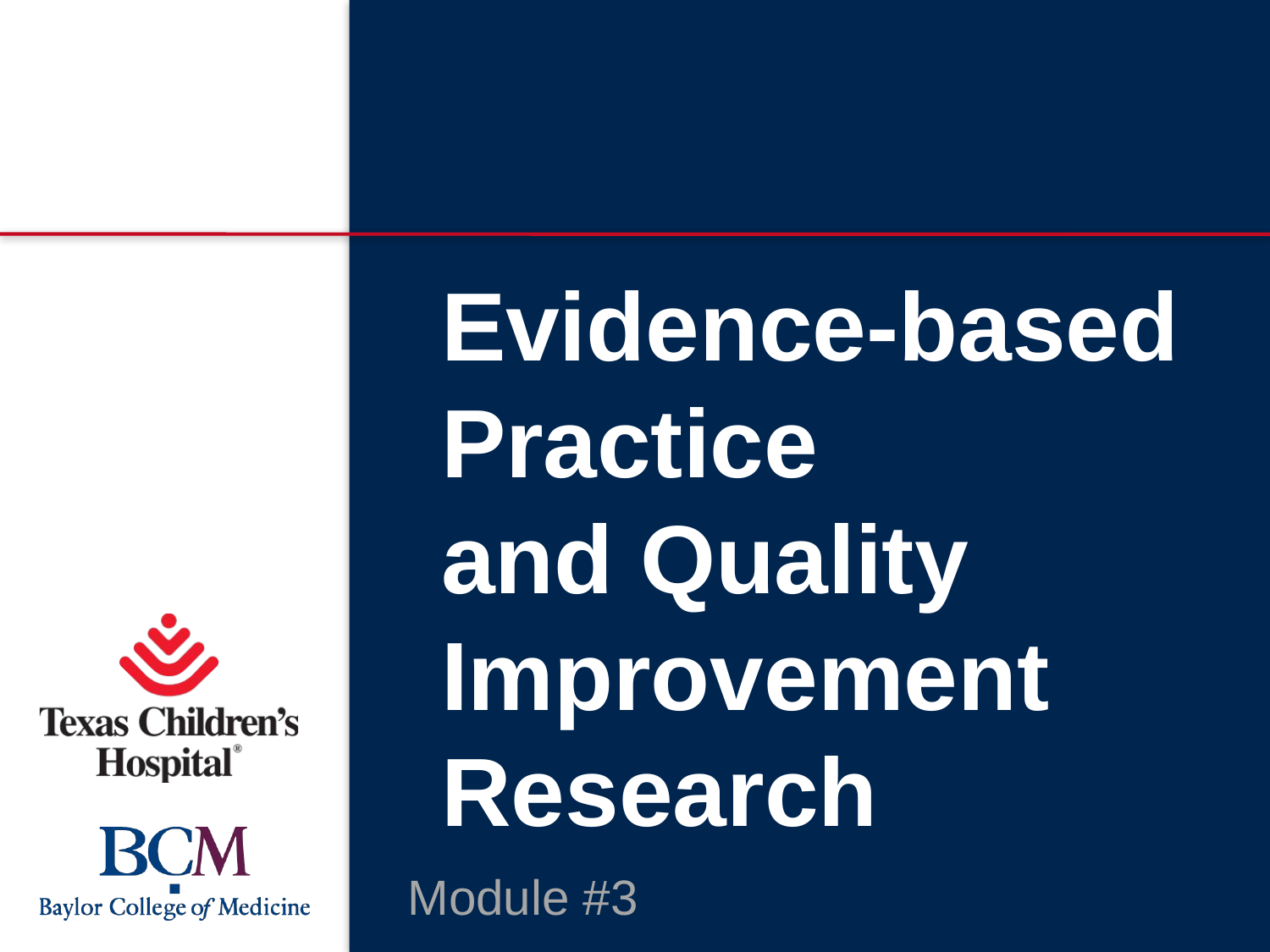

# Evidence-based Practice and Quality Improvement Research
Module #3

## Slide 2
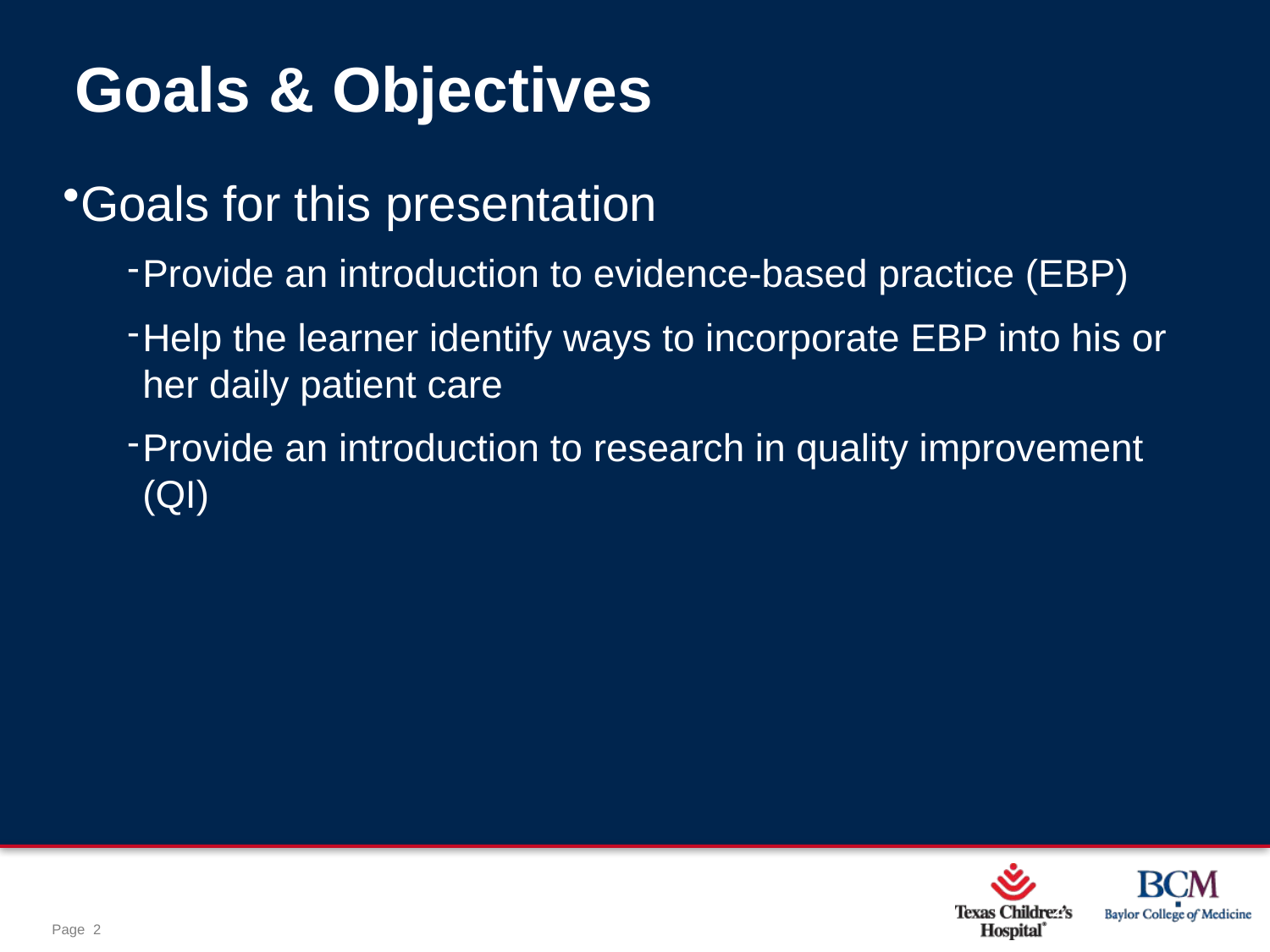

# Goals & Objectives
Goals for this presentation
Provide an introduction to evidence-based practice (EBP)
Help the learner identify ways to incorporate EBP into his or her daily patient care
Provide an introduction to research in quality improvement (QI)
2

## Slide 3
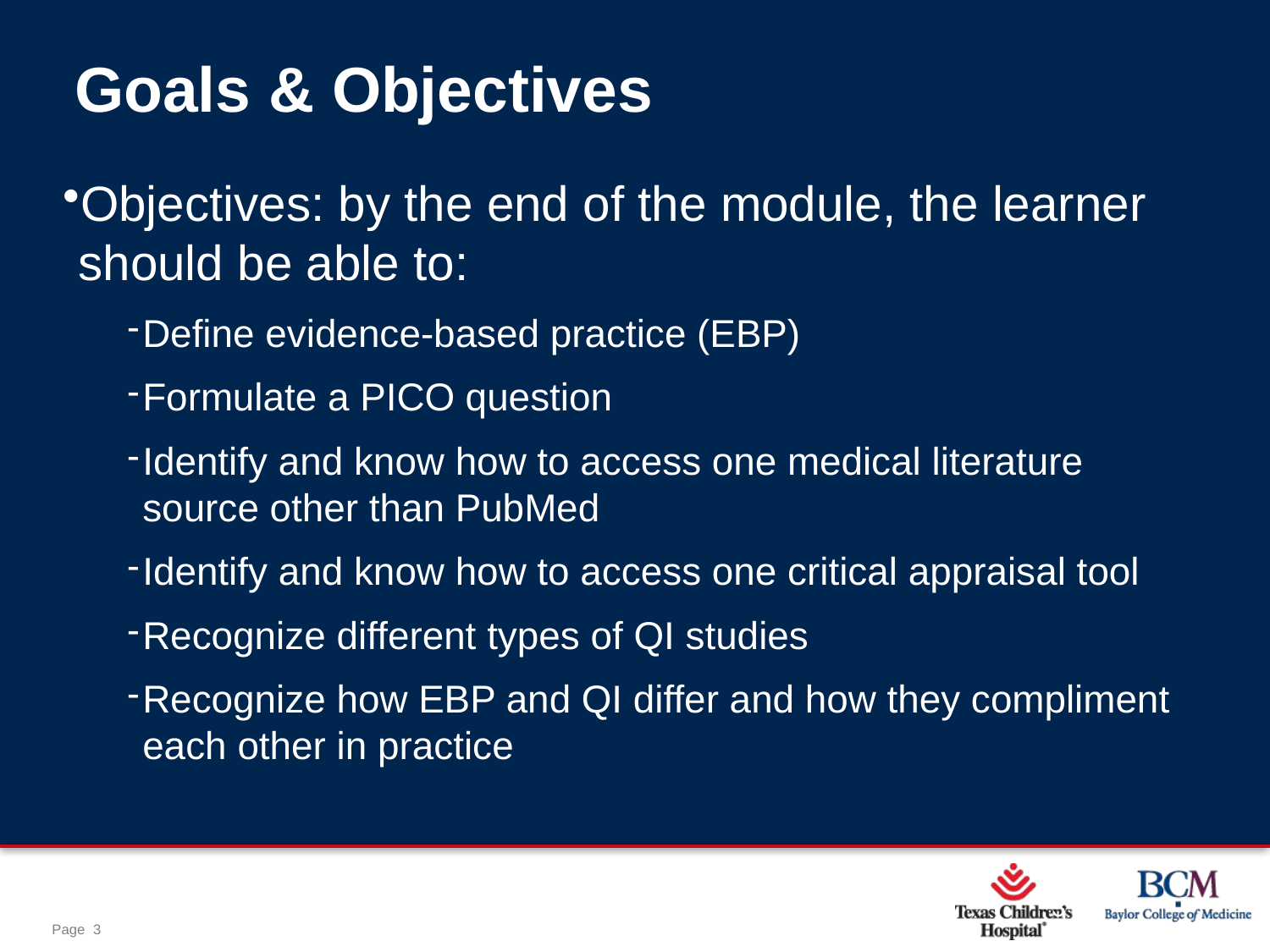

# Goals & Objectives
Objectives: by the end of the module, the learner should be able to:
Define evidence-based practice (EBP)
Formulate a PICO question
Identify and know how to access one medical literature source other than PubMed
Identify and know how to access one critical appraisal tool
Recognize different types of QI studies
Recognize how EBP and QI differ and how they compliment each other in practice
3

## Slide 4
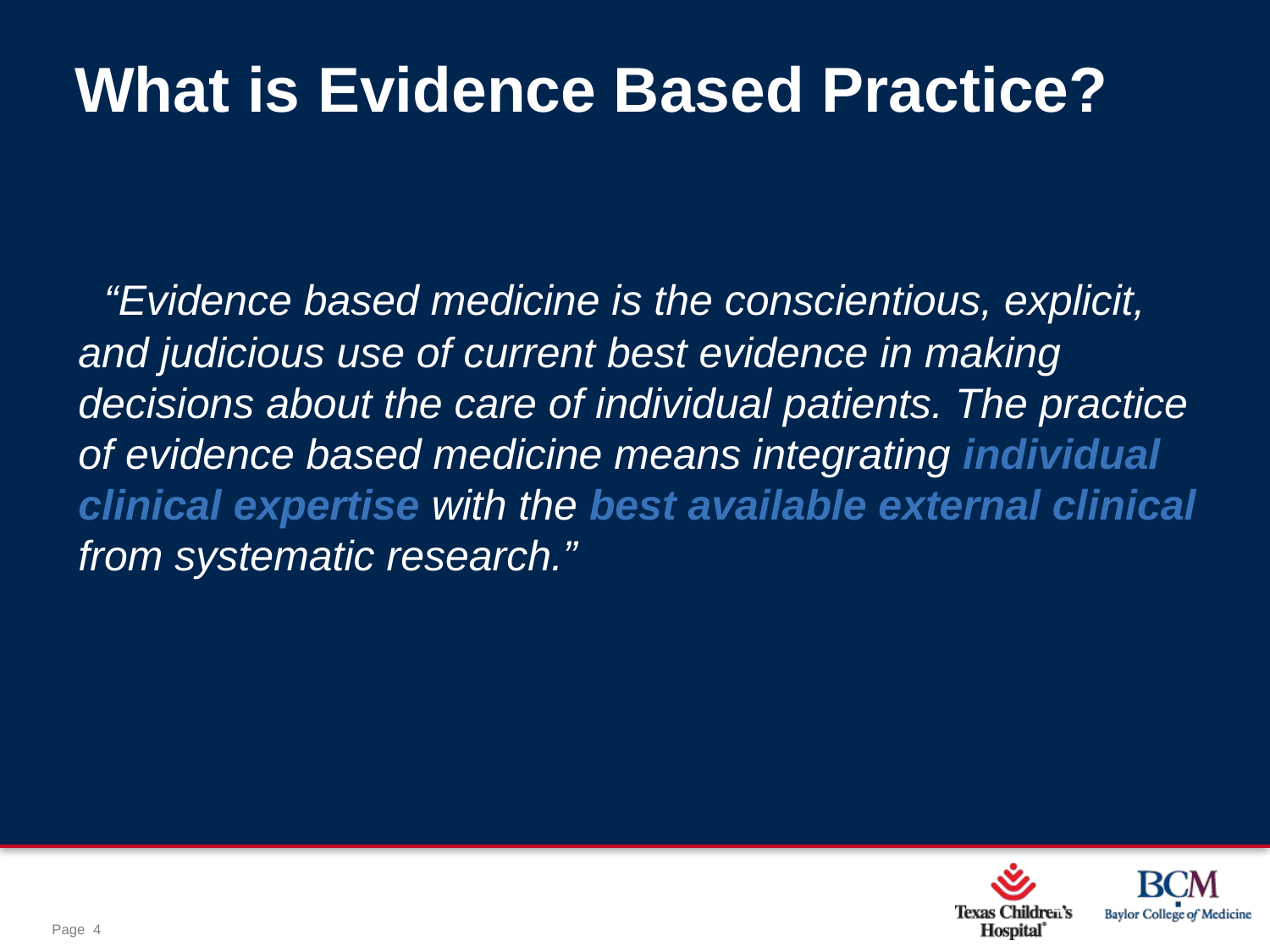

# What is Evidence Based Practice?
 “Evidence based medicine is the conscientious, explicit, and judicious use of current best evidence in making decisions about the care of individual patients. The practice of evidence based medicine means integrating individual clinical expertise with the best available external clinical from systematic research.”
Sackett et al, Evidence-based Medicine, What It Is and What It Isn’t, BMJ, 1996
4

## Slide 5
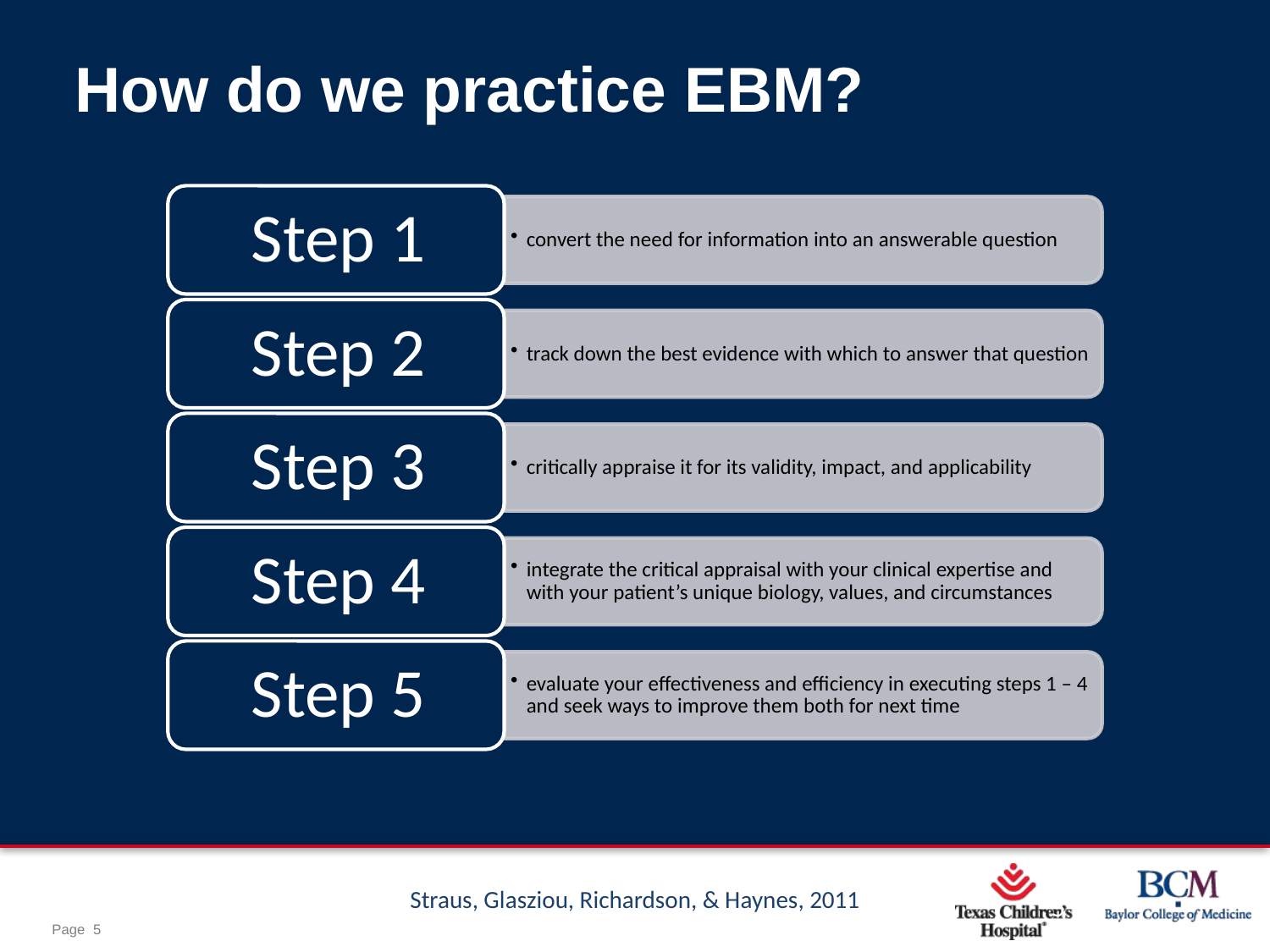

# How do we practice EBM?
Straus, Glasziou, Richardson, & Haynes, 2011
5

## Slide 6
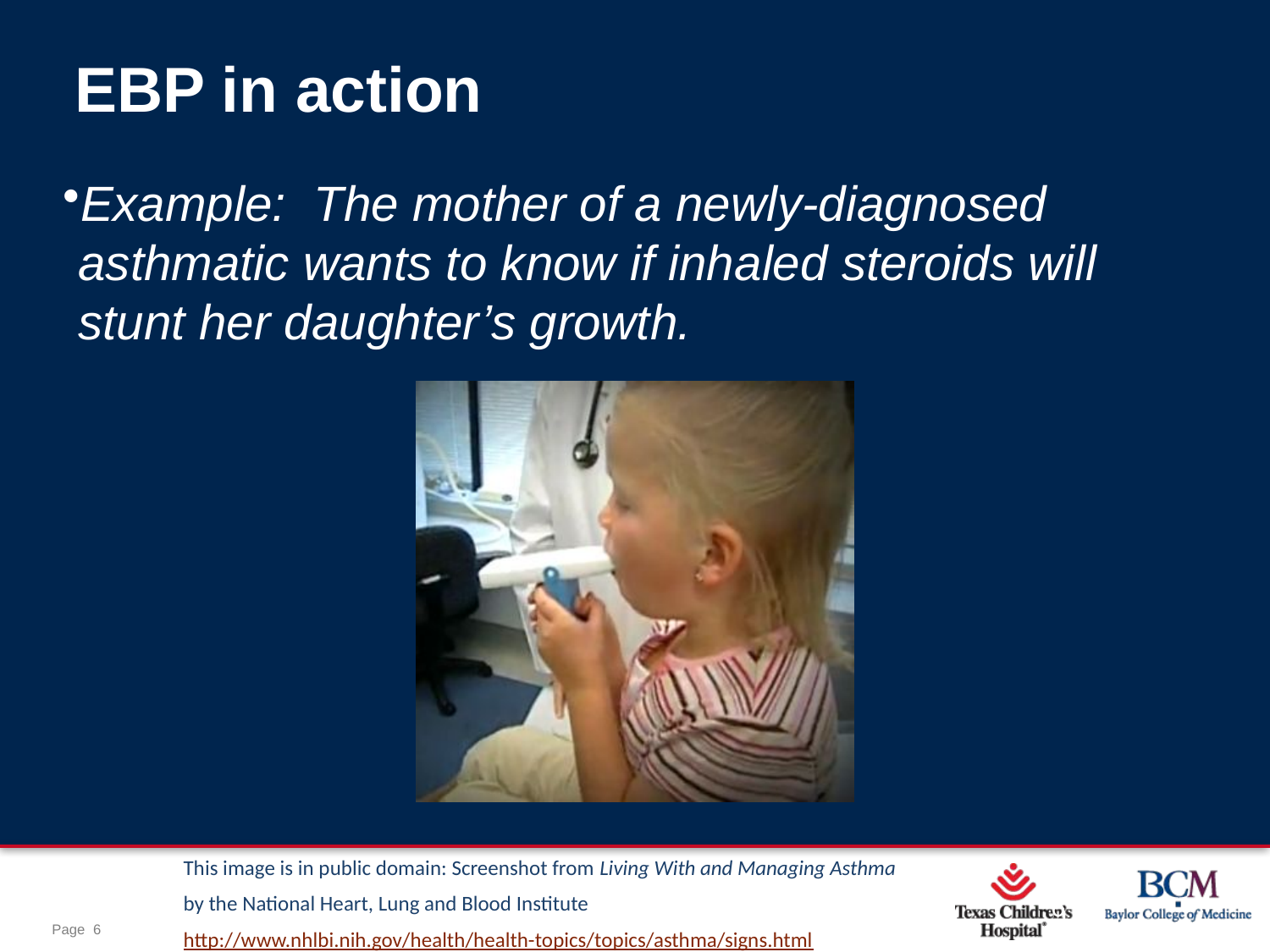

# EBP in action
Example: The mother of a newly-diagnosed asthmatic wants to know if inhaled steroids will stunt her daughter’s growth.
This image is in public domain: Screenshot from Living With and Managing Asthma
by the National Heart, Lung and Blood Institute
http://www.nhlbi.nih.gov/health/health-topics/topics/asthma/signs.html
6

## Slide 7
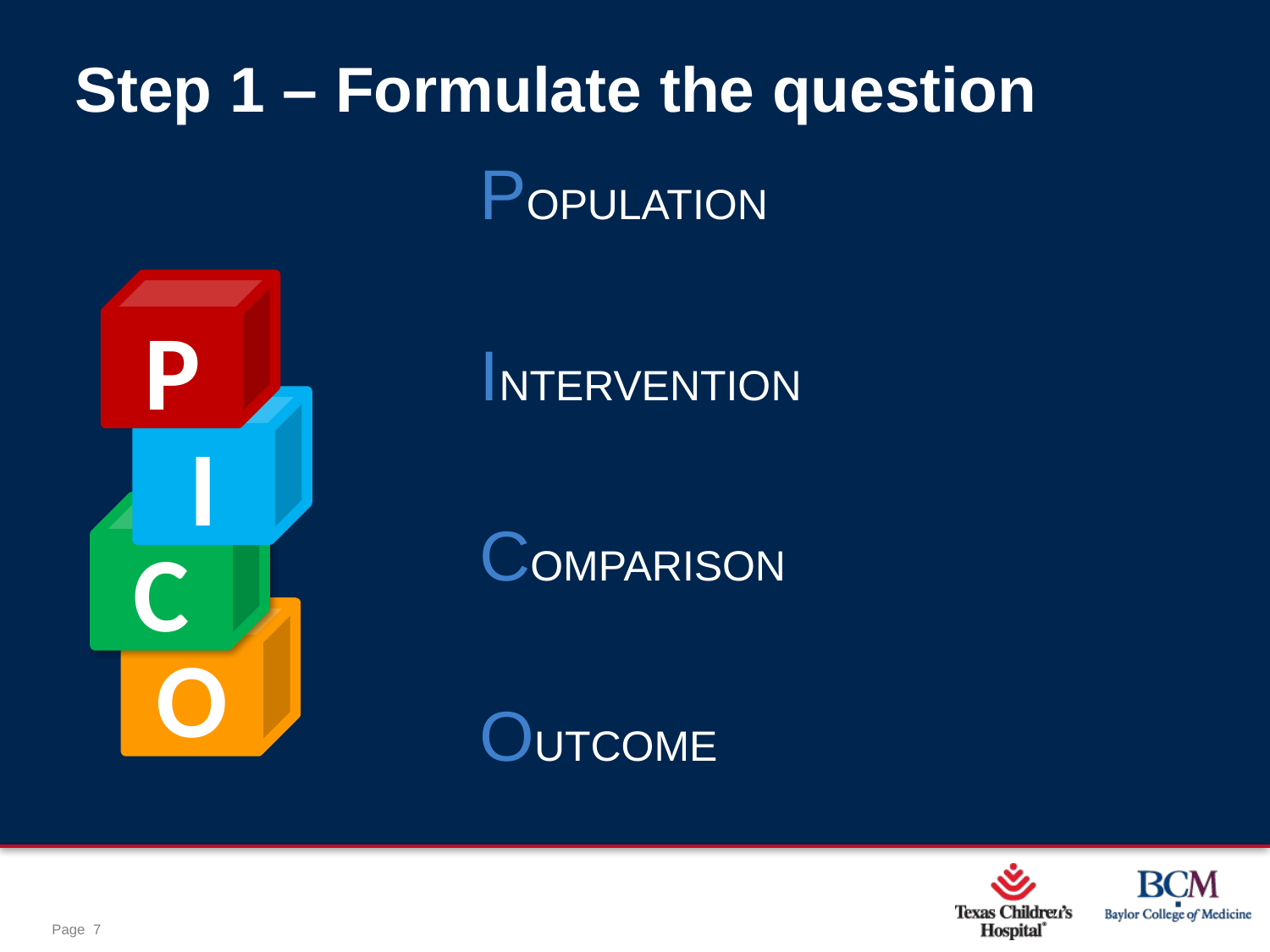

# Step 1 – Formulate the question
POPULATION
INTERVENTION
COMPARISON
OUTCOME
P
I
C
O
7

## Slide 8
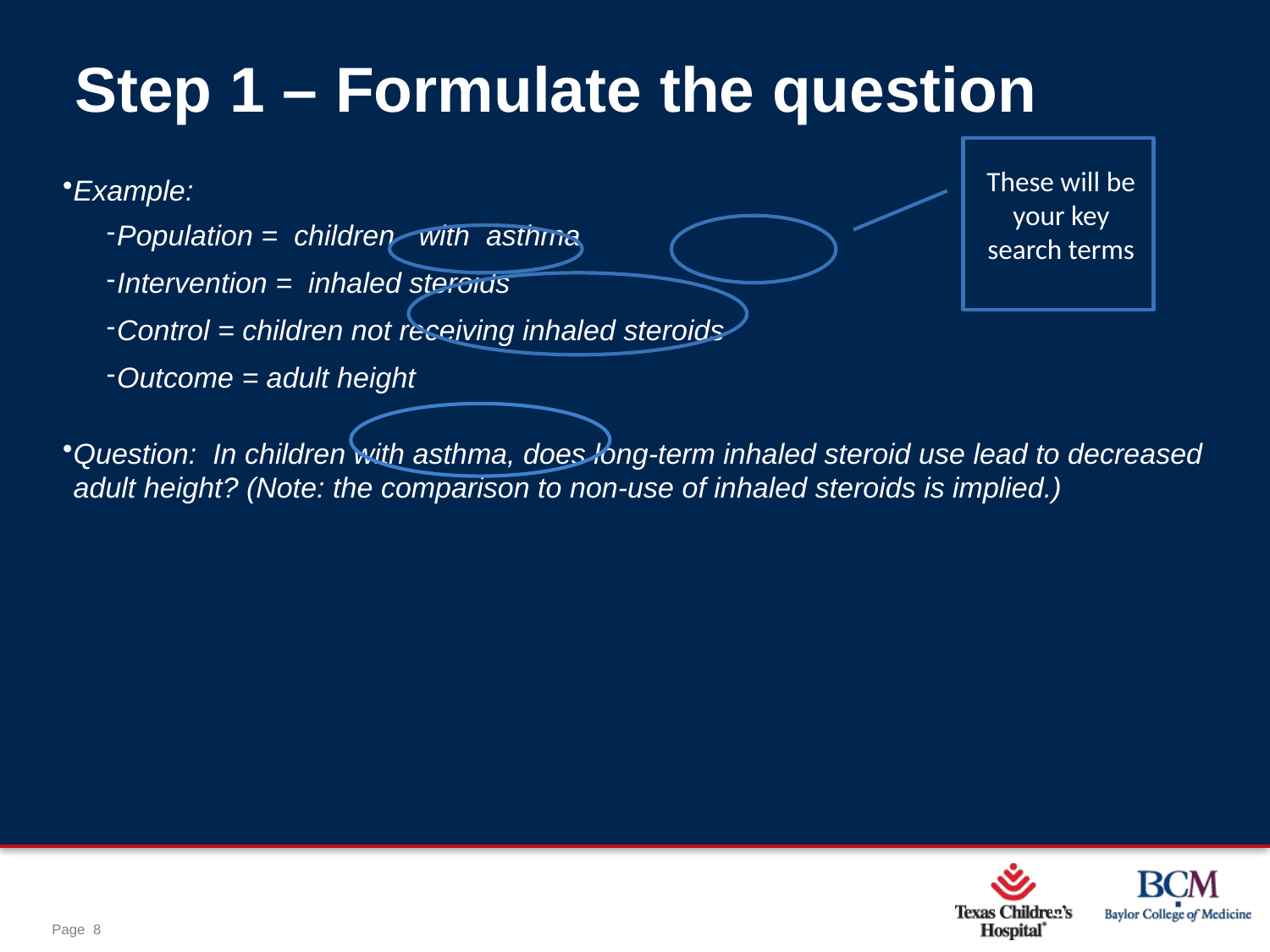

# Step 1 – Formulate the question
These will be your key search terms
Example:
Population = children with asthma
Intervention = inhaled steroids
Control = children not receiving inhaled steroids
Outcome = adult height
Question: In children with asthma, does long-term inhaled steroid use lead to decreased adult height? (Note: the comparison to non-use of inhaled steroids is implied.)
8

## Slide 9
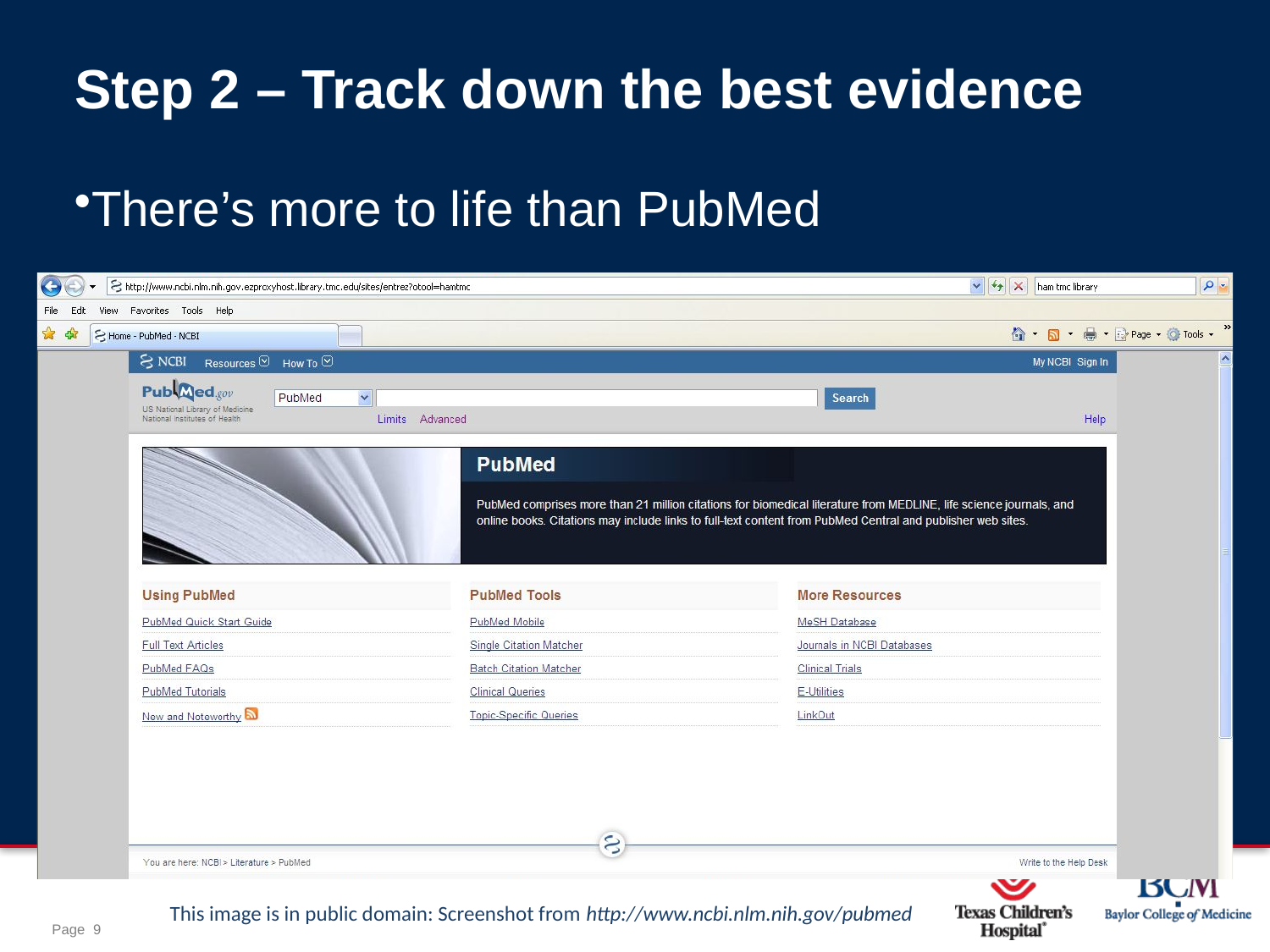

# Step 2 – Track down the best evidence
There’s more to life than PubMed
This image is in public domain: Screenshot from http://www.ncbi.nlm.nih.gov/pubmed

## Slide 10
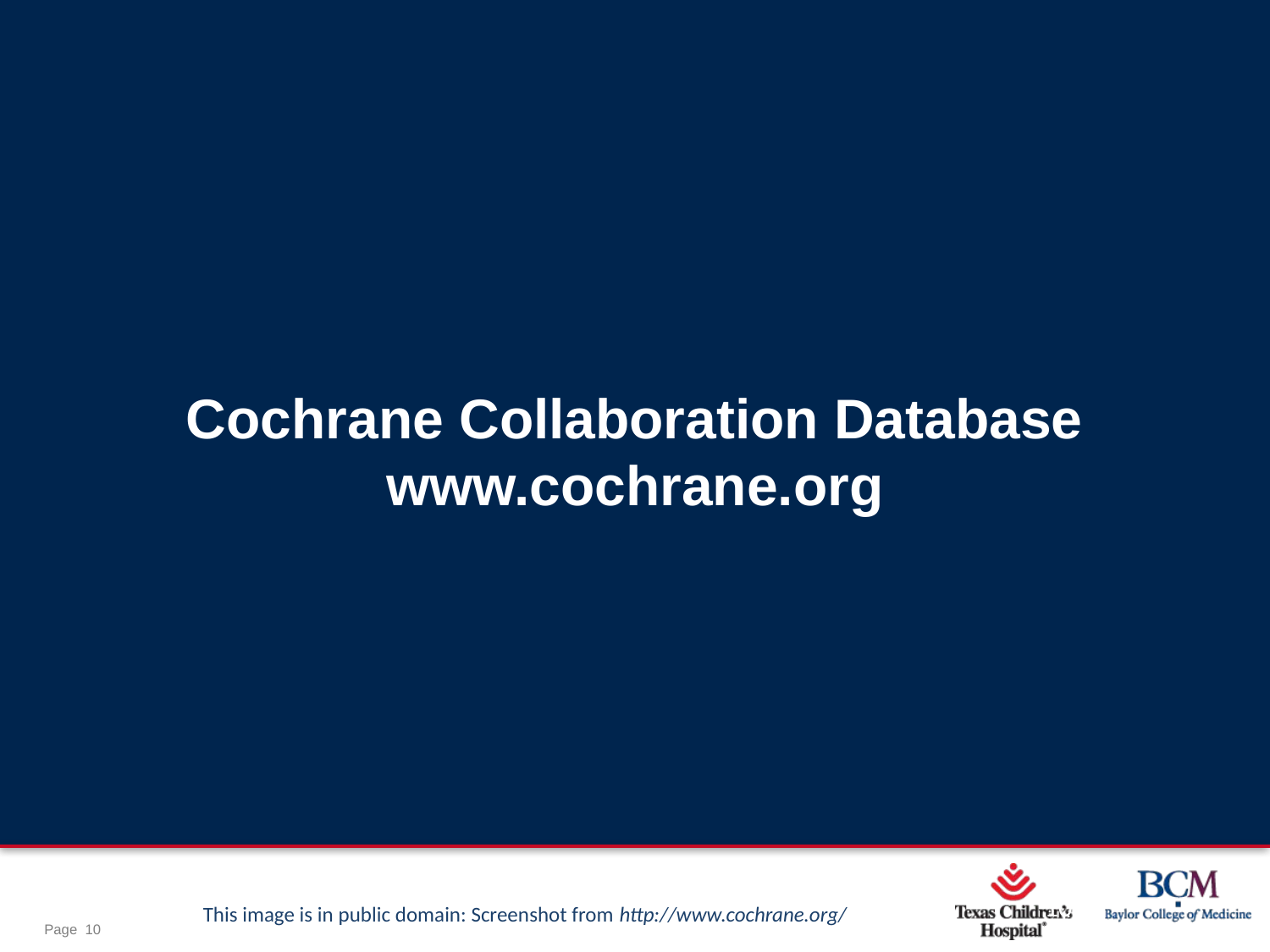

# Cochrane Collaboration Database www.cochrane.org
10
This image is in public domain: Screenshot from http://www.cochrane.org/

## Slide 11
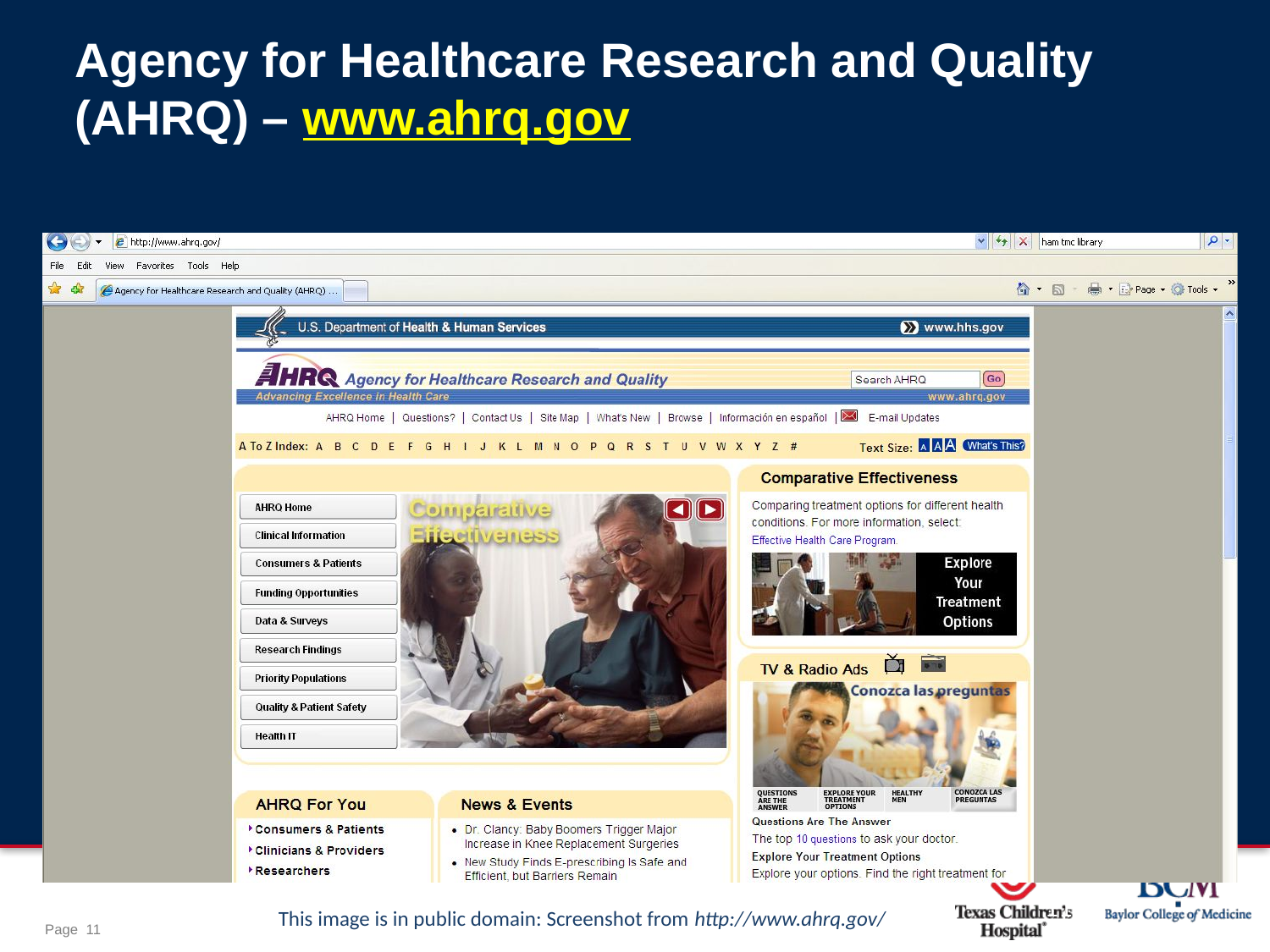

# Agency for Healthcare Research and Quality (AHRQ) – www.ahrq.gov
11
This image is in public domain: Screenshot from http://www.ahrq.gov/

## Slide 12
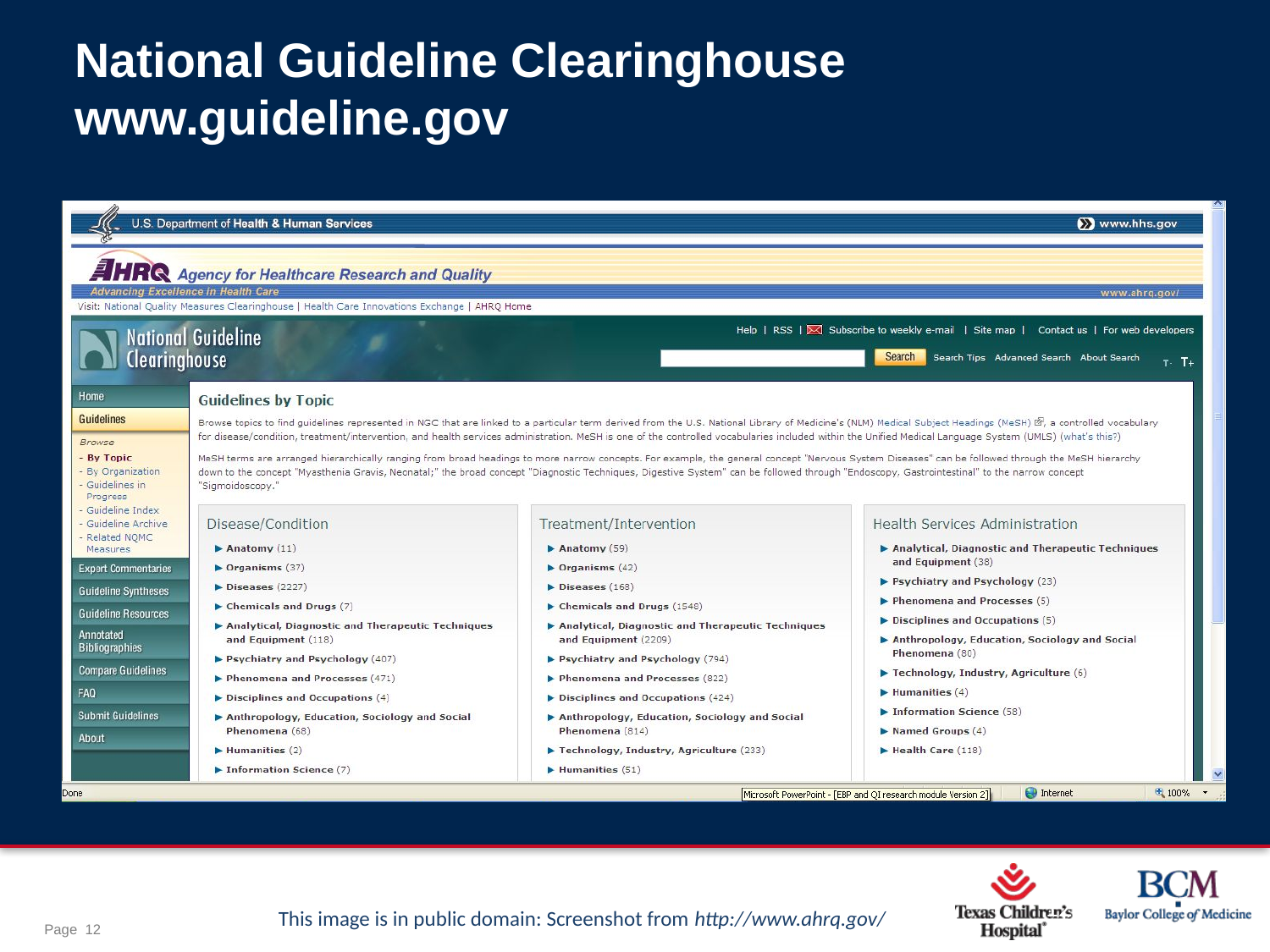

# National Guideline Clearinghouse www.guideline.gov
12
This image is in public domain: Screenshot from http://www.ahrq.gov/

## Slide 13
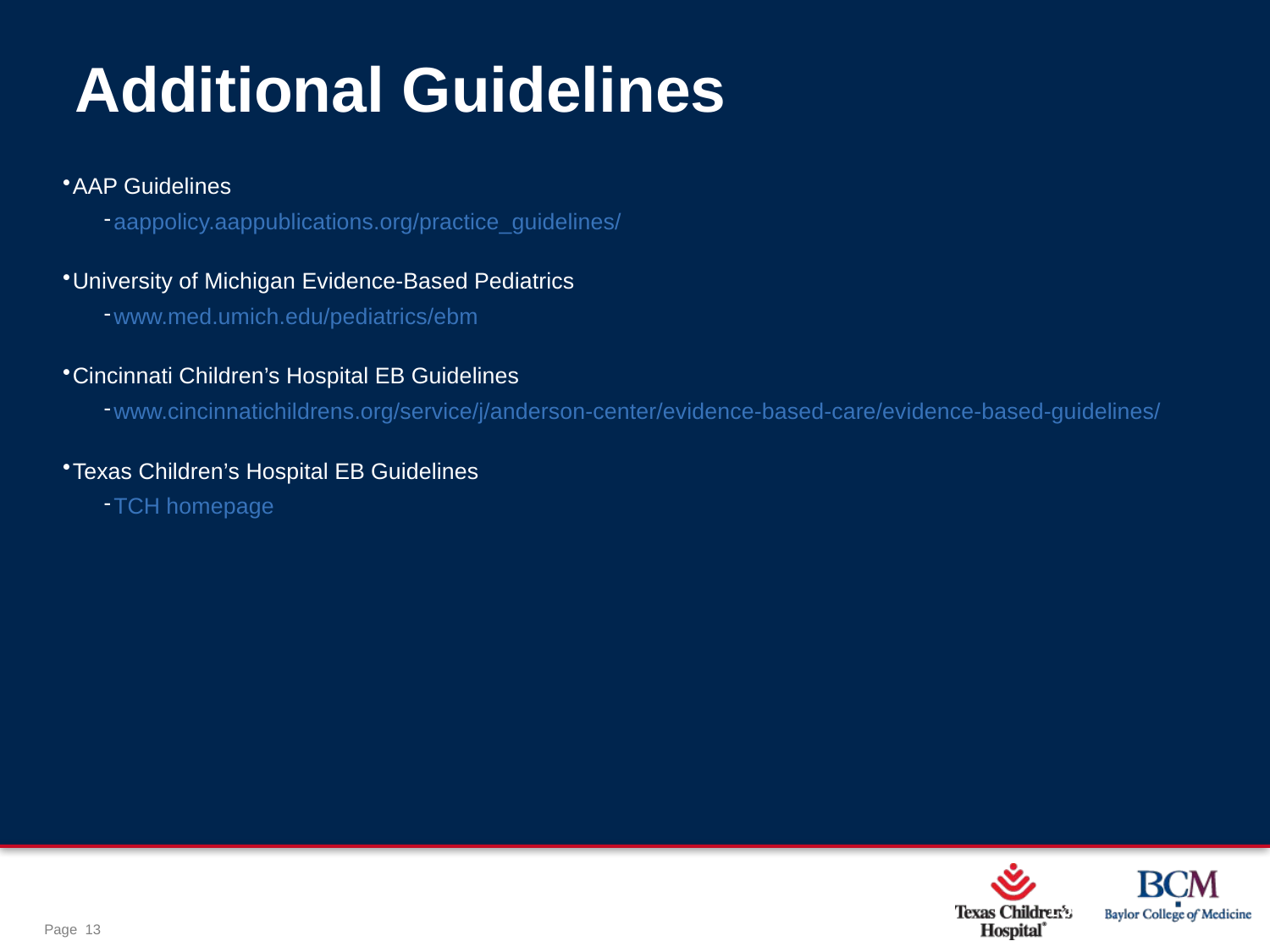

# Additional Guidelines
AAP Guidelines
aappolicy.aappublications.org/practice_guidelines/
University of Michigan Evidence-Based Pediatrics
www.med.umich.edu/pediatrics/ebm
Cincinnati Children’s Hospital EB Guidelines
www.cincinnatichildrens.org/service/j/anderson-center/evidence-based-care/evidence-based-guidelines/
Texas Children’s Hospital EB Guidelines
TCH homepage
13

## Slide 14
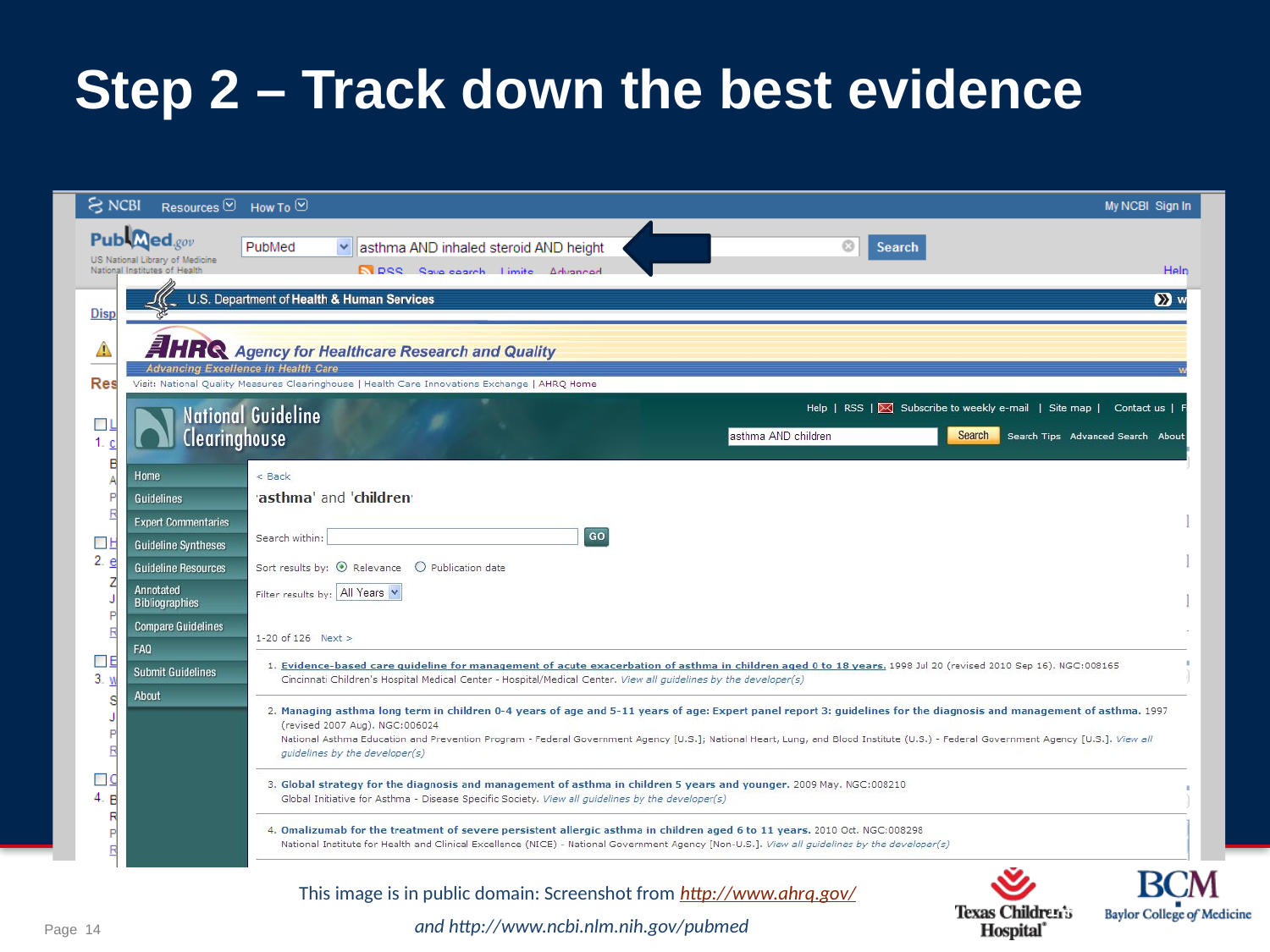

# Step 2 – Track down the best evidence
This image is in public domain: Screenshot from http://www.ahrq.gov/
and http://www.ncbi.nlm.nih.gov/pubmed
14

## Slide 15
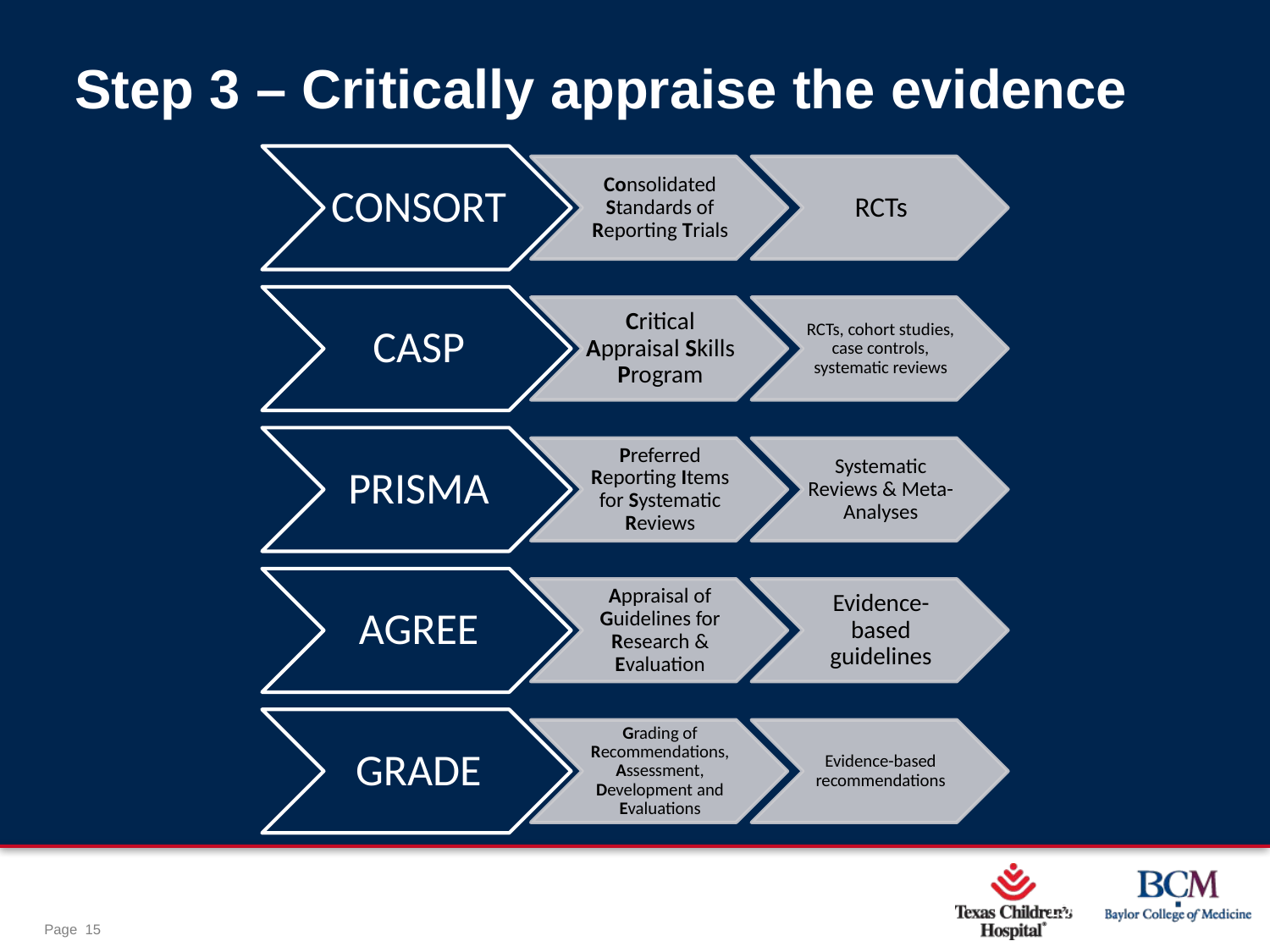

# Step 3 – Critically appraise the evidence
15

## Slide 16
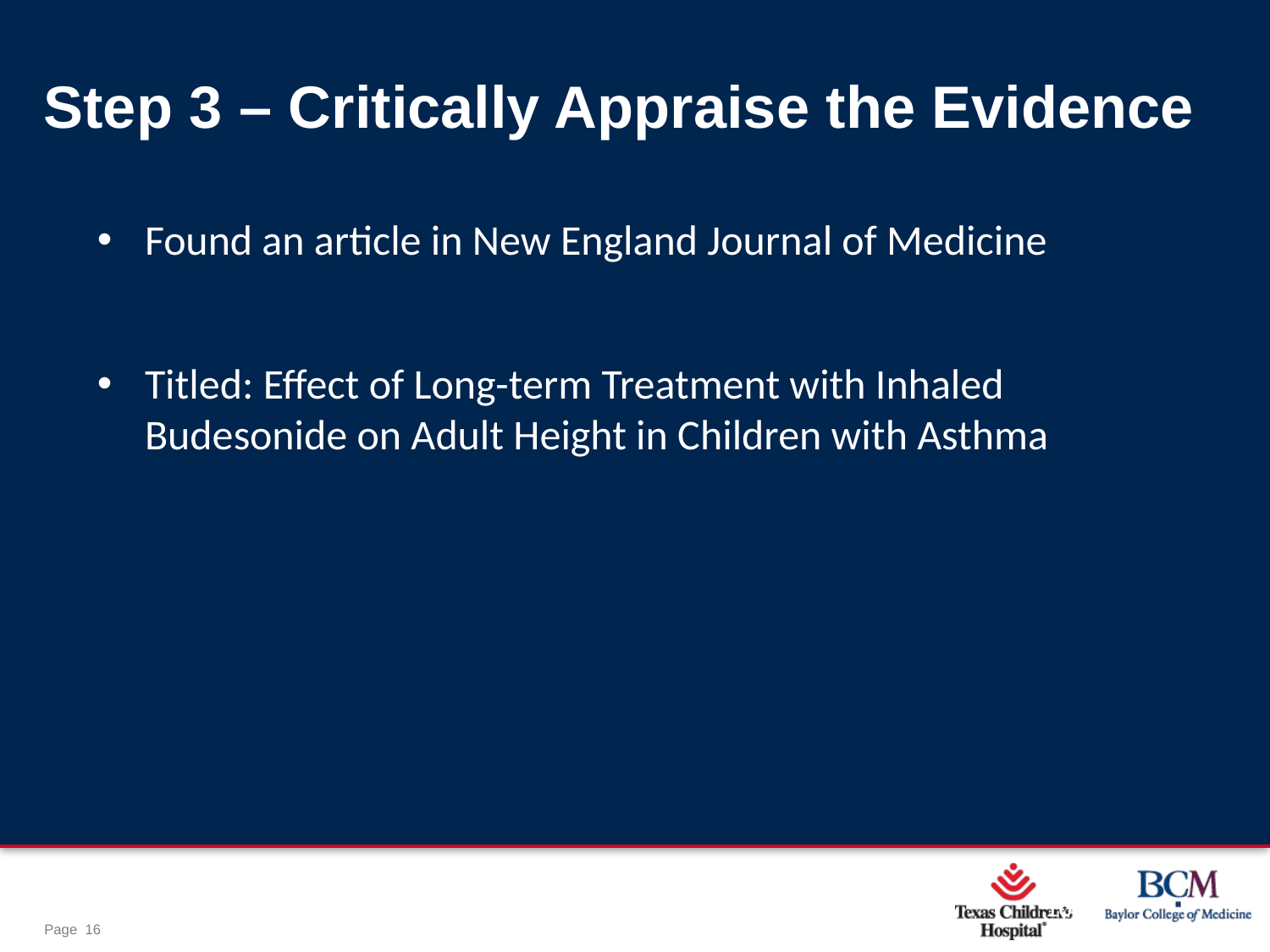

# Step 3 – Critically Appraise the Evidence
Found an article in New England Journal of Medicine
Titled: Effect of Long-term Treatment with Inhaled Budesonide on Adult Height in Children with Asthma
16

## Slide 17
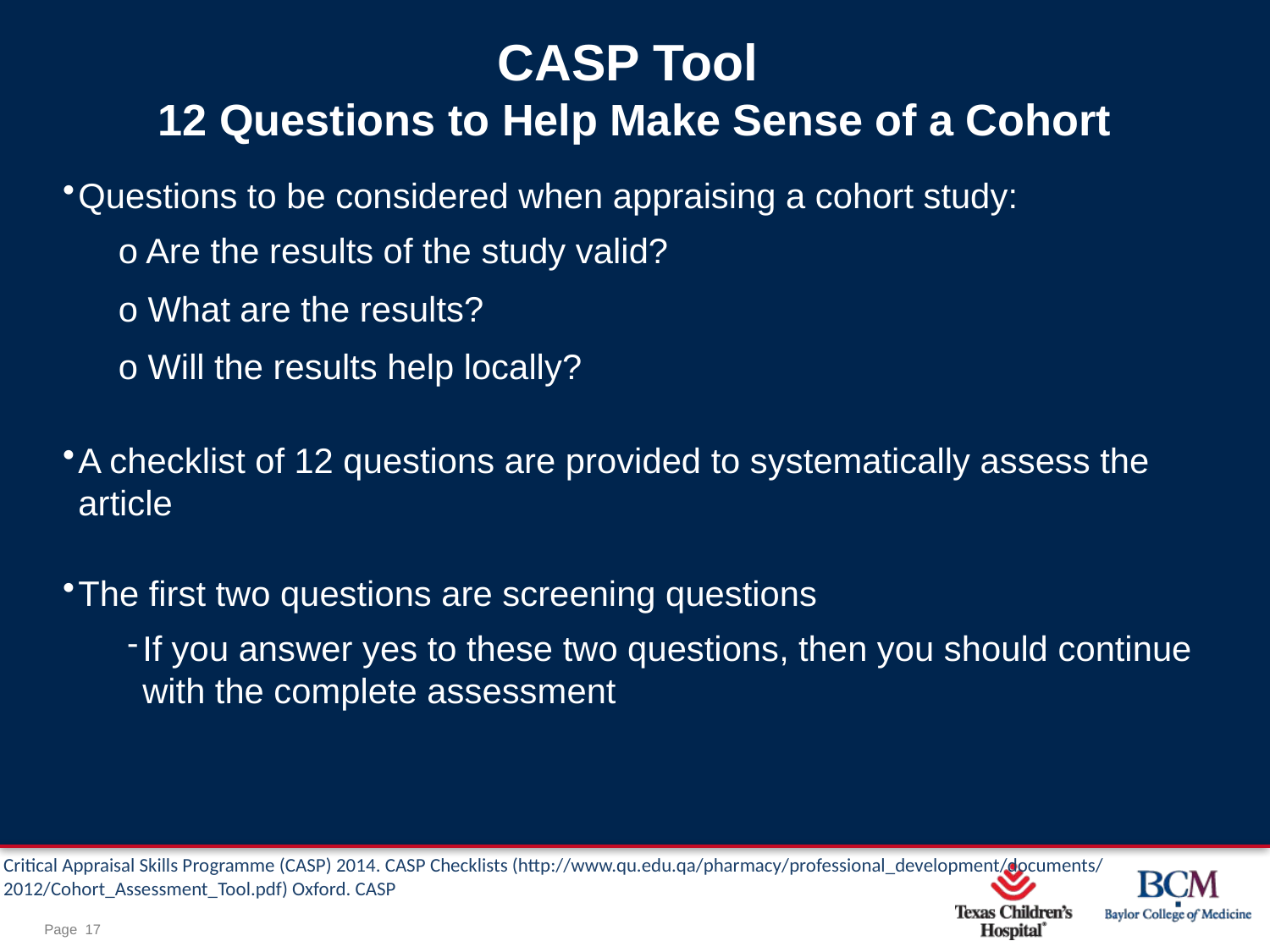

# CASP Tool 12 Questions to Help Make Sense of a Cohort
Questions to be considered when appraising a cohort study:
o Are the results of the study valid?
o What are the results?
o Will the results help locally?
A checklist of 12 questions are provided to systematically assess the article
The first two questions are screening questions
If you answer yes to these two questions, then you should continue with the complete assessment
Critical Appraisal Skills Programme (CASP) 2014. CASP Checklists (http://www.qu.edu.qa/pharmacy/professional_development/documents/2012/Cohort_Assessment_Tool.pdf) Oxford. CASP

## Slide 18
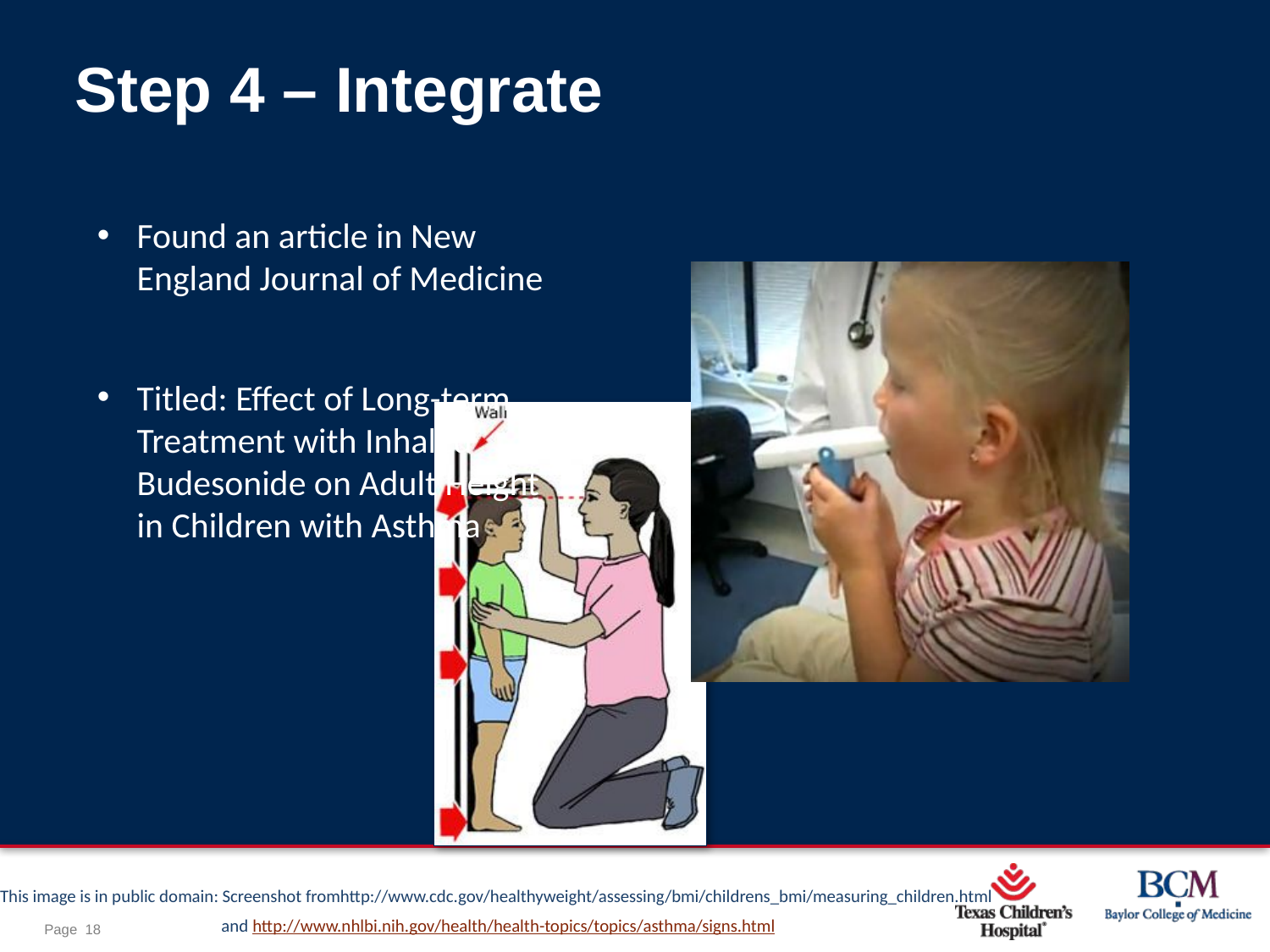

# Step 4 – Integrate
Found an article in New England Journal of Medicine
Titled: Effect of Long-term Treatment with Inhaled Budesonide on Adult Height in Children with Asthma
This image is in public domain: Screenshot fromhttp://www.cdc.gov/healthyweight/assessing/bmi/childrens_bmi/measuring_children.html
and http://www.nhlbi.nih.gov/health/health-topics/topics/asthma/signs.html

## Slide 19
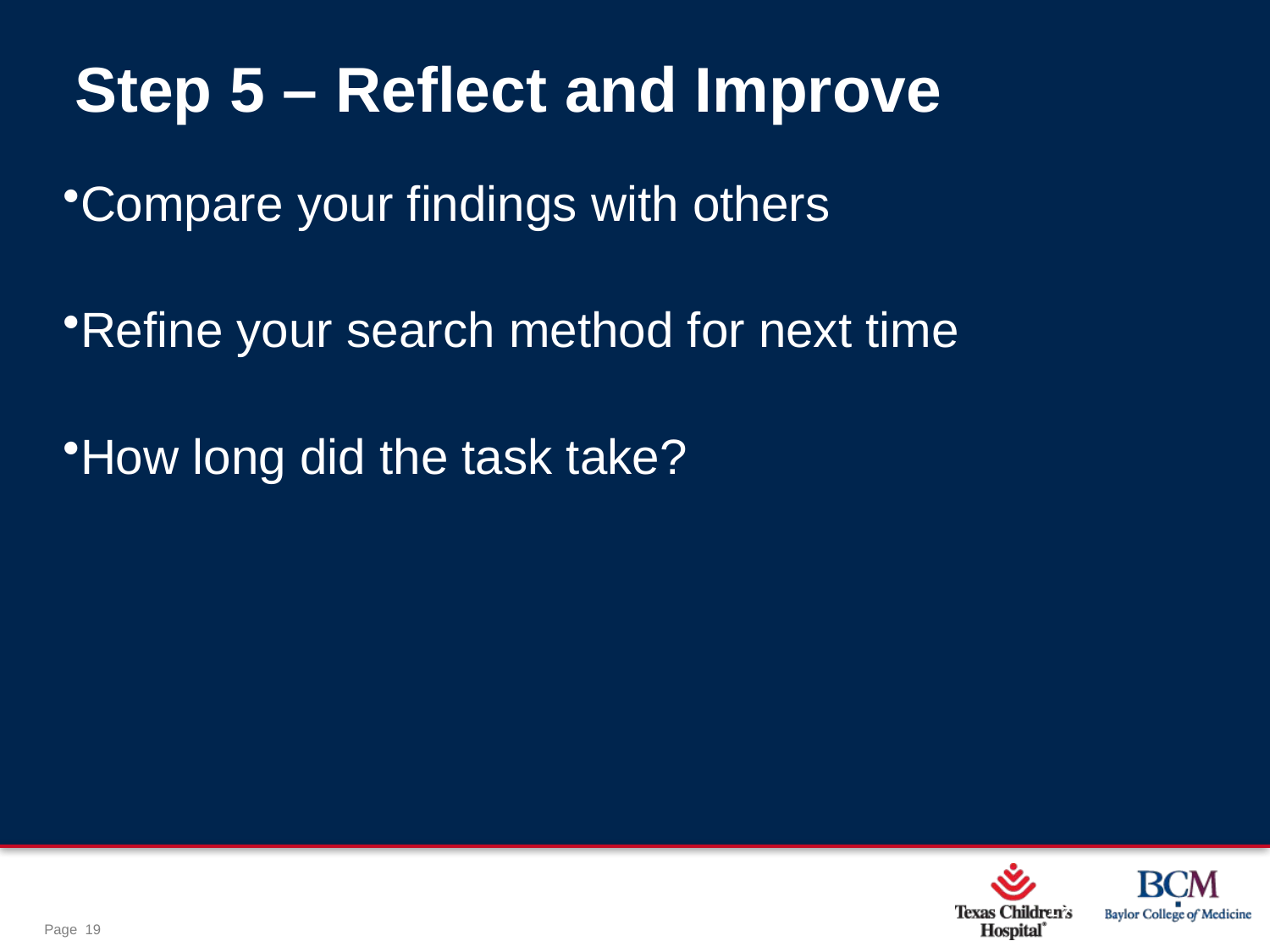

# Step 5 – Reflect and Improve
Compare your findings with others
Refine your search method for next time
How long did the task take?
19

## Slide 20
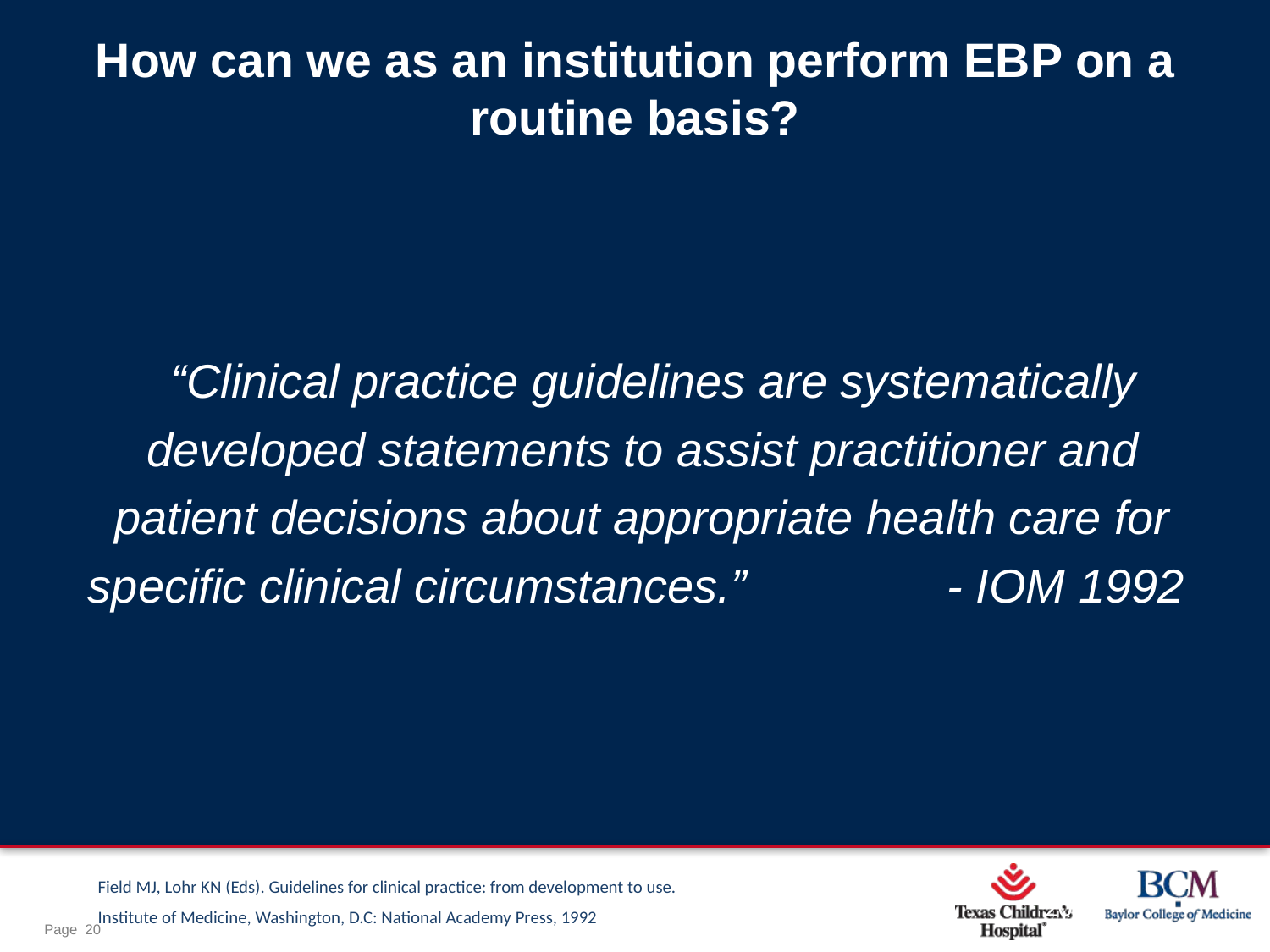

# How can we as an institution perform EBP on a routine basis?
 “Clinical practice guidelines are systematically developed statements to assist practitioner and patient decisions about appropriate health care for specific clinical circumstances.” 			 - IOM 1992
Field MJ, Lohr KN (Eds). Guidelines for clinical practice: from development to use.
Institute of Medicine, Washington, D.C: National Academy Press, 1992
20

## Slide 21
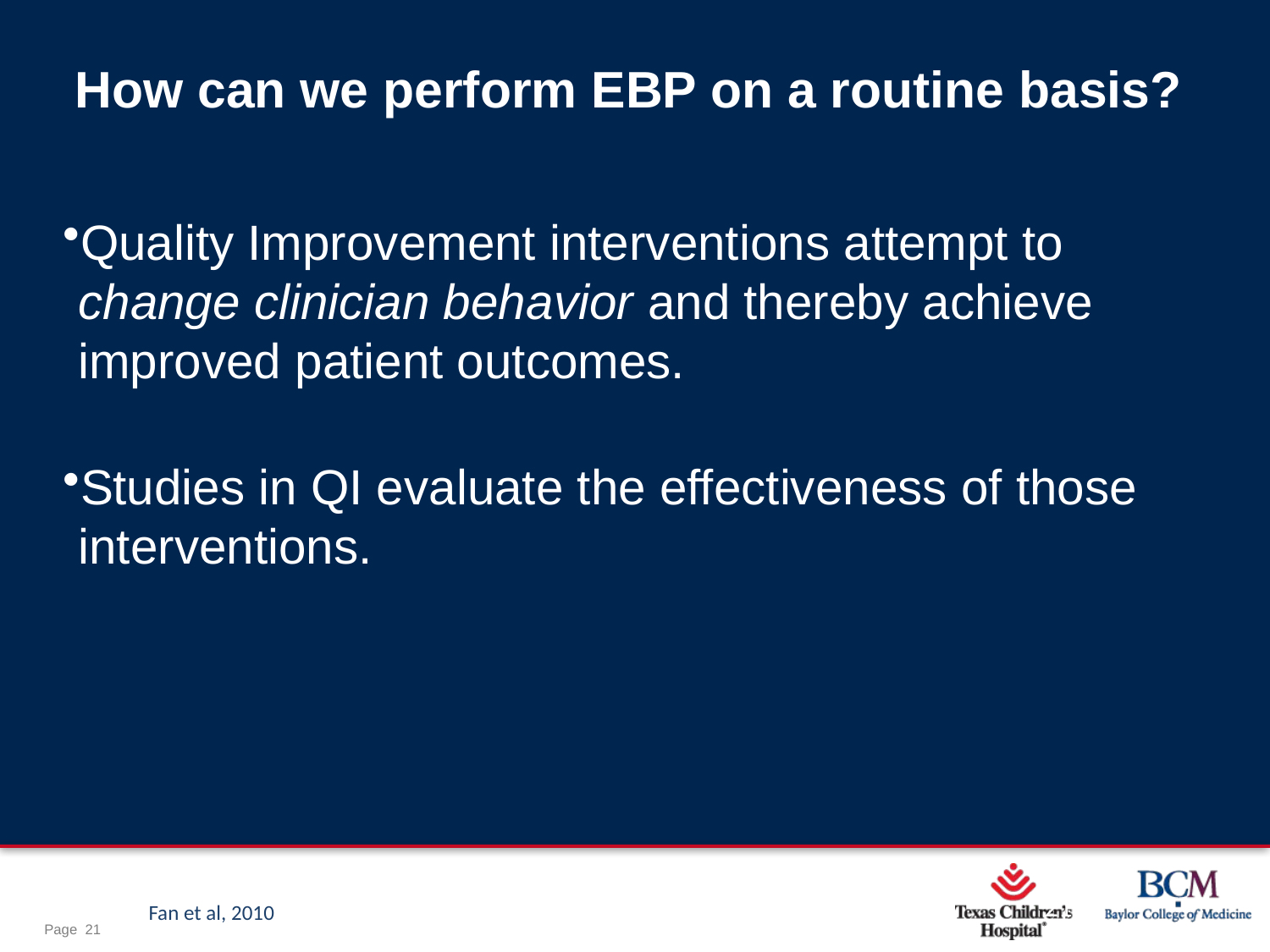

# How can we perform EBP on a routine basis?
Quality Improvement interventions attempt to change clinician behavior and thereby achieve improved patient outcomes.
Studies in QI evaluate the effectiveness of those interventions.
21
Fan et al, 2010

## Slide 22
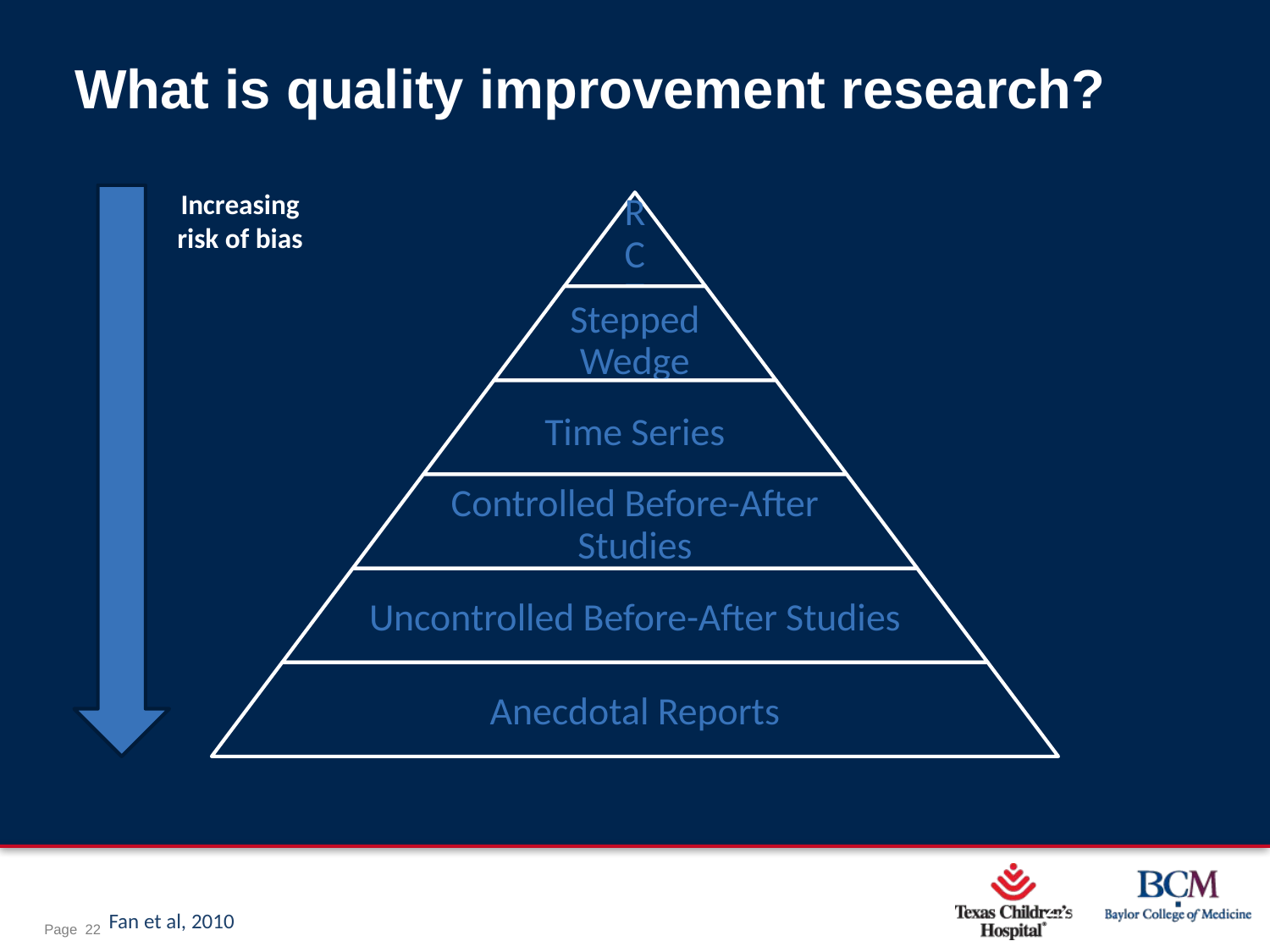

# What is quality improvement research?
Increasing risk of bias
22
Fan et al, 2010

## Slide 23
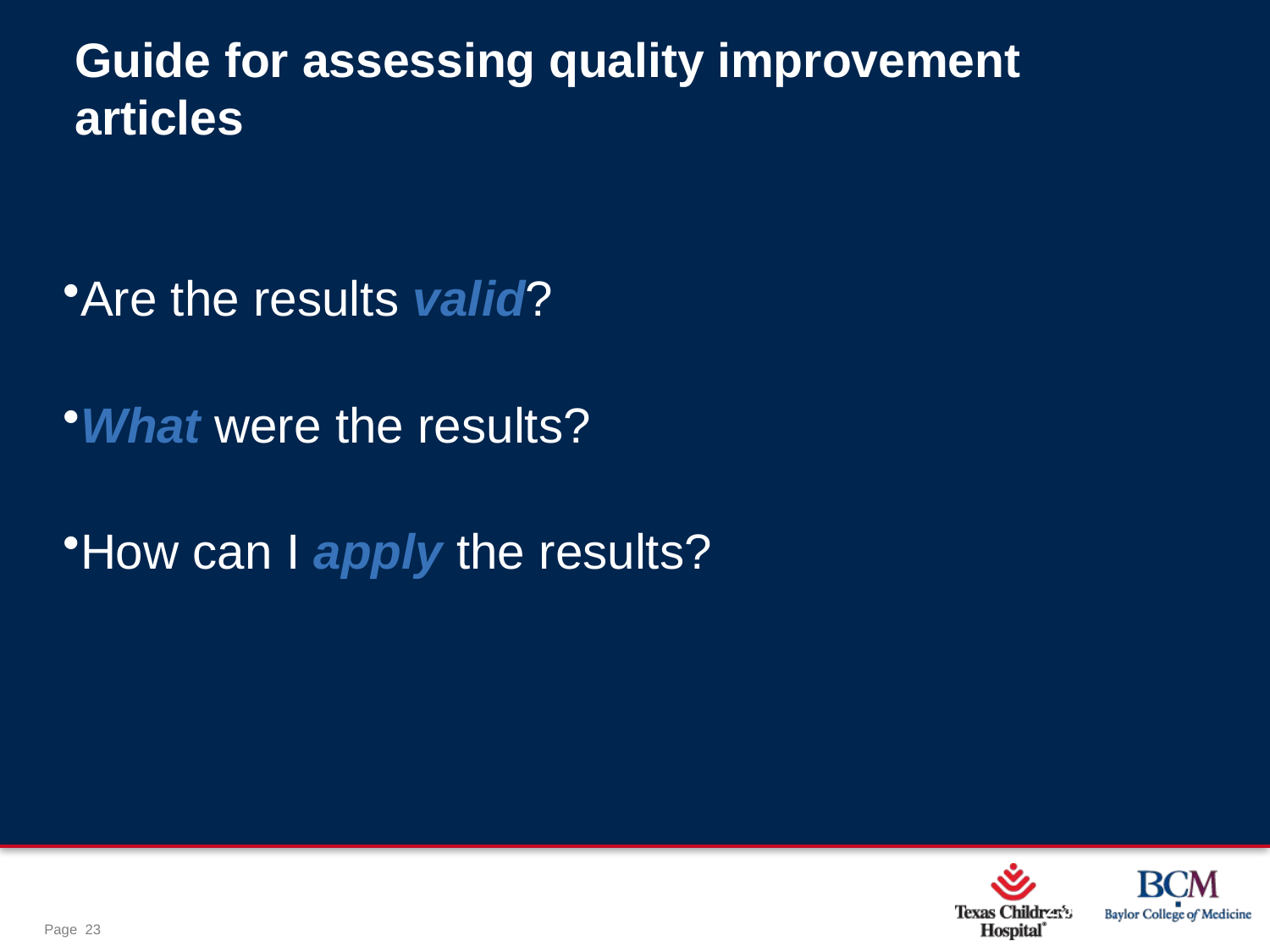

# Guide for assessing quality improvement articles
Are the results valid?
What were the results?
How can I apply the results?
Fan et al, 2010
23

## Slide 24
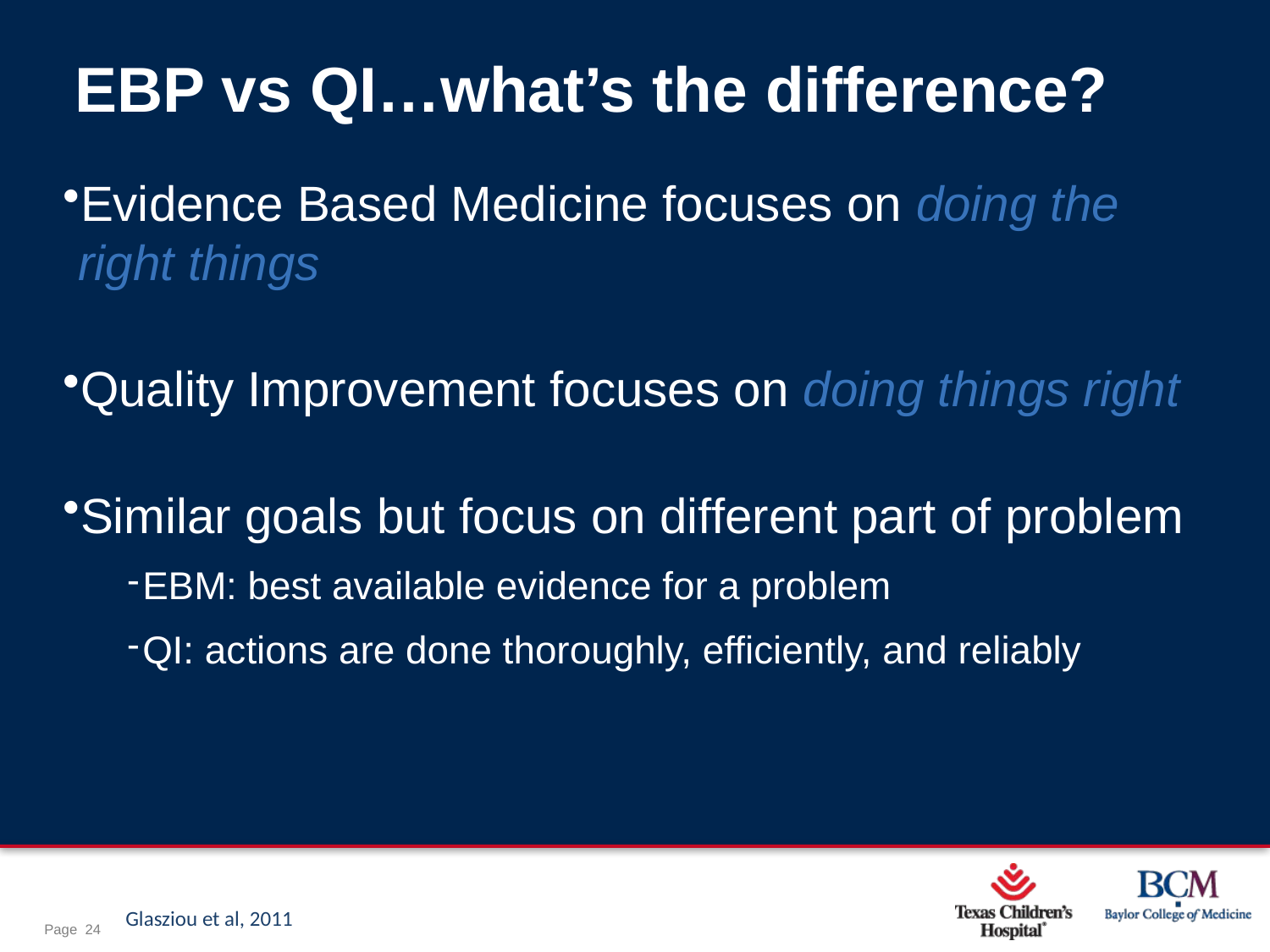

# EBP vs QI…what’s the difference?
Evidence Based Medicine focuses on doing the right things
Quality Improvement focuses on doing things right
Similar goals but focus on different part of problem
EBM: best available evidence for a problem
QI: actions are done thoroughly, efficiently, and reliably
Glasziou et al, 2011

## Slide 25
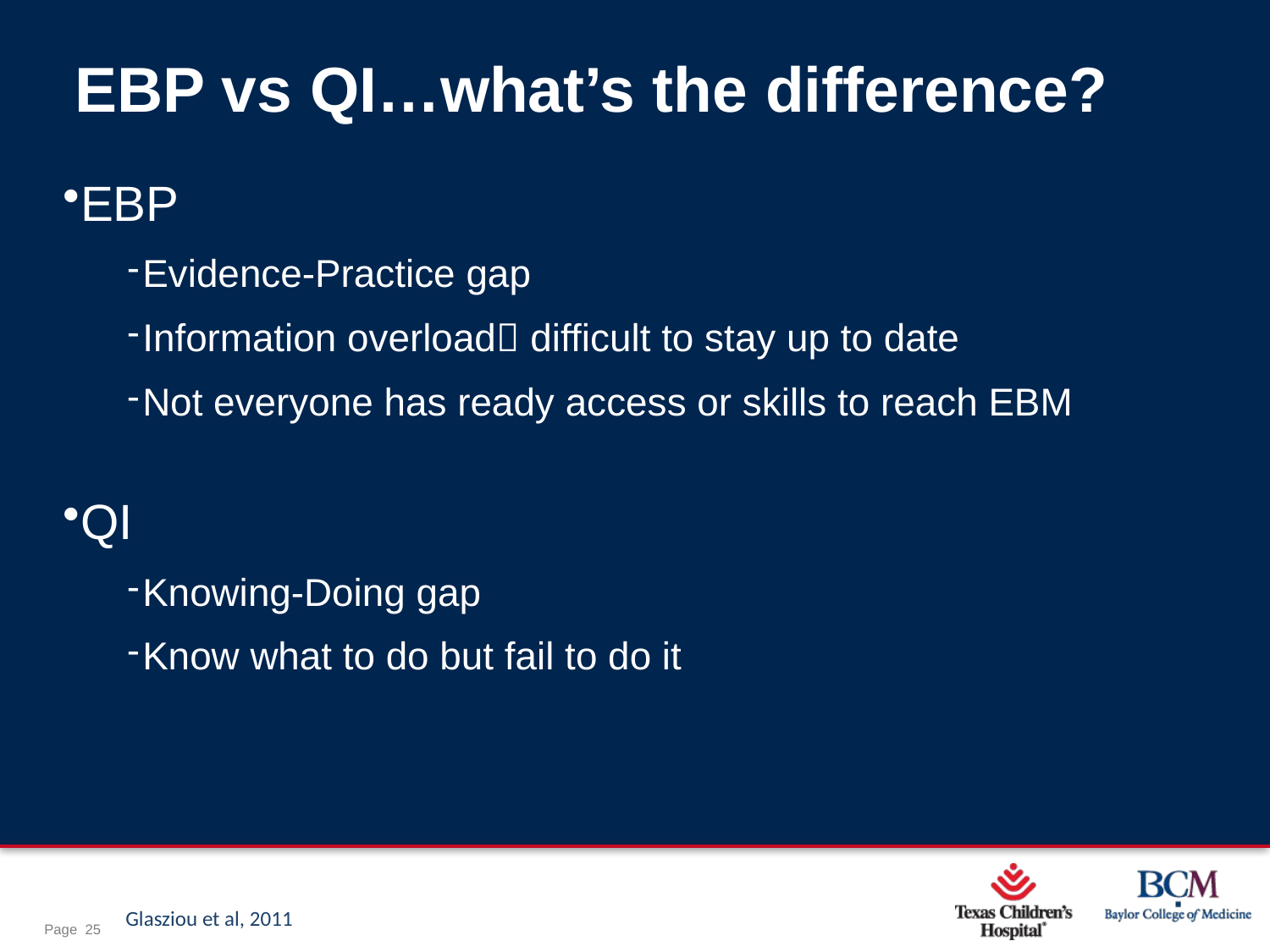

# EBP vs QI…what’s the difference?
EBP
Evidence-Practice gap
Information overload difficult to stay up to date
Not everyone has ready access or skills to reach EBM
QI
Knowing-Doing gap
Know what to do but fail to do it
Glasziou et al, 2011

## Slide 26
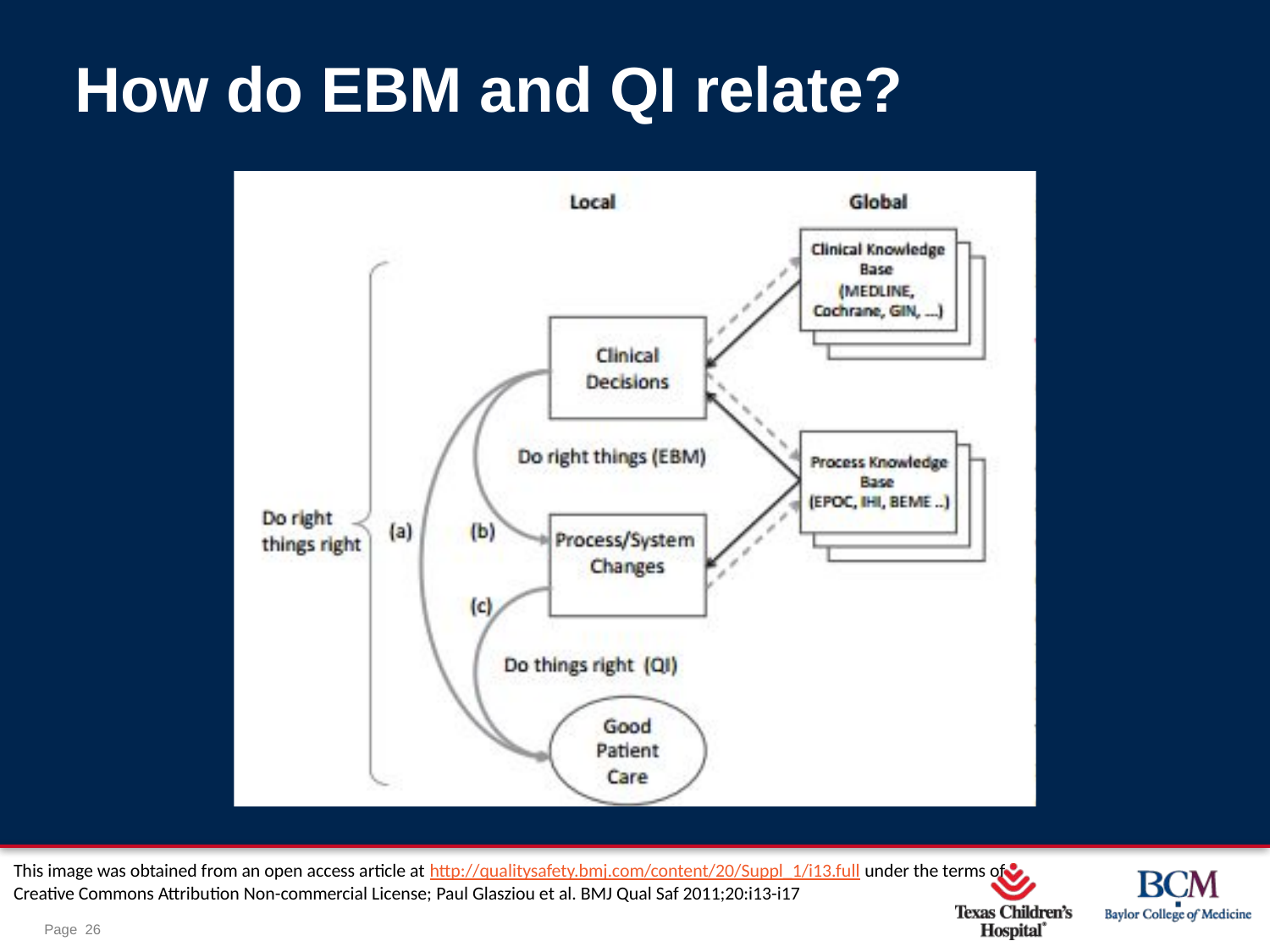

# How do EBM and QI relate?
This image was obtained from an open access article at http://qualitysafety.bmj.com/content/20/Suppl_1/i13.full under the terms of Creative Commons Attribution Non-commercial License; Paul Glasziou et al. BMJ Qual Saf 2011;20:i13-i17

## Slide 27
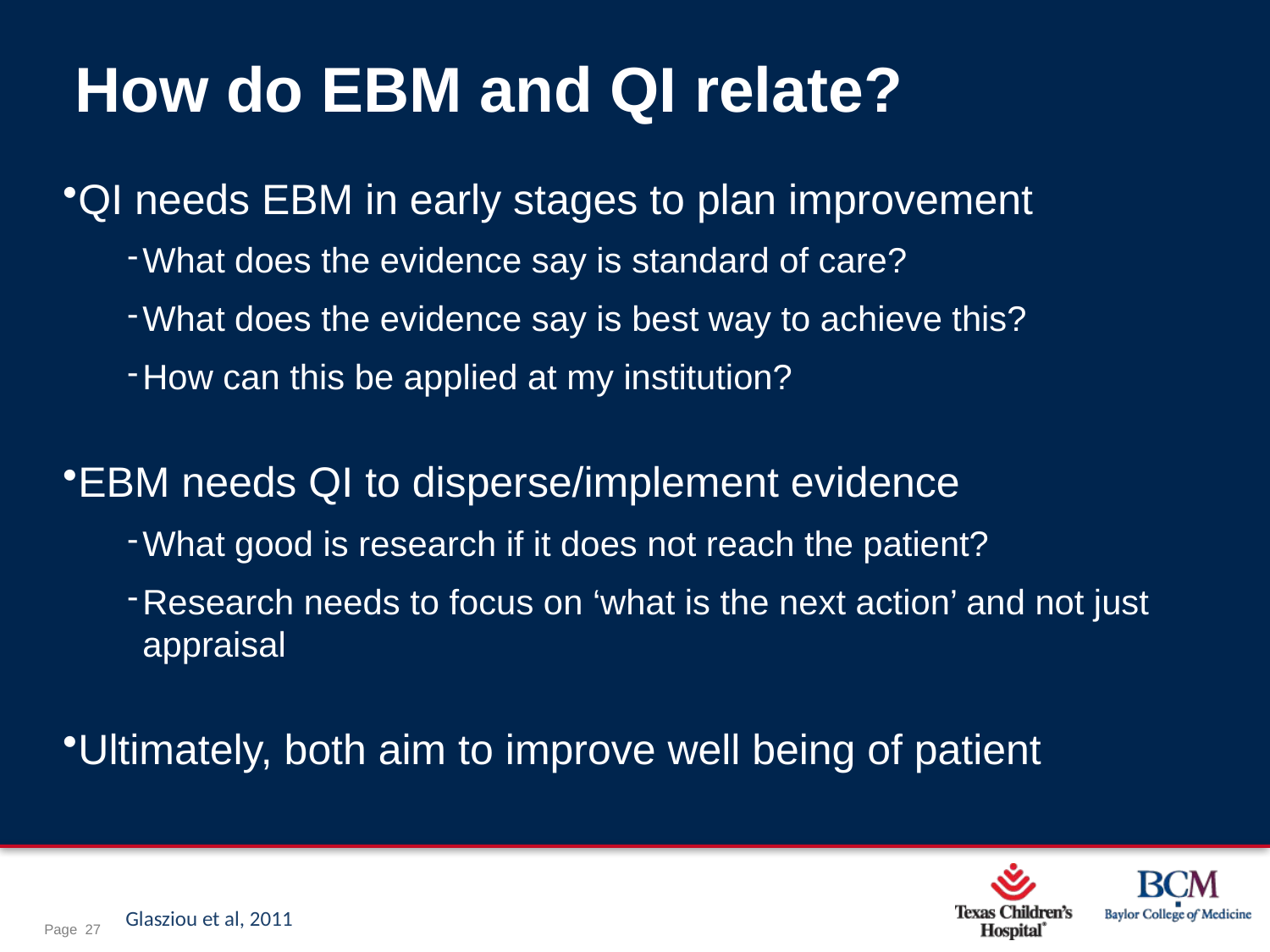

# How do EBM and QI relate?
QI needs EBM in early stages to plan improvement
What does the evidence say is standard of care?
What does the evidence say is best way to achieve this?
How can this be applied at my institution?
EBM needs QI to disperse/implement evidence
What good is research if it does not reach the patient?
Research needs to focus on ‘what is the next action’ and not just appraisal
Ultimately, both aim to improve well being of patient
Glasziou et al, 2011

## Slide 28
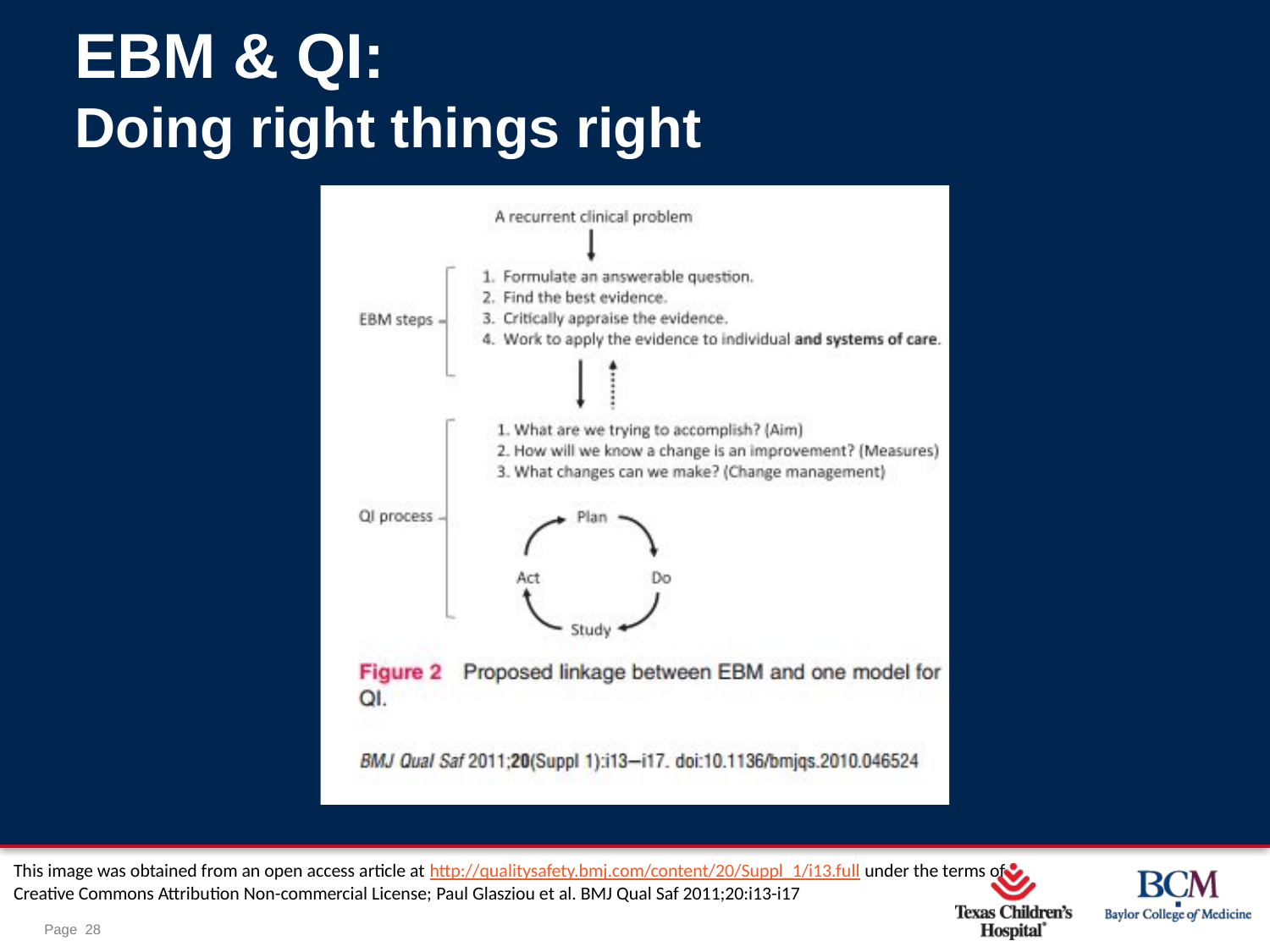

# EBM & QI: Doing right things right
This image was obtained from an open access article at http://qualitysafety.bmj.com/content/20/Suppl_1/i13.full under the terms of Creative Commons Attribution Non-commercial License; Paul Glasziou et al. BMJ Qual Saf 2011;20:i13-i17

## Slide 29
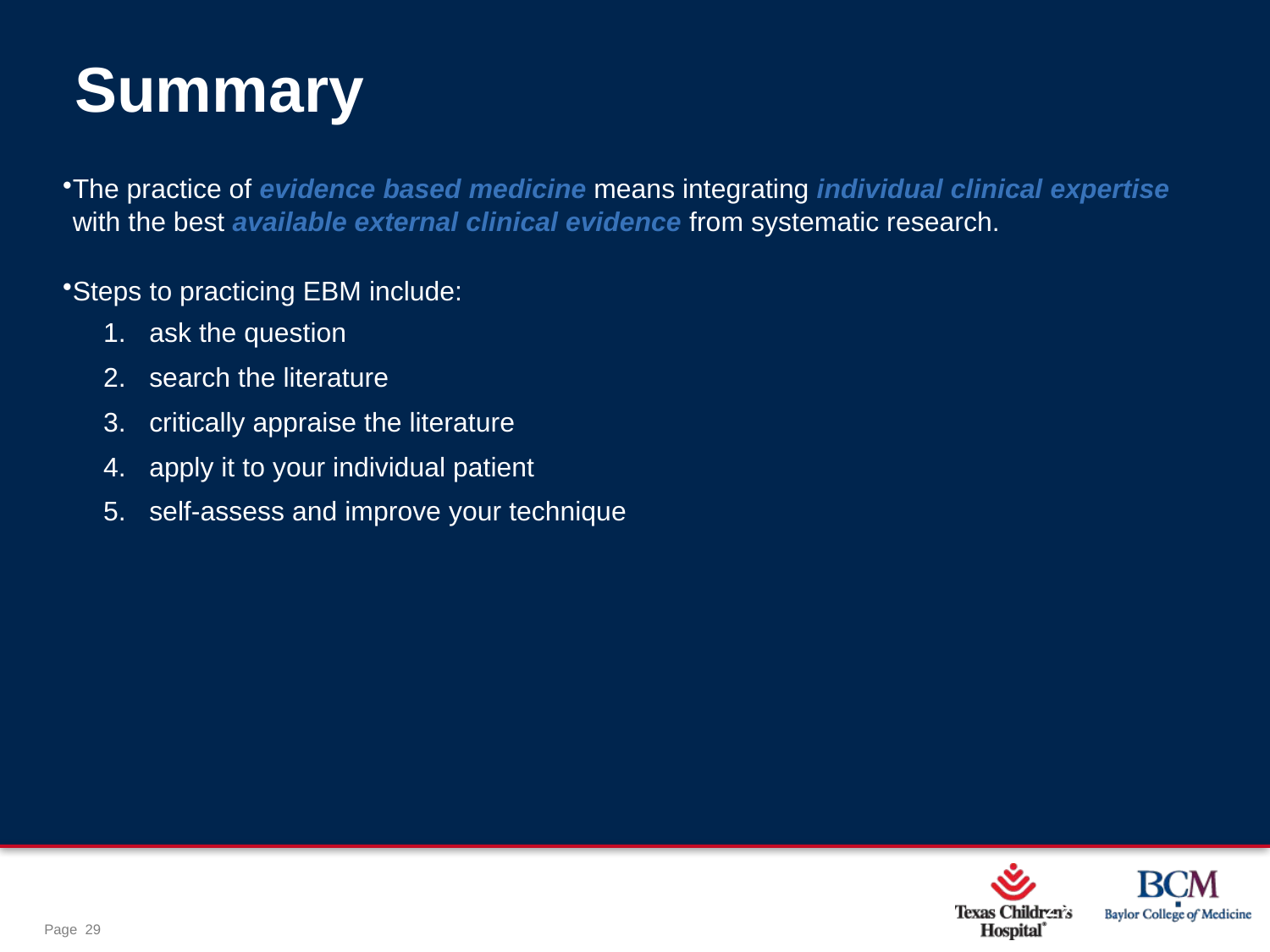

# Summary
The practice of evidence based medicine means integrating individual clinical expertise with the best available external clinical evidence from systematic research.
Steps to practicing EBM include:
ask the question
search the literature
critically appraise the literature
apply it to your individual patient
self-assess and improve your technique
29

## Slide 30
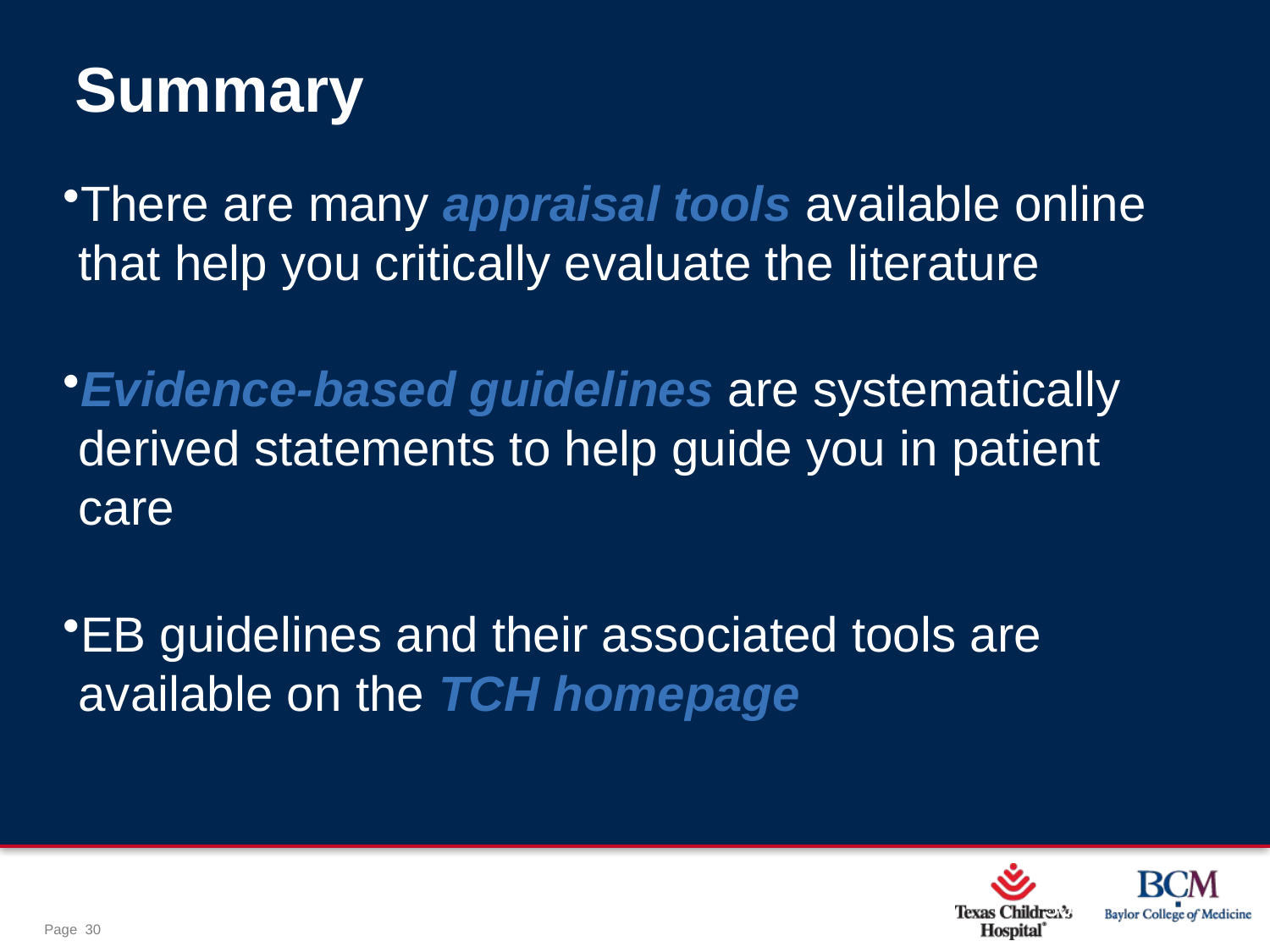

# Summary
There are many appraisal tools available online that help you critically evaluate the literature
Evidence-based guidelines are systematically derived statements to help guide you in patient care
EB guidelines and their associated tools are available on the TCH homepage
30

## Slide 31
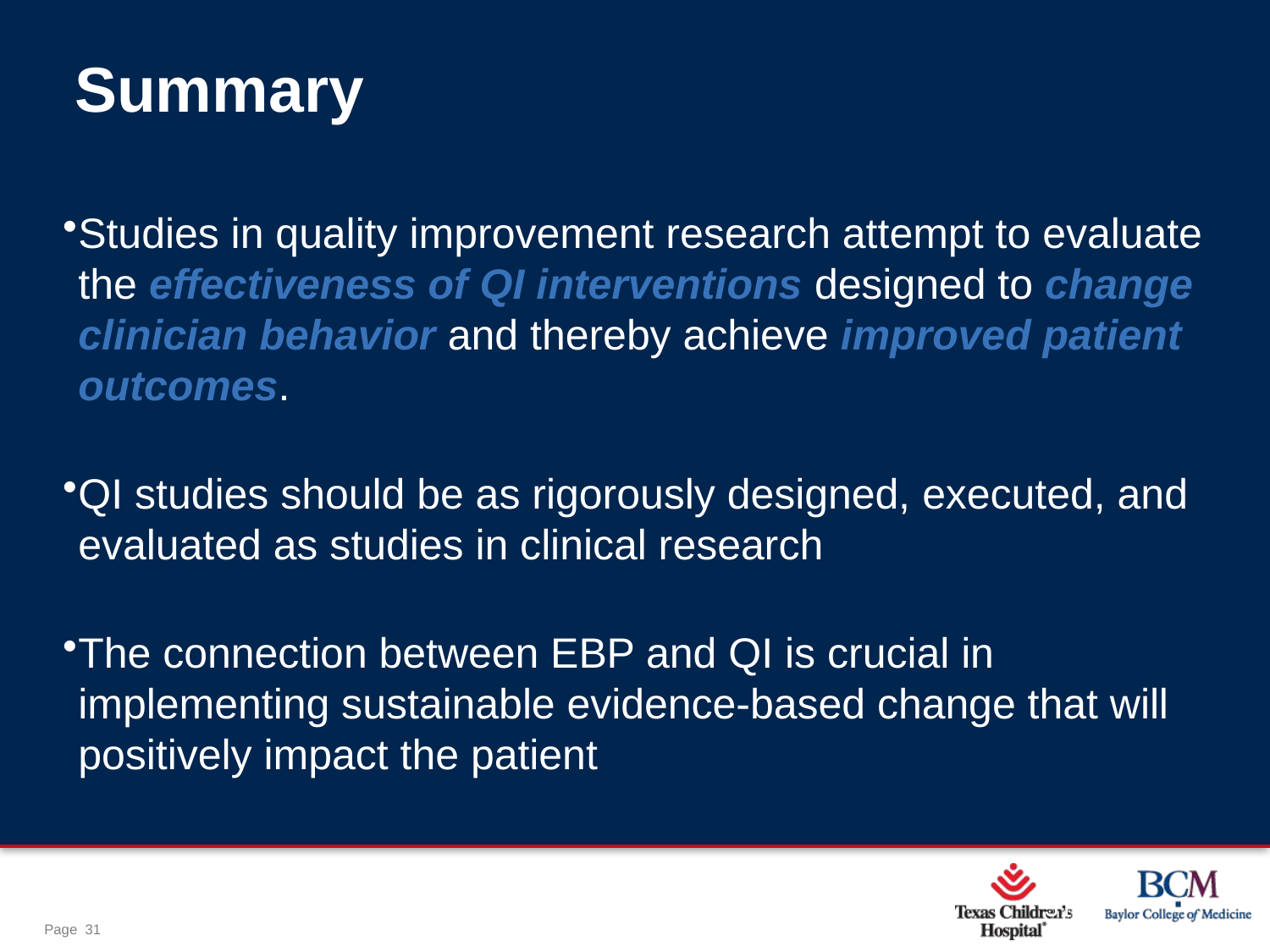

# Summary
Studies in quality improvement research attempt to evaluate the effectiveness of QI interventions designed to change clinician behavior and thereby achieve improved patient outcomes.
QI studies should be as rigorously designed, executed, and evaluated as studies in clinical research
The connection between EBP and QI is crucial in implementing sustainable evidence-based change that will positively impact the patient
31

## Slide 32
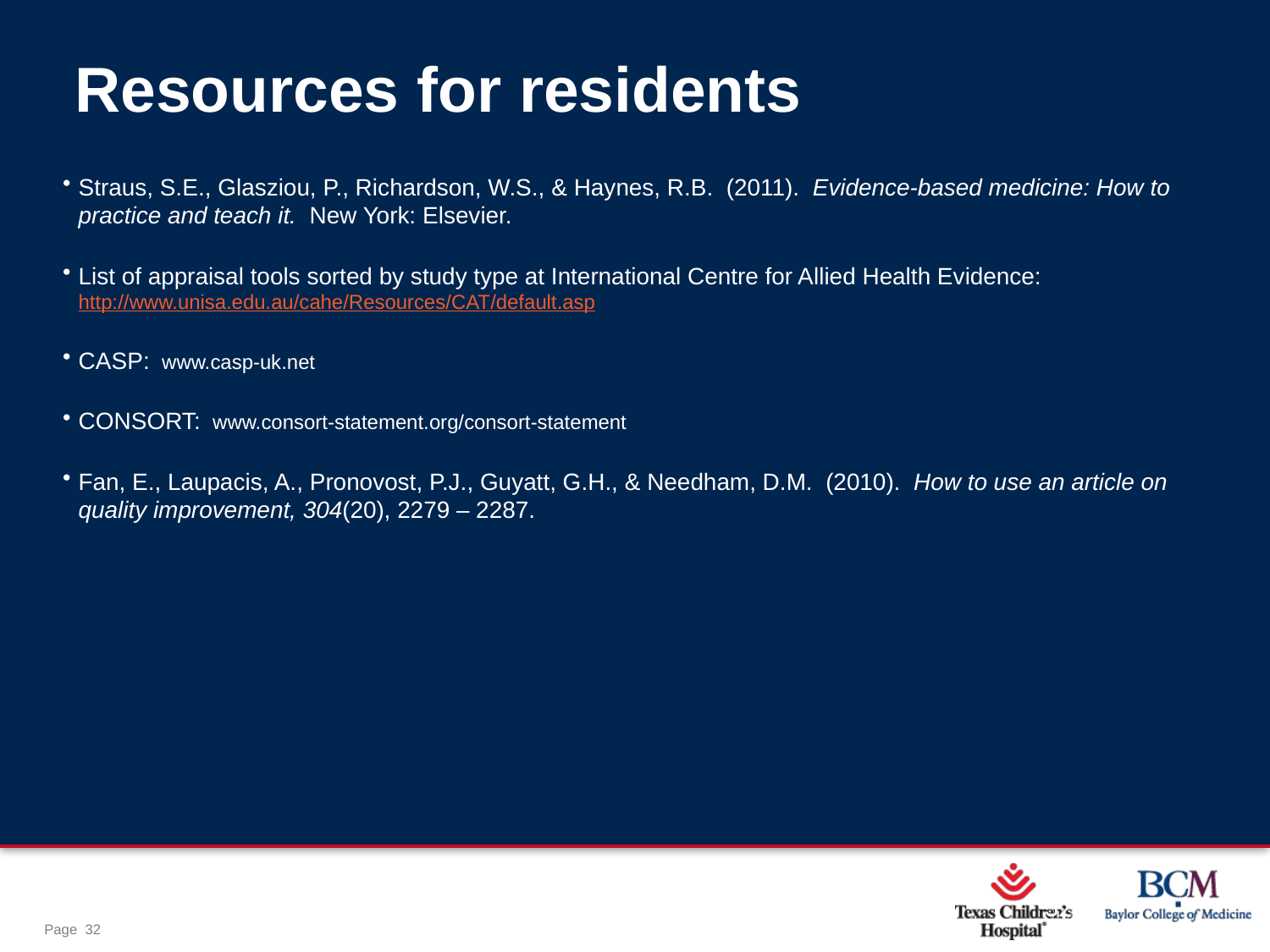

# Resources for residents
Straus, S.E., Glasziou, P., Richardson, W.S., & Haynes, R.B. (2011). Evidence-based medicine: How to practice and teach it. New York: Elsevier.
List of appraisal tools sorted by study type at International Centre for Allied Health Evidence: http://www.unisa.edu.au/cahe/Resources/CAT/default.asp
CASP: www.casp-uk.net
CONSORT: www.consort-statement.org/consort-statement
Fan, E., Laupacis, A., Pronovost, P.J., Guyatt, G.H., & Needham, D.M. (2010). How to use an article on quality improvement, 304(20), 2279 – 2287.
32

## Slide 33
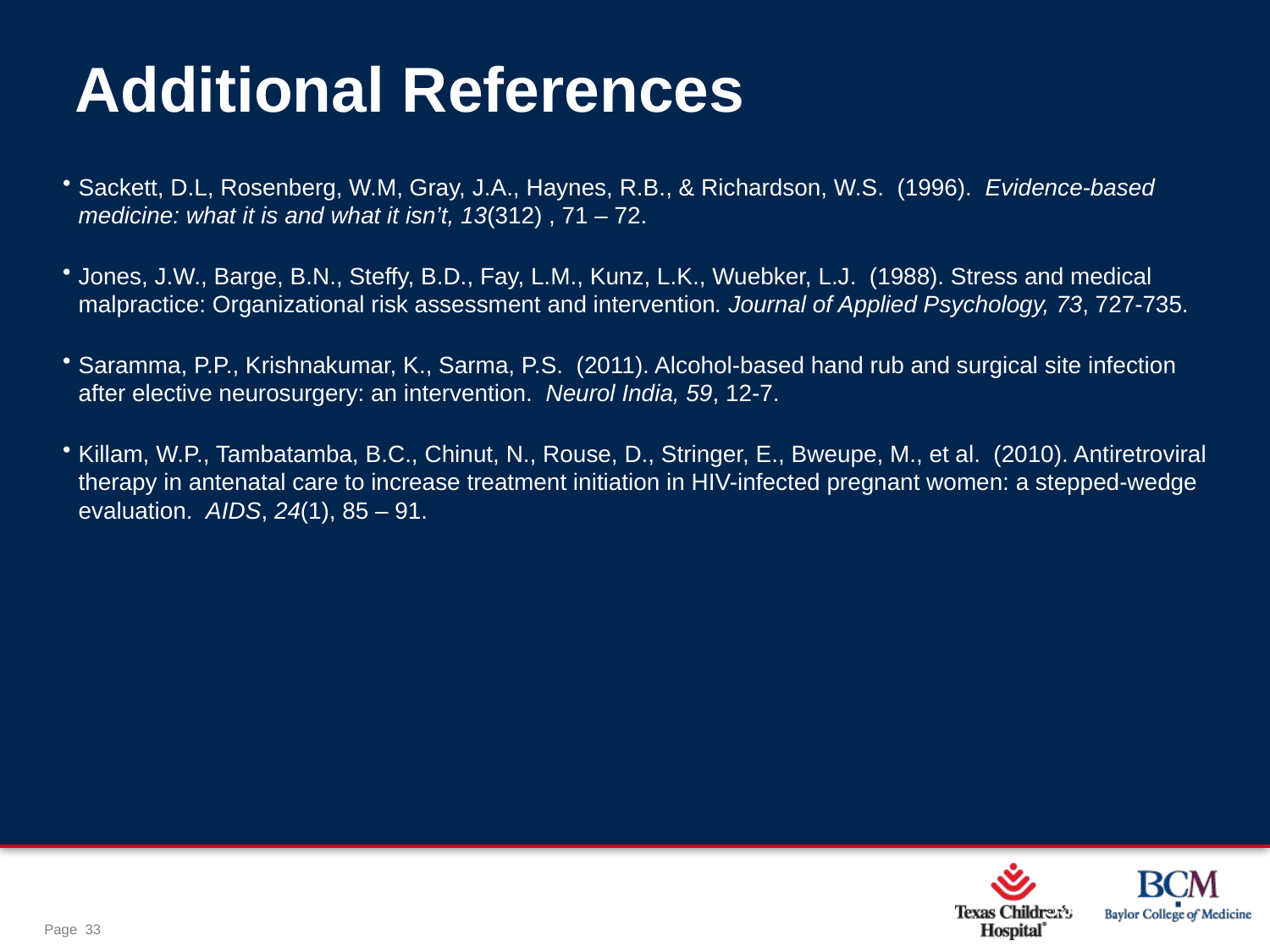

# Additional References
Sackett, D.L, Rosenberg, W.M, Gray, J.A., Haynes, R.B., & Richardson, W.S. (1996). Evidence-based medicine: what it is and what it isn’t, 13(312) , 71 – 72.
Jones, J.W., Barge, B.N., Steffy, B.D., Fay, L.M., Kunz, L.K., Wuebker, L.J. (1988). Stress and medical malpractice: Organizational risk assessment and intervention. Journal of Applied Psychology, 73, 727-735.
Saramma, P.P., Krishnakumar, K., Sarma, P.S. (2011). Alcohol-based hand rub and surgical site infection after elective neurosurgery: an intervention. Neurol India, 59, 12-7.
Killam, W.P., Tambatamba, B.C., Chinut, N., Rouse, D., Stringer, E., Bweupe, M., et al. (2010). Antiretroviral therapy in antenatal care to increase treatment initiation in HIV-infected pregnant women: a stepped-wedge evaluation. AIDS, 24(1), 85 – 91.
33

## Slide 34
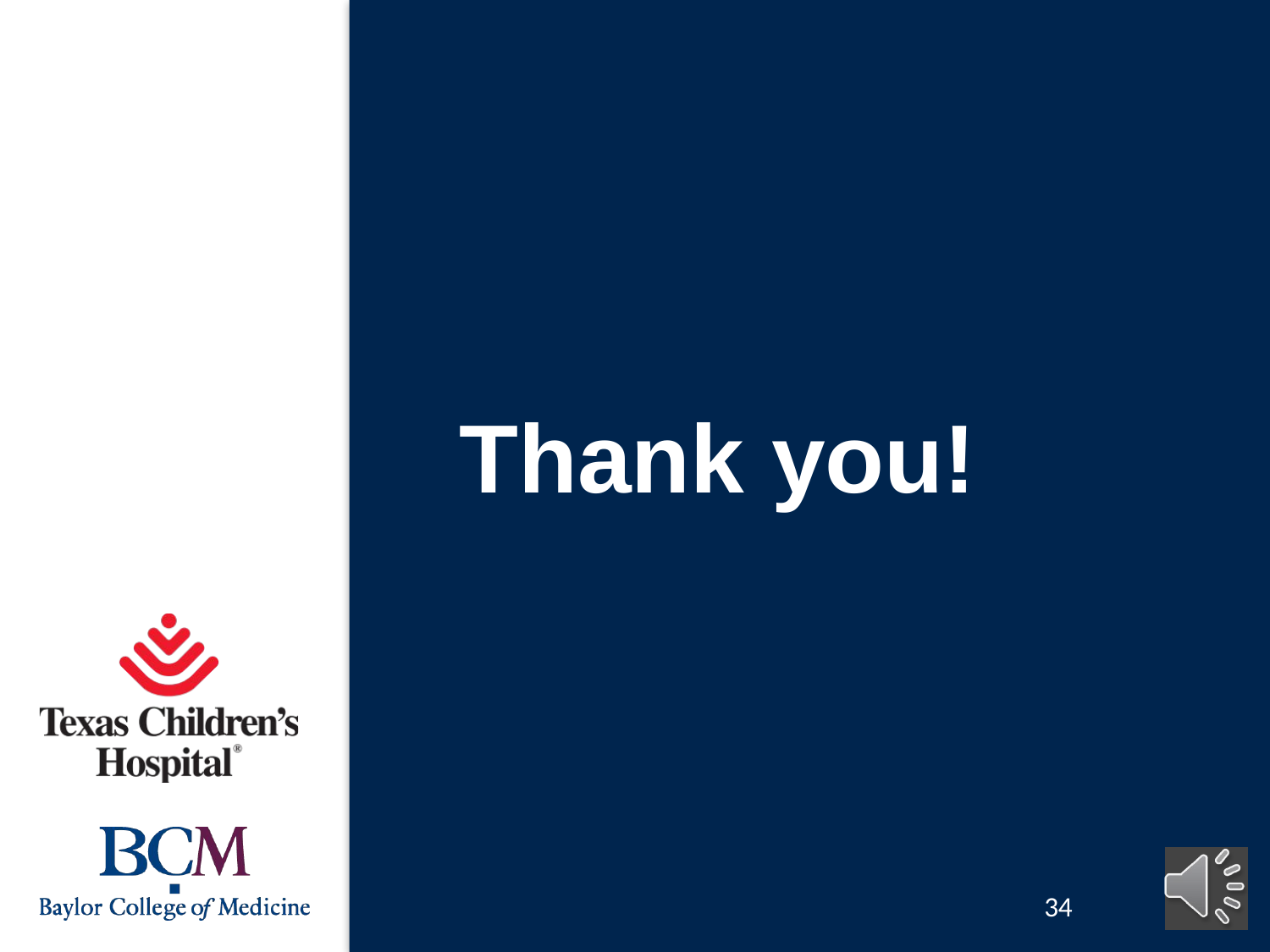

# Thank you!
34
